# Quantitative proteomics reveals coordinated changes in the proteome during replicative senescence

**DOI:** 10.1038/s41467-026-77686-8

**Published:** 2026-09-16

**Authors:** Nádia Da Silva Fernandes, Fridolin Kielisch, Amitkumar Fulzele, Sivarajan Karunanithi, Justus F. Gräf, Jia-Xuan Chen, Christian Behl, Helle D. Ulrich, Petra Beli

**Affiliations:** 1 Institute of Molecular Biology (IMB), Mainz, Germany; 2 Institute of Pathobiochemistry, The Autophagy Lab, University Medical Center of the Johannes Gutenberg University, Mainz, Germany; 3 Institute of Developmental Biology and Neurobiology (IDN), Johannes Gutenberg University, Mainz, Germany

**Keywords:** Senescence, Proteomics, Post-translational modifications

## Abstract

Cellular senescence is a state of irreversible cell cycle arrest triggered by telomere erosion, persistent DNA damage or chronic stress. The accumulation of senescent cells disrupts tissue function and contributes to aging and disease. Here, we employ mass spectrometry-based proteomics to systematically interrogate dynamic proteome changes at multiple levels during the progression of replicative cellular senescence. We demonstrate that proteome changes during senescence occur in a coordinated manner, characterized by widespread protein depletion on chromatin. Moreover, components of the cytoplasmic translation machinery are depleted, while mitochondrial proteins display increased insolubility. Autophagic and proteasome activity is compromised in senescent cells along with remodeling of ubiquitin linkages and depletion of ubiquitin E3 ligases. Comparison of the senescent proteome with different pathophysiological cellular states reveals a distinctive senescent signature shaped by changes in the proteostasis network. Collectively, we provide a resource for the exploration of temporally resolved changes in the senescent proteome.

## Abstract (web summary)

Senescence involves a massive reorganization of cellular components. This study shows that changes in ribosomal proteins and the ubiquitin-proteasome system are distinctive features of replicative senescence when compared to other cellular stresses.

## Introduction

Cellular senescence is a hallmark of aging and a driver of chronic inflammation and age-related diseases^1,2^. Senescent cells accumulate in aged tissues, where they contribute to local tissue dysfunction and chronic inflammation, ultimately leading to detrimental systemic effects. Although senescent cells display heterogenous characteristics, they share certain features, including an irreversible cell cycle arrest, resistance to apoptosis, secretion of inflammatory cytokines known as the senescence-associated secretory phenotype (SASP)^1,2^, and the expression of senescence markers such as the p16INK4a/Rb and/or p53/p21CIP1 tumor suppressors. Senescent cells also share an enlarged and flattened morphology^1,3^. Beyond replicative senescence – which results from telomere shortening and an associated sustained DNA damage response – senescence can also be triggered by chronic stress, mitochondrial dysfunction and oncogene activation^1^.

To treat age-related diseases and extend health span, efforts are ongoing to develop senolytic and senomorphic drugs that selectively target and destroy senescent cells through induction of apoptosis or suppression of the SASP phenotype, respectively^4^. Mechanistic understanding of senescence-specific vulnerabilities and the identification of new senolytic targets require a detailed characterization of changes that accompany senescence and their consequences on different cellular processes. Previous studies have investigated changes in the transcriptome of human cells during senescence, resulting in the identification of common markers of senescence^5–8^. However, detailed characterization of the changes in the proteome and associated post-translational modifications (PTMs) during replicative senescence with temporal resolution is still lacking.

The requirement for degradation of damaged proteins and cellular organelles is expected to increase during aging and senescence. The activities and the levels of the major cellular degradation machineries, the ubiquitin-proteasome system (UPS) and the autophagy-lysosome pathway, remain debated, also owing to the use of different cellular and animal aging model systems^9^. Modification of proteins with ubiquitin plays a central role in these degradation pathways^10^. Research in recent years has provided insights into the complexity of ubiquitin signaling via different homotypic, heterotypic or branched ubiquitin chains^11^. Consequently, mechanistic understanding of how ubiquitylation pathways are altered during aging and senescence requires investigations of ubiquitin in a linkage-specific manner.

Here, we employed mass spectrometry (MS)-based proteomics to determine changes in the proteome, the ubiquitin- and SUMO2/3-modified proteome, as well as protein solubility during replicative senescence in cultured human lung fibroblasts (IMR90). We found that senescence is accompanied by a steady depletion of nucleoplasm- and chromatin-associated proteins, except for core histones and DNA replication proteins, which were depleted late in the process. Levels of the protein components of the autophagy-lysosomal pathway increased, even though the general autophagic activity was compromised in senescent cells. Similarly, core proteasome subunits, especially subunits of the immunoproteasome, increased in abundance. In contrast, proteins involved in translation, including cytoplasmic ribosome subunits, showed a steady depletion during senescence. We demonstrated that senescence is associated with decreased solubility of > 500 proteins, with mitochondrial ribosomes and other mitochondria-localized proteins being overrepresented in the insoluble proteome. Finally, by comparing the proteome response during replicative senescence with responses to other stress conditions such as chronic proteasome and HSP90 chaperone inhibition, oxidative stress and reversible cell cycle arrest, we demonstrate that the replicative senescence proteome displays a characteristic signature that only partially overlaps with other pathological or physiological cellular states.

Our longitudinal multi-omics analysis during replicative senescence revealed specific rewiring of homeostatic pathways and cellular compartments, providing insights into the molecular mechanisms underlying senescence. These findings illustrate the temporal dynamics of regulatory network perturbations that accompany senescent phenotypes, opening new avenues for targeted therapeutic interventions.

## Results

### Steady depletion of chromatin-associated proteins is characteristic for replicative senescence

**Fig. 1:**
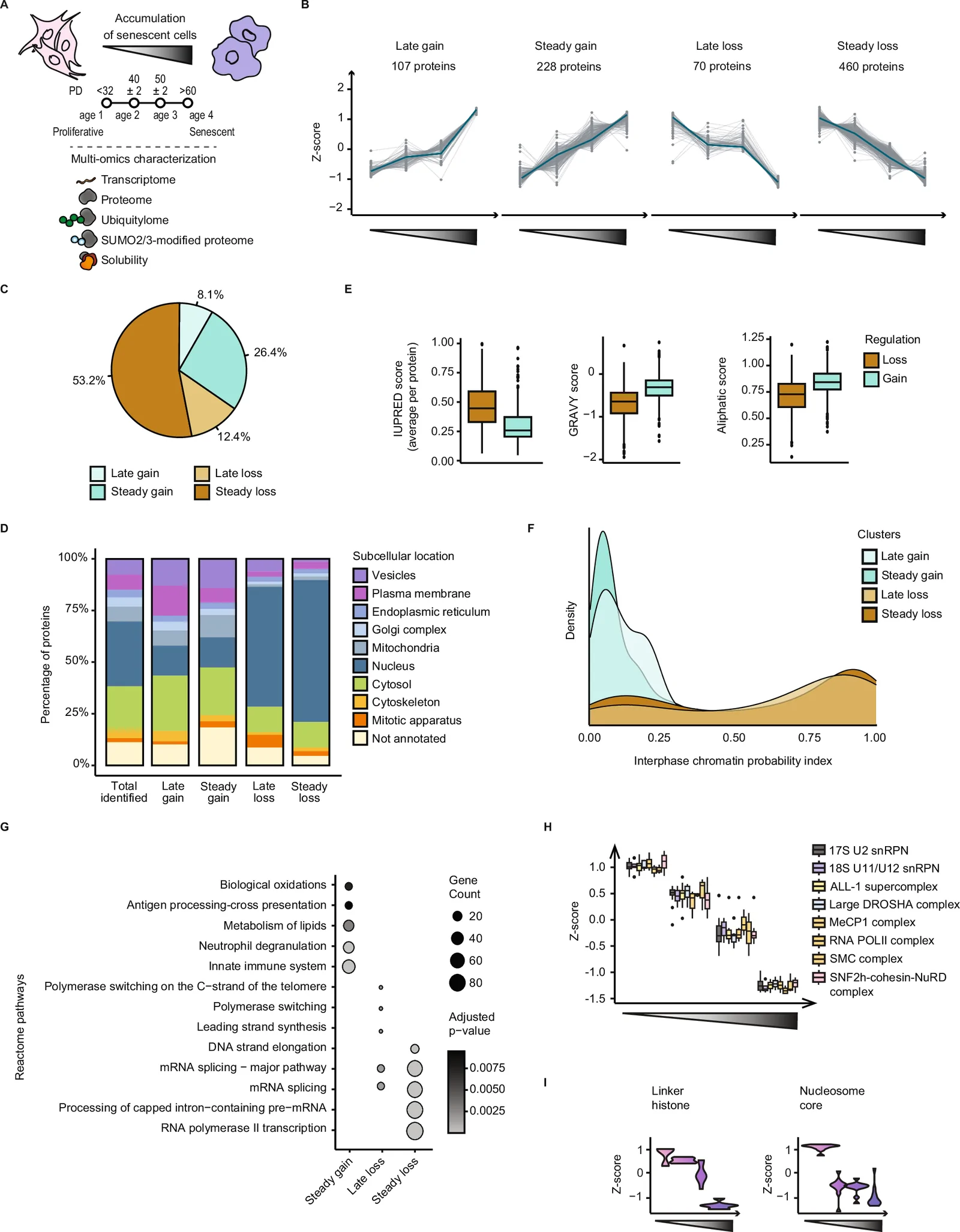
Replicative cellular senescence results in a coordinated depletion of chromatin-associated proteins. **A** Schematic representation of the experimental approach to analyze the transcriptome, proteome, ubiquitin- and SUMO2/3-modified proteome of IMR90 fibroblasts upon senescence in vitro. Senescence is represented by a gradient from white (proliferative) to black (senescent). All procedures involved either early-passage (age 1, proliferative) or late-passage (age 4, senescent) IMR90 cells, representing cumulative population doublings of <32 or >60, respectively. Time-course experiments involved two intermediate stages (age 2 and age 3) with cumulative population doublings of 40 and 50 ± 2, respectively. For each stage, a minimum of three age replicates was analyzed. **B** Differentially regulated proteins were clustered using the K-means algorithm, showing distinct temporal patterns. Proteomes were acquired for three distinct growth curves (GC1, GC2, GC3), each stage represented by four age replicates. **C** Pie plot displaying the percentage of regulated proteins assigned to the clusters defined above. **D** Stacked bar plot showing the distribution of protein main subcellular localization, as defined by the Human Proteome Atlas, organized by the clusters defined above. **E** Biochemical properties of proteins differentially regulated during senescence, for three GCs, each stage represented by four age replicates (IUPred: intrinsic disorder; GRAVY: grand average of hydropathy, Aliphatic: relative volume occupied by aliphatic amino acids). Bottom, interior line, and top of the box represent first quartile, median and third quartile, respectively. Whiskers represent minimum and maximum values within 1.5× interquartile range (IQR), with outliers indicated. **F** Distribution of interphase chromatin probability index, as defined by Kustatscher et al.^15^, organized by the protein clusters defined above. **G** Bubble plot showing significantly enriched reactome terms (*p* < 0.05, one-sided hypergeometric test with Benjamini-Hochberg false discovery rate correction) for all differentially regulated proteins present in the clusters defined above. Only the top five terms are shown for each cluster. **H** Box plot representing Z-scores for distinct nuclear complexes, annotated by the CORUM mammalian complex annotation^12,13^. Data derived from 3 GCs, each stage represented by four age replicates. Bottom, interior line, and top of the box represent first quartile, median and third quartile, respectively. **I** Violin plots displaying Z-scores of all quantified linker and core histones during senescence.

To study changes across the transcriptome, proteome, and ubiquitin- and SUMO2/3-modified proteomes, we used the IMR90 human fibroblast model of replicative senescence^12^. We selected four consecutive stages corresponding to defined population doublings (PD < 32, 40 ± 2, 50 ± 2 and >60, in the following, termed age 1-4), which represent a progressively increasing fraction of senescent cells. As part of our focus on the proteostasis network, we also monitored alterations in protein solubility that may occur due to protein damage and/or aggregation (Fig. 1A and Supplementary Fig. 1A).

We quantified the relative abundance based on equal total protein amounts of 5,923 proteins extracted using a modified RIPA buffer (containing 1% NP-40 and 0.1% sodium deoxycholate), of which 14% showed significant changes during senescence (Fig. 1B and Supplementary Data 1). Notably, these changes followed distinct temporal patterns that were also observable on the transcriptome level (Supplementary Fig. 1B and Supplementary Data 2), suggesting that the processes triggered by the accumulation of senescent cells are not stochastic (Fig. 1B). While the largest fraction of differentially regulated proteins exhibited a steady loss or a steady gain during senescence (460 and 228 proteins, respectively), a smaller fraction of proteins increased or decreased in their levels only at later stages. Overall, 65% of the differentially regulated proteins decreased in abundance, demonstrating that protein depletion is a prevalent feature of cellular senescence (Fig. 1C). To understand whether protein depletion displayed biases to specific cellular compartments, we used the annotation from the Human Protein Atlas^13^. Interestingly, we found that ~two-thirds of significantly depleted proteins localized to the nucleus (Fig. 1D). Notably, depletion of nuclear proteins was apparent on the proteome but not on the transcriptome level (Supplementary Fig. 1C, D).

Generally, nuclear and chromatin-binding proteins tend to exhibit a high degree of disorder and low hydrophobicity in comparison to membranous and vesicular proteins^14^. Consistent with this, we observed a higher degree of disorder and lower hydrophobicity among the depleted proteins compared to those that increased in abundance (Fig. 1E). We also found that more than half of the depleted proteins are likely to associate with chromatin (Fig. 1F), given that they display an “interphase chromatin probability index”^15^ larger than 0.7. Notably, depletion of nuclear proteins occurred in a coordinated manner: protein complexes involved in transcription, chromatin organization and RNA processing (Fig. 1G, H and Supplementary Fig. 1E), such as RNA polymerase II, the SMC complexes and the 17S U2 snRNP, respectively, were lost earlier than proteins involved in DNA replication and replication stress, such as POLA1, RFC3, RFC5, PRIM and CHEK1 (Fig. 1G and Supplementary Fig. 1F). A similar coordination applied to the most abundant class of DNA-binding proteins–histones–where an early depletion of all core histones, but a late loss of H1 linker histones was observed (Fig. 1I). Given that proliferative and senescent cells differ in morphology and size and consequently in total protein content, we repeated the proteome analysis based on equal cell numbers and utilizing a full RIPA buffer (containing 1% SDS, 1% NP-40 and 0.1% sodium deoxycholate) to ensure total protein solubilization (Supplementary Fig. 1G and Supplementary Data 1). Consistent with initial findings, we observed a depletion of nuclear and chromatin-associated proteins in senescent cells (Supplementary Fig. 1H, I). These temporal distinctions imply that chromatin reorganization follows, at least in part, a regulated scheme rather than arising as a stochastic consequence of cellular dysfunction.

### Changes in the transcription and translation protein network during senescence

**Fig. 2:**
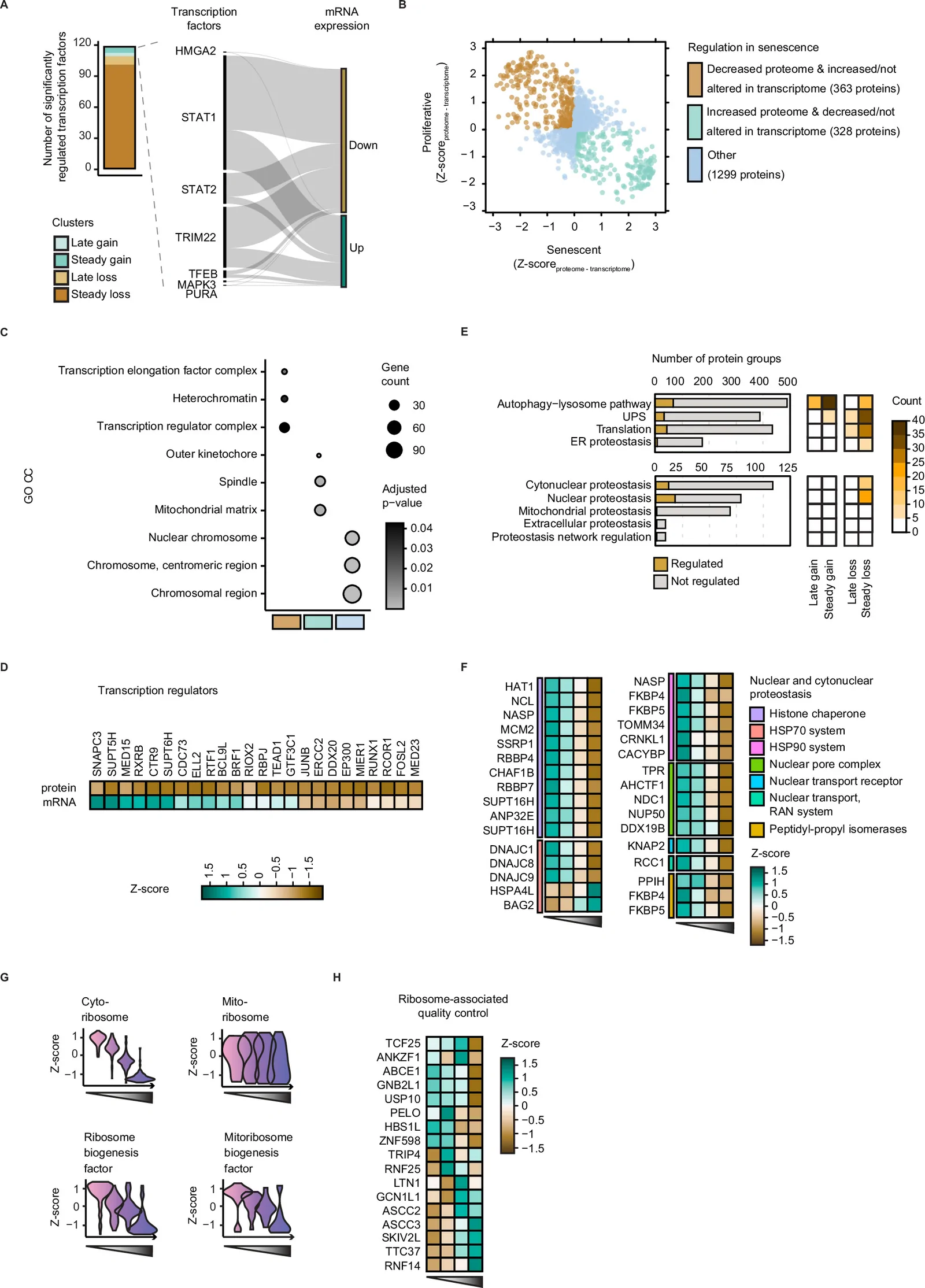
Alterations in the transcription and proteostasis network during cellular replicative senescence. **A** Left: Stack plot showing the number of transcription factors significantly regulated at the proteome level, arranged according to the clusters defined in Fig. 1A. Right: Sankey diagram representing the targets of upregulated transcription factors, as defined by the TFLink^16^ gateway, at the transcriptome level. The transcriptome was acquired for three growth curves, each stage represented by one age replicate of each growth curve. **B** Lattice plot correlating the Z-score differences between proteome and transcriptome of proteins found to be differentially regulated in senescence. Proteins and transcripts labeled as “Other” exhibit similar trends at protein and mRNA levels. **C** GO Cellular Compartment (CC) term enrichment for the proteins/transcripts and the clusters defined in panel (**B**). Adjusted *p* values below 0.05 were considered statistically significant (one-sided hypergeometric test with Benjamini-Hochberg false discovery rate correction). **D** Heatmap showing mRNA and protein levels of those transcription-associated factors that were depleted at the protein level, while transcripts were not significantly altered during senescence. **E** Bar plot showing the number of significantly regulated proteins in each proteostasis network category and the number of proteins found in each cluster. **F** Heatmap showing Z-scores of components of the nuclear and cytonuclear proteostasis network, found to be significantly regulated in senescence. Proteins are grouped into categories as classified by the proteostasis consortium annotation. **G** Violin plots showing the Z-score of cyto- and mitoribosome subunits, as well as the corresponding biogenesis factors. **H** Heatmap showing Z-scores of significantly regulated proteins associated with ribosome-associated quality control.

To understand the molecular mechanisms underlying the observed changes in protein abundance, we focused on transcription and proteostasis. In the context of transcription regulation, we observed that 117 transcription factors (as defined by the TFlink gateway^16^) showed significant changes during senescence; however, only seven were found to increase in abundance (Fig. 2A). These included STAT1 and STAT2, which maintain and reinforce the senescence^17,18^, as well as TFEB and HMGA2, which are responsible for increased lysosome biogenesis^19^ and SASP modulation^20^ during senescence, respectively. Nevertheless, the mRNA levels of more than two-thirds of their respective target genes were downregulated, demonstrating that despite an increase in abundance, these transcription factors are unable to fully activate the expression of target genes in senescent cells (Fig. 2A).

Furthermore, 9.5% of the proteins undergoing significant changes in their abundance during replicative senescence showed no correlation between mRNA and protein levels (Fig. 2B). Interestingly, among the proteins that were depleted in senescence despite an upregulation of their mRNAs were components of the complexes involved in the regulation of RNA polymerase II-mediated transcription elongation, such as the PAF complex, DSIF complex and super elongation complex (SEC) (Fig. 2B–D). In contrast, mitochondrial matrix proteins increased in their levels even though the corresponding mRNA transcripts showed the opposite trend (Fig. 2B, C).

The discordance between the transcriptome and proteome led us to focus on the proteostasis network that includes protein folding, synthesis/translation and degradation/recycling. Our analysis yielded quantitative information on 53.8% of proteins associated with proteostasis, based on the annotation from the Proteostasis Consortium^21,22^. Ten percent of these showed significant changes in their levels (Fig. 2E), suggesting a fine-tuned regulation of proteostasis during senescence. In line with previous observations^23,24^ that nuclear proteins are depleted during senescence, we found significant changes in >20% and 11% of proteins associated with nuclear and cytonuclear proteostasis, respectively (Fig. 2F). This includes histone chaperones, the nuclear pore complex, and nuclear transport machinery components, suggestive of a dysregulated nuclear homeostasis during senescence (Fig. 2F). Interestingly, we observed that co-chaperones of the HSP70 system exhibited opposing behavior: BAG2 and HSPA4L, which function as nucleotide exchange factors^25,26^, showed increased levels during replicative senescence, whereas members of the DNAJ family, known to stimulate HSP70 ATPase activity^27^, displayed decreased levels.

Furthermore, we observed a significant depletion of 6.7% of proteins associated with translation. In particular, the cytoplasmic ribosome and ribosome biogenesis factors decreased in abundance (Fig. 2G). Ribosome maturation factor SBDS was the only ribosome biogenesis factor that increased in abundance during senescence (Supplementary Fig. 2A). SBDS acts as a sensor of ribosomal stress, tying this type of stress response to cell cycle regulation through the MDM2-p53 loop^28^. Notably, we observed an increase in the abundance of the stalled ribosome sensor GCN1, the associated ubiquitin E3 ligase RNF14, and members of the SKI and ASC complexes involved in the resolution of ribosome stalling events^29,30^ (Fig. 2H).

### Distinct effects of senescence on the autophagy-lysosome pathway and ubiquitin-proteasome system components

**Fig. 3:**
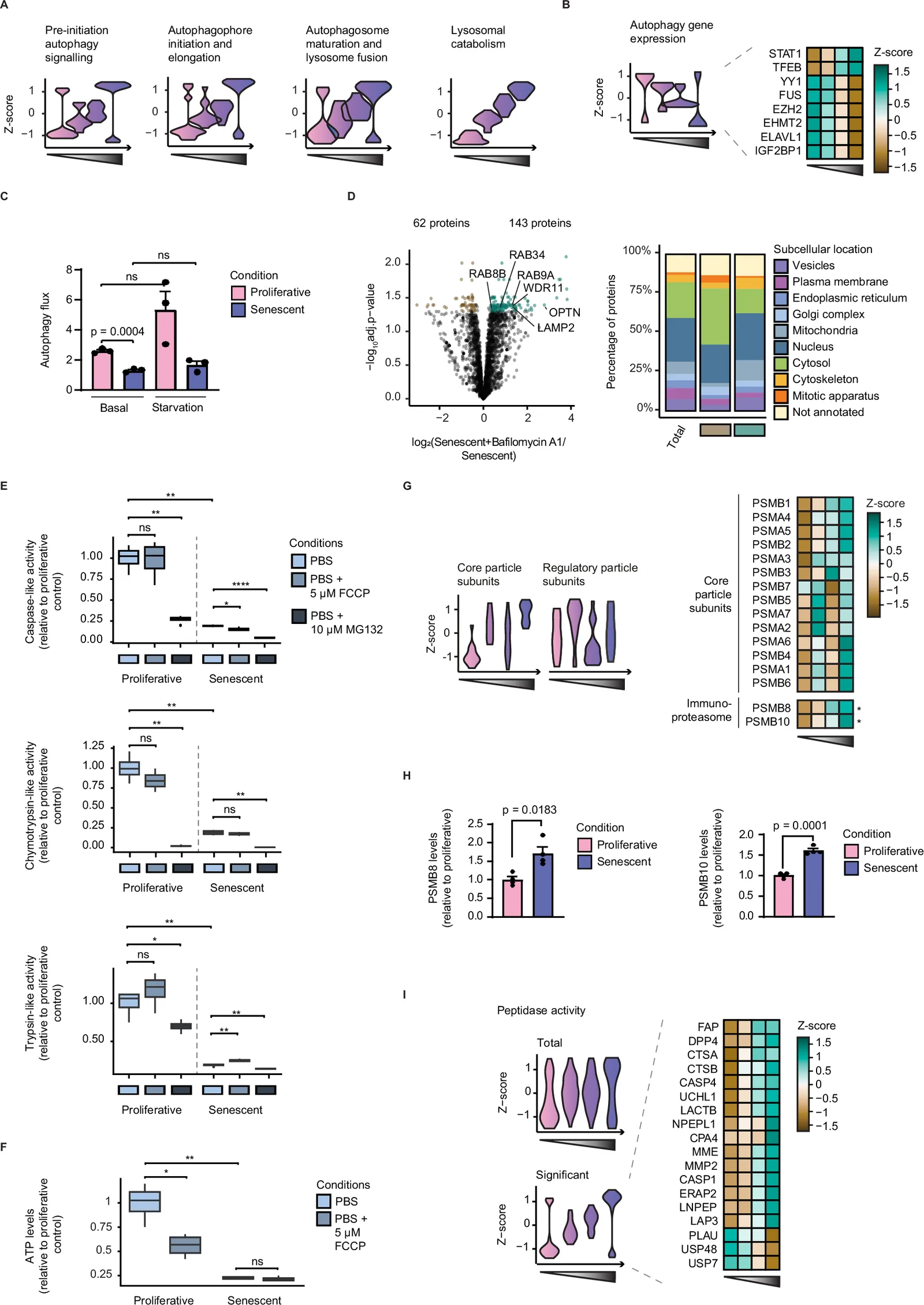
Autophagy and proteasomal degradation are compromised in replicative senescence. **A** Violin plots showing Z-scores of all significantly changing proteins associated with specific autophagy phases. **B** Violin plot and heatmap showing levels of autophagy-related transcription factors significantly altered in senescence. **C** Autophagy flux, quantified by comparing levels of lipidated (LC3-II) to non-lipidated LC3B (LC3-I) in proliferative and senescent cells upon 2.5 µM Bafilomycin A1 exposure for 2 h, in control and starvation conditions. LC3B levels were normalized to GAPDH. Bar plot values represent mean ± SEM (GC4, *n*=three age replicates). Representative blots are shown in Supplementary Fig. 2B. **D** Left: Volcano plot showing all quantified and significantly regulated proteins in senescent cells upon 2.5 µM Bafilomycin A1 exposure for 2 h. Selected proteins involved in intracellular trafficking and autophagy are highlighted. (GC4, *n*=three age replicates) Right: Stacked bar plot illustrating the distribution of protein main subcellular localization, as defined by the Human Proteome Atlas. **E** Box plot showing proteasomal activity of cells under starvation conditions, normalized to proliferative cells, measured by luminescence. Error bars represent SEM. Bottom, interior line, and top of the box represent first quartile, median and third quartile, respectively. Statistical analysis was performed using multiple unpaired *t* test with Welch’s correction. *p < 0.05, **p < 0.01, ***p < 0.001, **** *p* < 0.0001, *ns* not significant. (GC2, *n*=four age replicates) (**F**) Box plot showing relative ATP levels in lysates of cells cultured in starvation conditions, measured by luminescence. Error bars represent SEM. Bottom, interior line, and top of the box represent first quartile, median and third quartile, respectively. Statistical analysis was performed using multiple unpaired t-test with Welch’s correction. *p < 0.05, **p < 0.01, *ns* not significant. (GC2, *n*=four age replicates) (G) Left: Violin plot showing Z-scores for the abundance of all quantified core and regulatory proteasome subunits. Right: Heatmaps showing Z-scores of core particle and immunoproteasome subunits (asterisks: significant changes). **H** Quantification of western blots of PSMB8 and PSMB10 in proliferative and senescent cells. Signals were normalized to Ponceau staining. Statistical analysis was performed using multiple unpaired t-test with Welch’s correction. Bar plot values represent mean ± SEM (GC1, *n*= four age replicates). Representative blots are shown in Supplementary Fig. 2E. **I** Left: Violin plot showing Z-scores of all quantified and significantly regulated proteins with peptidase activity. Right: Heatmap highlighting peptidases found to be differentially regulated in senescence.

Among the components of the main degradative pathways, the UPS and autophagy, 9.9% and 13.7% of proteins associated with these pathways underwent significant changes in their abundance, respectively. However, while protein depletion prevailed in the UPS, in particular ubiquitin E3 ligases, components of the autophagy system tended to increase in abundance; with >70% of the significantly regulated autophagy-associated proteins showing increased levels during senescence (Fig. 3A). This set includes proteins participating in the early (pre-initiation, initiation and elongation) and late stages (lysosomal metabolism and autophagosome maturation) of autophagy. Yet, proteins responsible for the transcription of autophagy genes, such as FUS, ELAVL1 and EZH2, were found to be depleted (Fig. 3B). Transcription factor TFEB, which plays a central role in promoting transcription of lysosomal biogenesis and autophagy genes^31^, increased in abundance. Despite increases in autophagy protein levels, we observed a decreased autophagy flux in senescent cells, assessed by lipidation of LC3B (Fig. 3C and Supplementary Fig. 2B). Thus, it is conceivable that the observed accumulation of autophagy-associated proteins during senescence results from their inefficient degradation alongside the autophagy cargo.

In line with this observation, blocking autophagy through inhibition of lysosome-autophagosome fusion with Bafilomycin A1 induced mild changes in the senescent proteome. Only 6.8% of proteins significantly changed their levels upon short-term autophagy inhibition (Fig. 3D). Analysis of 143 proteins that increased in their abundance after autophagy inhibition revealed a significant enrichment of mitochondrial proteins, such as UQCRFS1, ATP5F1A, ATP5F1B, NDUFS3 and NNT, involved in oxidative phosphorylation, indicating that mitochondria are a major autophagic cargo in senescent cells (Fig. 3D). Additionally, this group included proteins involved in intracellular trafficking and lysosomal pathways, such as members of the RAB family, as well as autophagy receptors such as OPTN (Fig. 3D).

To evaluate whether proteasome activity could compensate for the impairment of autophagy, we analyzed the activity and protein levels of the ubiquitin-proteasome system. 20S proteasome activity assays in crude lysates showed a significant reduction of chymotrypsin-, trypsin- and caspase-like activities (Fig. 3E). At the same time, senescent cells exhibited a 4.4-fold reduction of ATP levels (Fig. 3F) compared to proliferative cells. To understand whether the observed reduction in proteasome activity was due to this phenomenon, we repeated the activity assays upon treatment of cells with carbonyl cyanide-4-(trifluoromethoxy)phenylhydrazone (FCCP) to prevent the production of ATP by oxidative phosphorylation^32^. FCCP treatment reduced ATP levels by 50% in proliferative but not in senescent cells (Fig. 3F). These findings are in line with previous reports showing that senescent cells undergo a metabolic shift towards glycolysis^28^ to offset the reduction of ATP production resulting from mitochondrial dysfunction. Indeed, we observed a general increase in abundance of proteins associated with the glycolytic pathway during senescence (Supplementary Fig. 2C), as previously reported^33^. While FCCP treatment did not affect proteasome activity in proliferative cells, it induced subtle but statistically significant changes in the caspase-like and trypsin-like activities in senescent cells, however, in opposite directions (Fig. 3E). Considering these data, we conclude that loss of proteasomal activity in senescent cells is unlikely to be a consequence of ATP depletion.

Decline of proteasomal activity was accompanied by a remodeling of the core and regulatory proteasome particles. We observed an overall increase in abundance of core particle subunits, while levels of the regulatory particle subunits did not significantly change during senescence (Fig. 3G and Supplementary Fig. 2D, E). Notably, we detected a significant increase in abundance of the immunoproteasome subunits—PSMB8 and PSMB10—that was confirmed by western blotting (Fig. 3H and Supplementary Fig. 2F). Proteolysis is also assured by the action of proteases. Among one specific class of proteases–peptidases–we observed a significant increase of ATP-independent peptidases in senescent cells (Fig. 3I), suggesting that senescent cells favor ATP-independent proteolysis.

### SUMO2/3-modified proteins accumulate during senescence

**Fig. 4:**
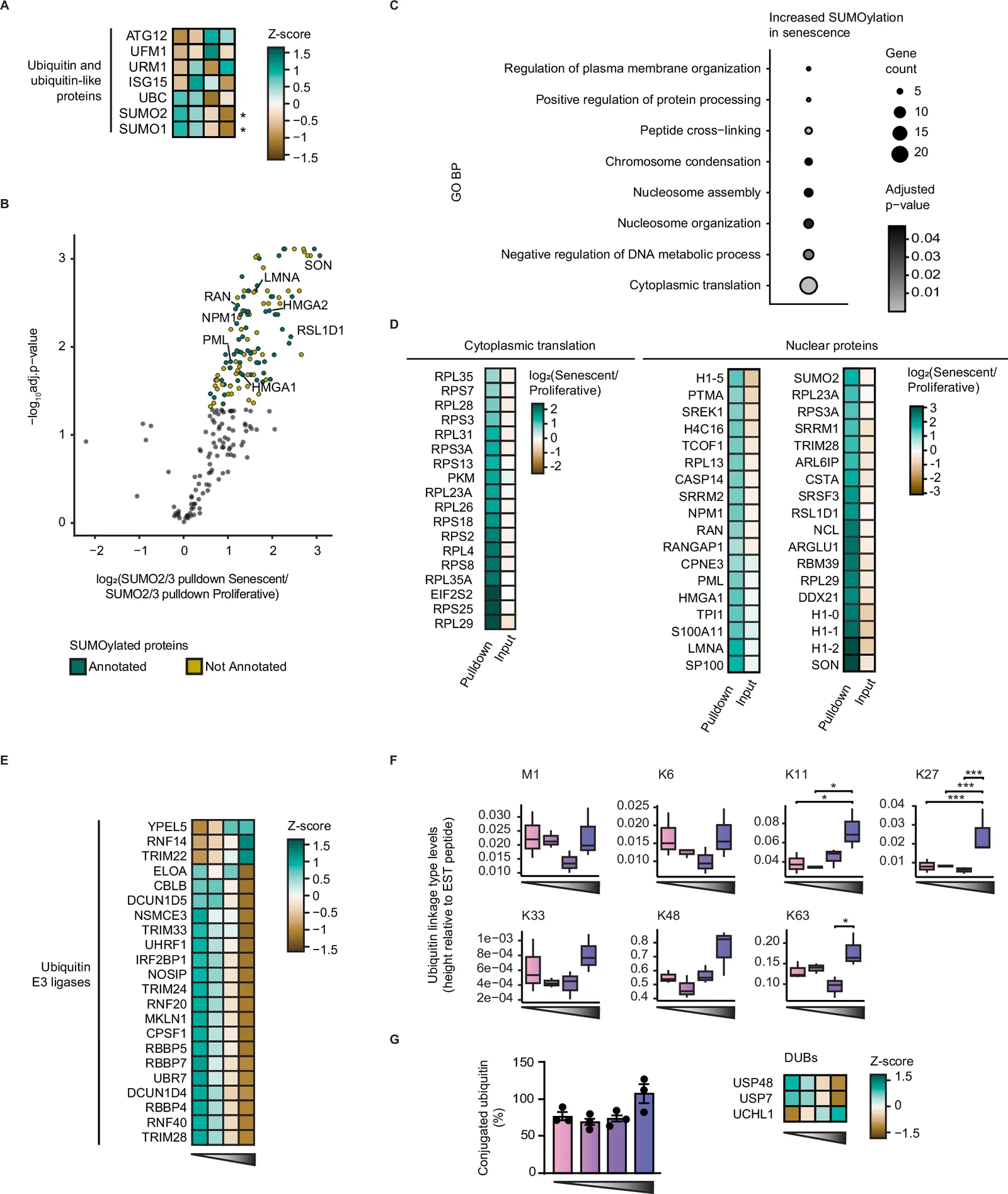
SUMO2/3-modified proteins as well as K11 and K27 ubiquitin linkages accumulate in replicative senescence. **A** Heatmap shows Z-scores of ubiquitin and ubiquitin-like proteins. Asterisks represent significant regulation during senescence. **B** Volcano plot exhibiting all proteins quantified in a SUMO2/3 pulldown. Significantly regulated proteins that are known SUMO2/3 targets based Hendriks and Vertegaal^116^ are color-coded. Proteins associated with cellular senescence are highlighted (GC1, *n*= four age replicates). **C** Dot plot showing GO BP terms enriched for proteins with increased SUMOylation in senescence with an adjusted *p* value below 0.05 (one-sided hypergeometric test with Benjamini-Hochberg false discovery rate correction). Top eight terms are shown. **D** Heatmaps showing the log_2_(Senescent/Proliferative) ratio of proteins associated with cytoplasmic translation (left) and nuclear organization (right) in input and SUMO2/3 pulldowns. **E** Heatmap showing the Z-scores of all E3 ligases found to be differentially regulated during senescence. **F** SureQuant^TM^ targeted quantification of ubiquitin linkages. The EST peptide provides a quantitative measure of total ubiquitin levels. Statistical analysis relied on a moderated *t*-test, with incorporated Bayes variance moderation. *P* values were corrected for multiple testing using the Holm method. ***p < 0.001, **p < 0.01, *p < 0.05. Bottom, interior line, and top of the box represent first quartile, median and third quartile, respectively. Whiskers represent minimum and maximum values within 1.5× IQR, with outliers indicated. (GC4, *n*= three age replicates, seven transitions quantified per age replicate and peptide). **G** Left: Bar plot showing the percentage of conjugated ubiquitin, calculated by the sum of all quantified linkages presented in panel (**F**) and normalized to the total amount of ubiquitin defined by the EST peptide. Error bars represent SEM. (GC4, *n*= three age replicates, seven transitions quantified per age replicate and peptide). Right: Heatmap showing the Z-score of deubiquitylating enzymes whose levels changed significantly during senescence.

Considering the changes in autophagy and the UPS, we investigated the effects of cellular senescence on ubiquitin and ubiquitin-like modification systems. We found that SUMO1 and SUMO2/3 exhibited significant depletion (Fig. 4A), alongside a tendential decrease of SUMO-activating and E3 ligase enzymes (Supplementary Fig. 3A), while the abundance of other ubiquitin-like modifiers did not change significantly (Fig. 4A).

SUMO1 and SUMO2/3 are known for their roles in the regulation of nuclear protein function^34,35^ (Supplementary Fig. 3B). To elucidate the effects of SUMO2/3 depletion on protein SUMOylation, we performed pulldowns of SUMO2/3-conjugated proteins under denaturing conditions (Supplementary Data 3). Pulldowns were performed using equal protein amounts (Supplementary Fig. 3C, D). We identified increased SUMO2/3-modification of 122 proteins in senescence, 57% of which were previously reported as SUMO targets^36^ (Fig. 4B). Proteins localizing to nuclear condensates such as PML bodies (PML and SP100), nuclear speckles (SON, SRRM1 and SRRM2), as well as topoisomerases TOP1/2 and senescence-associated proteins HMGA2 and LMNA showed increased SUMOylation in senescent cells (Fig. 4B). In line with previous reports^37^, we observed an increased number of PML nuclear bodies in senescent cells (Supplementary Fig. 3E). Additionally, GO term enrichment analysis showed that besides nuclear proteins, cytoplasmic translation components experienced increased SUMOylation (Fig. 4C, D). Thus, in light of the depletion of soluble SUMO2/3 in senescence, the accumulation of SUMO2/3-modified proteins in lysates prepared under denaturing conditions suggests a potential accumulation of SUMO2/3 in the insoluble proteome in senescence, analogous to the events associated with compromised proteostasis in neurodegeneration^36,38^.

### Ubiquitylation through K11- and K27-linkages accumulates during senescence

Although total ubiquitin levels did not significantly change during senescence, strikingly, senescence was accompanied by decreased levels of 19 ubiquitin E3 ligases (Fig. 4E). Only three ubiquitin E3 ligases, YPEL5, TRIM22 and RNF14 showed a significant increase in their levels during senescence (Fig. 4E). We therefore investigated whether abundance of conjugated ubiquitin as well as ubiquitin chain topology is altered during senescence (Supplementary Data 4 and 5). To this end, ubiquitylated proteins from proliferative or senescent cells were enriched using the OtUBD probe, which exhibits high affinity for mono- and polyubiquitylated proteins^39^. Analysis by targeted MS revealed a significant increase in abundance of K11- and K27-linkages (up to 3.0-fold and 2.0-fold, respectively), highlighting their potential roles in proteome remodeling during senescence (Fig. 4F). Importantly, the levels of conjugated ubiquitin increased during senescence (Fig. 4G). Three deubiquitylating enzymes (DUBs) significantly changed in abundance during senescence: USP48 and USP7 showing a decrease and UCHL1 an increase. UCHL1 regulates free ubiquitin^40^. Therefore, increased levels of this DUB could be a compensatory mechanism aimed at increasing the cellular pool of free ubiquitin.

### Analysis of changes in the ubiquitin-modified proteome during senescence

**Fig. 5:**
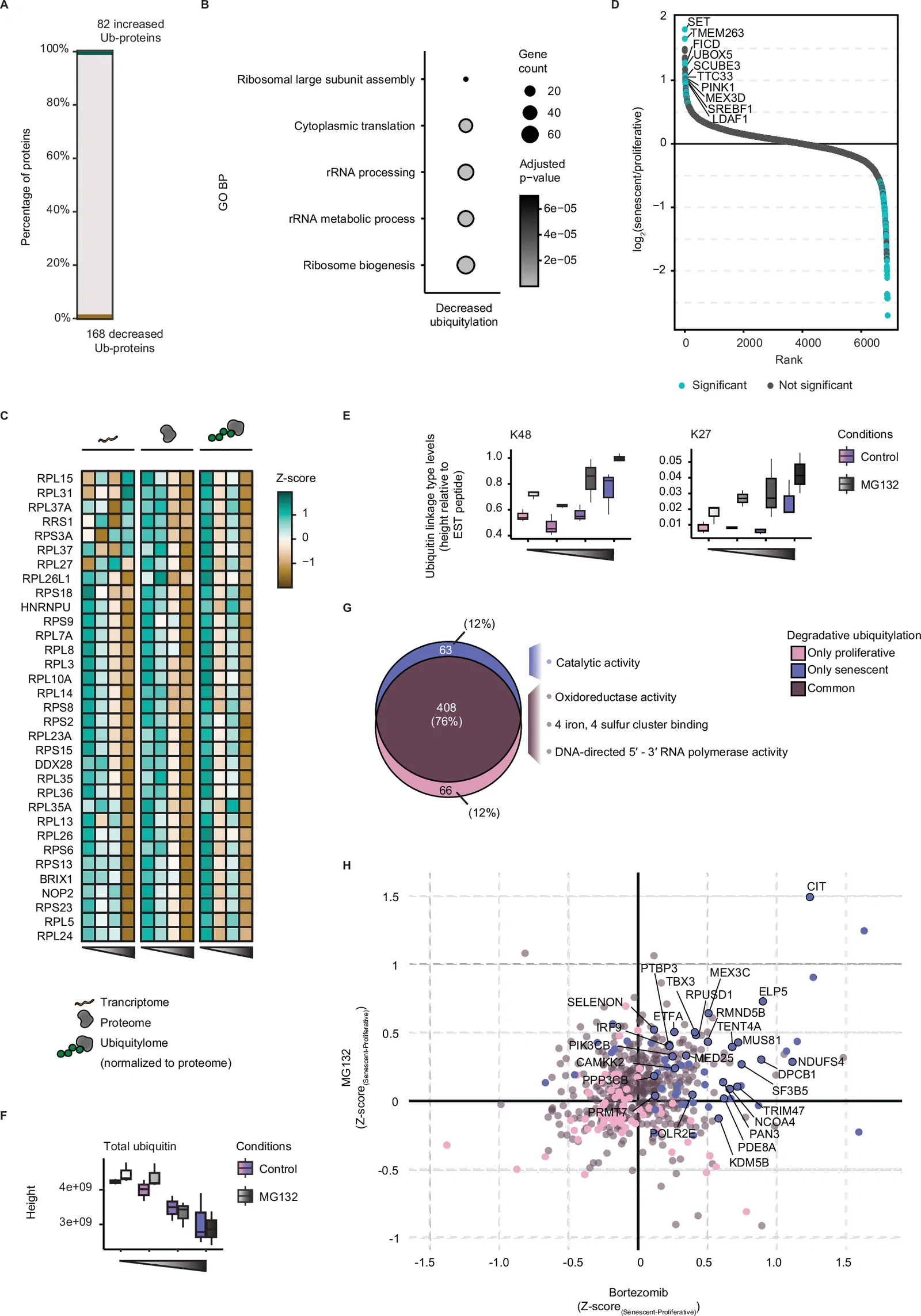
Replicative senescence-induced changes in the ubiquitin-modified proteome. **A** Bar plot indicative of the percentage of proteins with changes in ubiquitylation. Ubiquitylation data is normalized to the proteome, to correct for alterations at the proteome level (GC4, *n*= four age replicates). (**B**) Dot plot showing GO Biological process (BP) terms enriched for proteins with decreased ubiquitylation in senescence with an adjusted *p* value inferior to 0.05 (one-sided hypergeometric test with Benjamini-Hochberg false discovery rate correction). The top five terms are shown. **C** Heatmap showing the Z-score of cytosolic translation factors with decreased ubiquitylation in senescence, and corresponding changes at the transcriptome and proteome level. **D** Rank plot illustrating changes in ubiquitylation levels during senescence, highlighting the top ten proteins with increased ubiquitylation. **E** Box plot displaying the SureQuant^TM^ targeted quantification of K27 and K48 peptides (*n*= three age replicates, seven transitions quantified per age replicate) in control and MG132-treated conditions. Bottom, interior line, and top of the box represent first quartile, median and third quartile, respectively. Whiskers represent minimum and maximum values within 1.5× IQR, with outliers indicated. **F** Box plot displaying the SureQuant^TM^ targeted quantification of the EST peptide (n= three age replicates, seven transitions quantified per age replicate) in control and MG132-treated conditions. Bottom, interior line, and top of the box represent first quartile, median and third quartile, respectively. Whiskers represent minimum and maximum values within 1.5× IQR, with outliers indicated. **G** Area-proportional Euler diagram displaying the overlap of proteins with increased ubiquitylation in proliferative and senescent cells after exposure to 10 μM MG132 or 8 μM of Bortezomib for 4 h, along with enriched GO molecular function terms. **H** Lattice plot showing proteins regulated in proliferative and/or senescent cells upon proteasomal inhibition using MG132 or Bortezomib. Axes denote the Z-score difference between conditions. Colors defined in panel G indicate whether proteins are commonly or uniquely regulated in proliferative and/or senescent states. Text labels highlight proteins associated with the GO molecular function term “catalytic activity”.

By enriching ubiquitylated proteins with the OtUBD probe^39^ under denaturing conditions, we additionally gained insight into the global landscape of ubiquitin-modified proteins in senescence. We identified 250 proteins with significant changes in their ubiquitylation status in senescent cells (Fig. 5A, and Supplementary Data 6). We found that proteins involved in cytoplasmic translation and ribosome biogenesis displayed reduced ubiquitylation even after normalization to their total protein levels, which generally decreased (Fig. 5B, C and Supplementary Fig. 3F). Although significant enrichment for a particular biological process was not found for proteins with increased ubiquitylation in senescence, among the top 10 ubiquitylated proteins were histone chaperone SET, poorly characterized ubiquitin E3 ligases UBOX5 and MEX3D, mitochondrial quality control kinase PINK1 and lipid droplet assembly factor IDAF1 (Fig. 5D).

Subtle changes in the ubiquitin-modified proteome led us to investigate also changes in degradative ubiquitylation by using proteasome inhibitors to prevent degradation of ubiquitylated targets. Treatment of cells with 10 µM MG132 or 8 µM Bortezomib for 4 h before enrichment of ubiquitylated proteins provided insight into the spectrum of putative proteasome substrates in senescence (Supplementary Data 7). Ubiquitin linkage-specific analysis revealed an accumulation of K27- and K48-linkages after inhibition of the proteasome (Fig. 5E and Supplementary Fig. 3G). Interestingly, while levels of K48-linkage increased to a similar level (1.3×) in both proliferative and senescent states, the increase in the K27-linkage after proteasome inhibition was more pronounced in proliferative cells (2.1×) than in senescent cells (1.7×), where linkages of this topology accumulated even in the absence of MG132 (Fig. 5E). Furthermore, while levels of total ubiquitin tended to decrease in senescence, proteasomal inhibition led to an increase of total ubiquitin in age 1 and age 2, but not at age 3 and age 4. (Fig. 5F).

To gain insight into the proteins preferentially targeted for degradation in proliferative and senescent cells, we focused on proteins that showed increased degradative ubiquitylation in response to MG132 and Bortezomib treatments. We observed that 76% of the proteins showed increased degradative ubiquitylation in both conditions (Fig. 5G), with 12% unique to proliferative cells and 12% unique to senescent cells (Fig. 5G). Gene Ontology (GO) enrichment analysis showed that subunits of RNA polymerase I (POLR1A), RNA polymerase II (POLR2A, POLR2E) and RNA polymerase III (POLR3A and POLR3B) were commonly subjected to degradative ubiquitylation in proliferative and senescent cells (Fig. 5G). Among the proteins selectively subjected to degradative ubiquitylation in senescent cells, we found an enrichment of proteins associated with the GO term “catalytic activity” (Fig. 5H). These included proteins associated with transcription (TBX3 and POLR2E) and RNA processing (SF3B5, PTBP3, PAN3, DCP1B, TENT4A and MEX3C). In addition, cell cycle regulators such as CDK1, CCND1, CIT and RMND5B, as well as stress responsive MAPKs (MAPK12, also known as p38γ and the upstream kinase MAP3K6) and signaling regulators (PIK3CB and CAMKK2) showed increased degradative ubiquitylation in senescence.

### Mitochondrial proteins and K27-linked ubiquitylation are associated with the senescence-associated insoluble proteome

**Fig. 6:**
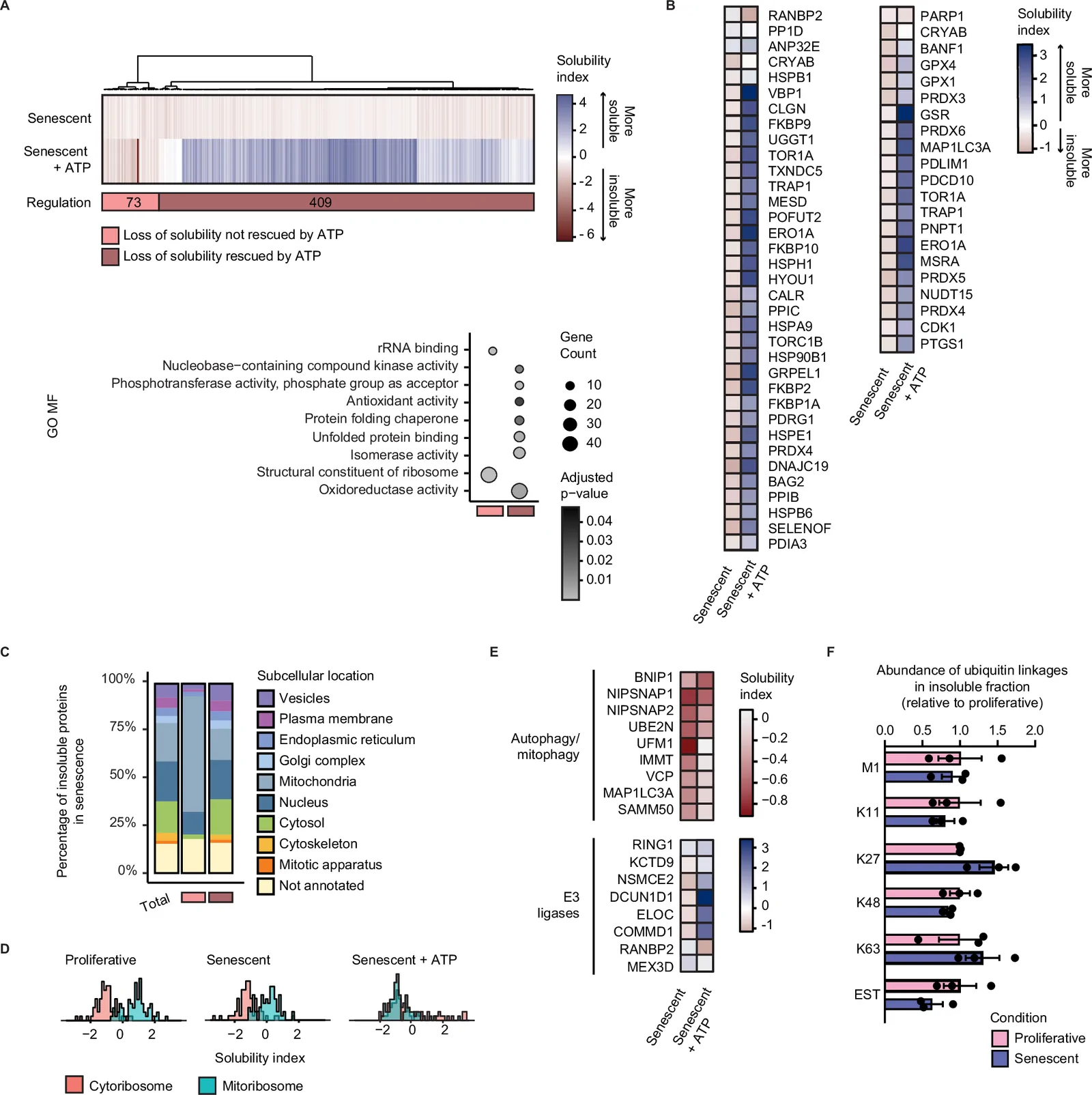
Mitochondrial proteins are enriched in the insoluble proteome of senescent cells. **A** Top: Heatmap comparing the solubility indices of proteins with altered solubility during senescence. The analysis includes early- and late-passage IMR90 cells, defined as proliferative and senescent, respectively. (GC1, *n*= four age replicates). Columns represent protein groups ordered by hierarchical clustering (complete linkage). Bottom: GO molecular function (MF) term analysis for protein clusters defined in the panel above, with a *p* < 0.05 (one-sided hypergeometric test with Benjamini-Hochberg false discovery rate correction). **B** Heatmap for senescent and senescent + ATP conditions, showing solubility index changes in protein folding factors (left) and oxidative stress regulators (right). **C** Stack plot displaying the subcellular distribution of proteins with altered solubility, according to the clusters defined in panel (**A**). **D** Solubility distribution of cytosolic and mitochondrial ribosome proteins in proliferative, senescent and senescent + ATP conditions. **E** Heatmap showing alterations of the solubility index for mitophagy regulators and E3 ligases that lost solubility during senescence. **F** SureQuant^TM^ targeted quantification of ubiquitin linkages in the insoluble fraction of senescent cells. Values normalized to the proliferative condition. Bar plot values represent mean ± SEM (GC1, *n*=three age replicates, each with six transitions quantified per peptide).

Given that senescent cells accumulate protein aggregates^41^, we assessed changes in protein solubility through proteomics, applying a detergent-based separation method to distinguish soluble (solubilized by NP-40) and insoluble (solubilized by SDS) proteins^42,43^. As ATP plays a crucial role in protein solubilization, both as an essential cofactor of molecular chaperones and via a hydrotope-like activity^44^, and considering that we found ATP levels to be depleted in senescent cells (Fig. 3F), we also investigated the effects of ATP replenishment. We identified 550 proteins that became insoluble during senescence, and the majority (74%) were re-solubilized by the addition of ATP (Fig. 6A and Supplementary Data 8). The insoluble fraction contained proteins involved in metabolic regulation, protein folding (such as chaperones and heat shock proteins) and redox homeostasis (Fig. 6B). Interestingly, mitochondrial ribosome subunits became more insoluble during senescence (Supplementary Fig. 4A, B) and remained insoluble even after ATP addition (Fig. 6C, D). Components of the electron transport chain, mitochondrial metabolic enzymes, and mitochondrial protein/import/assembly factors also showed decreased solubility in senescence, possibly reflecting the accumulation of damaged mitochondria during senescence^45^.

Among the members of ubiquitin-dependent degradation pathways, several UPS and autophagy components were enriched in the insoluble proteome fraction (Fig. 6E), such as autophagy/mitophagy regulators, ubiquitin and SUMO E3 ligases, namely RING1, NSMCE2 and RANBP2 (Fig. 6E), as well as proteasome core particle subunits and proteasome activators (Supplementary Fig. 4C). Interestingly, the RNA-binding E3 ligase MEX3D showed both significantly increased non-degradative ubiquitylation and decreased solubility in senescent cells (Figs. 5D, 6E). Analysis of ubiquitin linkages by targeted MS revealed an enrichment of the K27 linkage in the insoluble fraction of senescent cells (Fig. 6F and Supplementary Data 4 and 5). Interestingly, a recent characterization of proteins modified by K27-linked ubiquitin after proteotoxic stress reported an enrichment of molecular chaperones^46^.

### Cellular senescence elicits a response distinct from other stress responses

Following the characterization of proteome changes during replicative senescence, we aimed to understand to what degree replicative senescence resembles other cellular stress responses. To this end, we exposed proliferative IMR90 fibroblasts to an array of perturbations focused on proteostasis by inhibiting the UPS system (Bortezomib and MG132), autophagy (Bafilomycin A1) and protein folding via the HSP90 chaperone (DMAG-17). We also treated cells with hydrogen peroxide to induce oxidative stress or starved them of amino acids to induce reversible cell cycle arrest and autophagy. Furthermore, we included a treatment with a small molecule—Inflachromene (ICM)—that was shown to induce a senescence-like phenotype^47^.

**Fig. 7:**
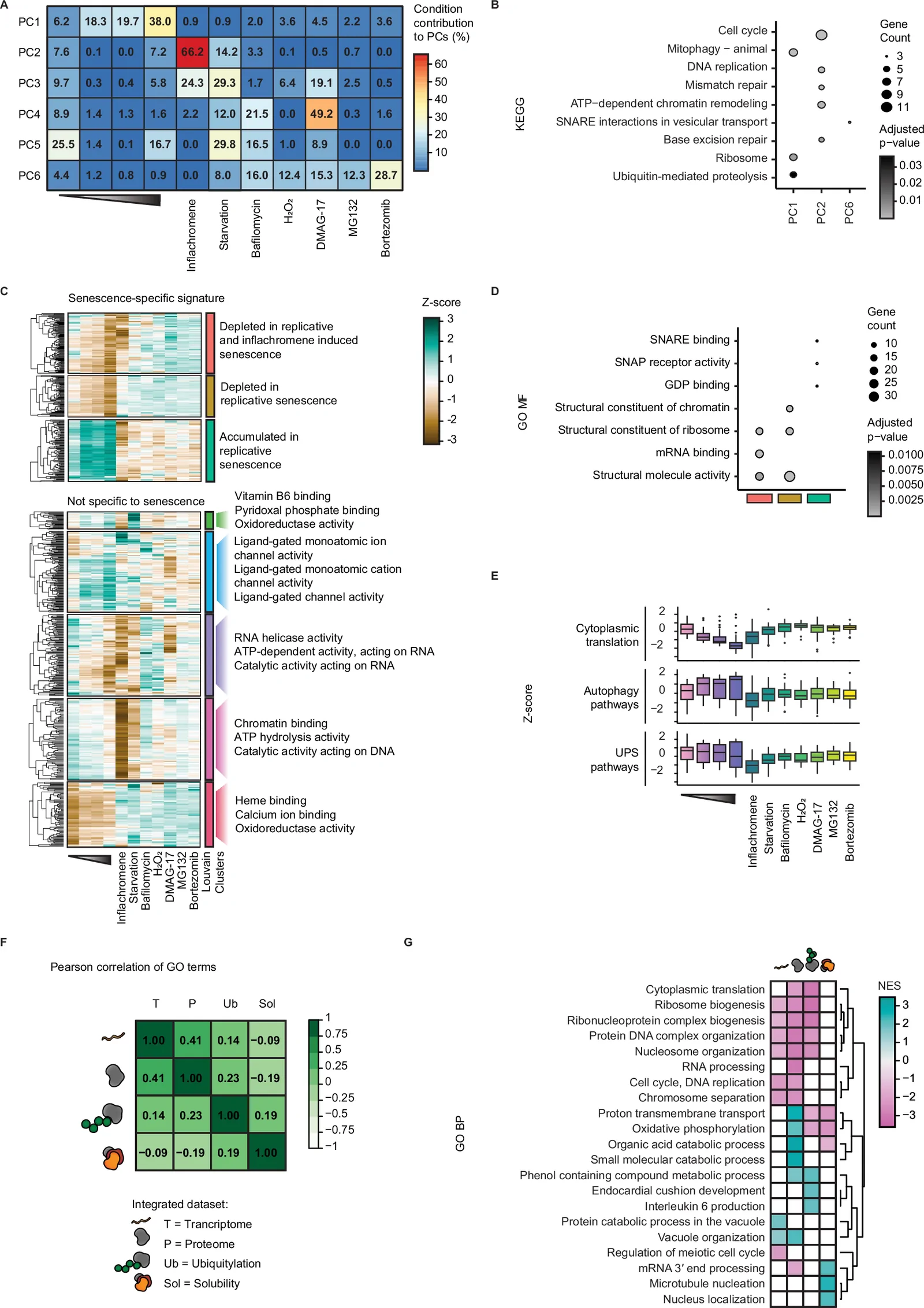
Cellular replicative senescence is a characteristic response not mimicked by other stressors. **A** Heatmap showing the contributions to PCA decomposition of different stress conditions. (GC1, *n*= three age replicates) **B** Dot plot showing KEGG terms enriched for the top 100 contributing proteins to each PC, as defined in panel A. GO terms are considered significant with an adjusted *p* value < 0.05 (one-sided hypergeometric test with Benjamini-Hochberg false discovery rate correction). Only the top three terms are shown. **C** Heatmap showing Z-scores of proteins differentially regulated during senescence, and their corresponding response to other stressors. Protein groups were organized in eight clusters using the Louvain algorithm. For each cluster, protein groups are clustered using hierarchical clustering (complete linkage). Clusters are further separated into senescence-specific and -unspecific. **D** Dot plot of GO molecular function (MF) terms enriched for each senescence-specific Louvain cluster (*p* value < 0.05, one-sided hypergeometric test with Benjamini-Hochberg false discovery rate correction) as defined in panel (**C**). **E** Box plot showing Z-scores of proteins differentially regulated at the proteome level in senescence, and their regulation in response to distinct stressors. Bottom, interior line, and top of the box represent first quartile, median and third quartile, respectively. Whiskers represent minimum and maximum values within 1.5 × IQR, with outliers indicated. **F** Correlation matrix displaying Pearson correlations of GO BP terms between the datasets evaluated during senescence. **G** Heatmap showing normalized enrichment score (NES) values for the GO BP terms with the strongest contributions to the transcriptome, proteome, ubiquitylome, and solubility datasets.

We performed analysis of proteome changes in response to these treatments and used principal component (PC) analysis to understand the similarities and differences considering all significantly regulated proteins during replicative senescence (Supplementary Data 9). This revealed that PC1 variance was predominantly influenced by the IMR90 senescence growth curve (Fig. 7A and Supplementary Fig. 5A). Among the top 100 proteins (based on highest absolute PC scores) from each PC, 91 were uniquely found in PC1, and included cytoplasmic ribosomal proteins and ubiquitin E3 ligases, such as RNF14, NEDD4L and UBE2D3. Interestingly, KEGG enrichment analysis revealed that this group of proteins is enriched in proteostasis network components such as ubiquitin-mediated proteolysis, mitophagy and ribosome (Fig. 7B). This supports the notion that replicative senescence differs from other stress responses and that remodeling of the proteostasis network is likely an important distinctive factor. Induction of the senescence-like phenotype using ICM did not fully recapitulate the changes observed in replicative senescence (Fig. 7A). The ICM-induced effect is better encompassed by PC2, with the top 75 contributing proteins involved in nuclear processes, such as chromatin remodeling, DNA replication and repair and cell cycle regulation (Fig. 7B).

To better understand which proteins were commonly or distinctly regulated in replicative senescence versus other stress conditions, we performed Louvain clustering of the PC scores into 8 distinct clusters. We observed that the levels of 168 proteins were exclusively changing (up or down) in replicative senescence, while levels of an additional 110 proteins decreased in both replicative senescence and ICM-induced senescence (Fig. 7C). Loss of RNA-binding proteins was observed in both replicative senescence and upon ICM treatment, although loss of chromatin-binding proteins was more pronounced in replicative senescence (Fig. 7D and Supplementary Fig. 5B). Interestingly, depletion of ribosomal proteins is only partially mimicked by ICM-treatment, with a specific subset of ribosomal proteins depleted uniquely in replicative senescence (Supplementary Fig. 5C). The increased levels of autophagy proteins were also specific to cellular senescence (Fig. 7D, E and Supplementary Fig. 5C).

Next, we aimed to understand whether the senescence-associated proteome signature was also apparent in changes in mRNAs, protein ubiquitylation or protein solubility. We first assessed the comparability of the datasets by verifying that key senescent biomarkers were expressed at consistent levels across different datasets (Supplementary Fig. 5D). Then, we calculated the normalized enrichment scores (NES) of GO biological process terms, enabling direct comparison of distinct datasets, while maintaining information about the magnitude and direction of enrichment of different processes. A positive Pearson correlation of NES was observed between the proteome and transcriptome (ρ of 0.41), as well as between the proteome and ubiquitylome (ρ of 0.23) (Fig. 7F). Notably, mRNA processing factors were primarily affected on the proteome level, whereas translation and ribosome biogenesis factors exhibited additional changes in ubiquitylation (Fig. 7G). Mitochondrial proteins showed changes across multiple levels, while autophagy-associated proteins were primarily affected at the transcriptome level (Fig. 7G). Together, these findings indicate that remodeling of the senescent proteome results from distinct layers of gene expression regulation that vary according to the specific cellular process involved.

## Discussion

Although cellular senescence was identified more than 60 years ago^48^, the molecular mechanisms that accompany senescence on the proteome level remain largely unclear. Here, we profiled changes in the transcriptome, proteome, SUMO2/3- and ubiquitin-modified proteome, as well as in protein solubility during replicative senescence of human fibroblasts. We found that proteome remodeling occurs in a coordinated manner during senescence, with protein depletion being a prevalent feature. Nuclear and chromatin proteins are particularly affected in senescence, consistent with the notion that changes in the chromatin landscape and loss of nuclear proteins are drivers of cellular senescence^49^. Depletion of some nuclear proteins is considered a marker of cellular senescence, such as of LMNB1^50^, linker and core histones^51,52^. We now show that the loss of nuclear and chromatin-associated proteins is a widespread phenomenon during replicative senescence, and that it occurs in a coordinated manner, with a subset of proteins associated with mRNA processing and DNA replication, as well as linker histones being lost only later during senescence. Alterations in the transcriptome of senescent cells do not fully explain these alterations: for example, proteins involved in the regulation of RNA polymerase II elongation were lost despite a tendency to be transcriptionally upregulated, and this may contribute to the previously observed increase in RNA polymerase II elongation speed in different aging models^53^. Furthermore, similar observations were recently reported in the killifish aging model, where RNA- and DNA-binding proteins were depleted on the proteome but not at the transcriptome level^54^. Moreover, we found that inhibition of the HSP90 chaperone in part mimicked the depletion of RNA-binding proteins observed in senescence. One of the targets of HSP90 is the cytoplasmic capping enzyme, which was shown to be depleted upon HSP90 inhibition and exhibits an interactome rich in RNA-binding proteins^55^.

Our integrative analysis revealed that changes in the transcriptome and loss of nuclear proteins are not the only defining features of cellular senescence. In addition, remodeling of the proteostasis network has a strong and distinctive contribution that is not mimicked by other cellular stressors. Specifically, this includes changes in the translation and ubiquitin-mediated degradation machinery. Both nucleolar ribosomal biogenesis factors and cytosolic ribosomal proteins are depleted, the latter strongly influenced at the post-translational level by ubiquitin and SUMO2/3. Interestingly, cytosolic ribosomal subunits do not accumulate upon inhibition of the proteasome or autophagy and thus do not appear to be degraded through these pathways in senescence but are lost through alternative mechanisms. Additionally, we show that proteins associated with cytoplasmic translation become more SUMOylated during senescence, namely members of the large ribosomal subunit (e.g., RPS8, RPL23A, RPL26, RPL35 and RPL35A), which show decreased ubiquitylation. Although SUMOylation of ribosomes and associated biogenesis factors is essential for proper assembly and maturation of the ribosome complex^56^, persistent SUMOylation of these subunits impedes their export to the cytoplasm and final maturation. This subsequently leads to known features of cellular senescence such as nucleolar hypertrophy and compromised translation^9,57^. Indeed, perturbations of ribosome biogenesis have been shown to drive cells into senescence^58^ through the activation of p53 by the 5S ribonucleoprotein particle; and ribotoxic stress has been shown to be a determining feature of replicative senescence^59^. Moreover, ribosomal collisions during translation were observed to increase in killifish brain^54^, *Caenorhabditis elegans* and *Saccharomyces cerevisiae*^60^ during aging. We demonstrate significant changes in levels of proteins associated with stalled ribosomes: ubiquitin E3 ligase RNF14 as well as GCN1 and ASCC2/3 increase in abundance whereas ZNF598 decreases. These findings suggest a complex regulation of ribosome stalling-associated quality control mechanisms.

Strikingly, mitochondrial ribosomal subunits appear to be regulated in a distinct way and segregate to the insoluble proteome together with specific autophagy receptors and ubiquitin E3 ligases. A previous proteomics study revealed extensive rewiring of the mitochondrial proteome after chemotherapy-induced senescence^61^. Mitochondria are known to accumulate damage^62^, become dysfunctional in senescence and have been described to become insoluble in brains of older mice^63^. Damaged mitochondria are known to be decorated with distinct ubiquitin chains and targeted for degradation by mitophagy. The latter is known to be repressed in cellular senescence^64^, consistent with our observation that despite increased levels of autophagy components, autophagic flux is compromised in senescent cells. In contrast, the levels of the core proteasome particle subunits tended to increase, specifically immunoproteasome subunits, which could be interpreted as a response to cellular stress, given that immunoproteasome and the 20S core proteasome were shown to remove damaged proteins during oxidative stress more efficiently^65,66^. Furthermore, decreased proteasomal activity, and subsequent accumulation of misfolded proteins, have been associated with an increased accumulation of all SUMO paralogs, particularly of SUMO2/3 in insoluble protein inclusions^67^.

Finally, we observed an accumulation of conjugated ubiquitin, predominantly through K11 and K27 linkages, during senescence. Indeed, a recent study reported a decline in the activities of deubiquitylating enzymes such as USP7 and UCHL1 in aged mouse and killifish brain^68^. K11-linked ubiquitylation, mediated by the APC complex, initiates the degradation of cell cycle regulators and damaged ribosomes^69^ and branched K11-K48-linked ubiquitylation can enhance degradation of proteins associated with cell cycle and protein quality control^70,71^. A recent study showed that histones and other chromatin-associated proteins are modified with K27-linked ubiquitin in a process dependent on p97/VCP segregase activity^72^, but not the proteasome.

Together, our findings reveal the intricate remodeling of cellular processes in senescence, which arise not only through the dysfunction of the transcription-translation axis, but also due to alterations of the proteostasis network and a depletion of the chromatin-associated proteome. Based on the comparison to other types of cellular stress, we have identified depletion of ribosomal proteins and remodeling of ubiquitin-mediated degradation as distinctive features of replicative senescence. This study provides a comprehensive analysis of the proteome and its post-translational modifications as well as protein solubility, offering systematic insights into the proteome landscape during replicative senescence. While transcriptomic studies suggest the existence of a core senescence program^73^, its conservation at the proteomic level remains unresolved. Nevertheless, shared features across senescence models have been reported. For instance, loss of histone H1 variants, Lamin B1, and nuclear pore complex components^51,74,75^ has been observed in both replicative senescence and oncogene-induced senescence (OIS). Furthermore, ribosome biogenesis has been shown to be attenuated in both replicative senescence and Rb-dependent senescence^76^, whereas autophagy-mediated degradation of ribosomes has been documented for OIS^77^. In addition, mitochondrial dysfunction^78–80^ and attenuation and/or remodeling of the UPS system have been described consistently across senescent models^81–83^. These findings suggest that a core senescence response may also extend to the proteome. However, similar to the transcriptomic signature, the proteomics response is likely dynamic in nature—shaped by context, timing, and cell-type specific factors—highlighting the need for future studies to identify and define conserved mechanisms across senescence models. Together, these discoveries may open avenues for the development of new therapeutic interventions for the targeting of senescent cells.

## Methods

### IMR90 cell culture

Human embryonic lung fibroblasts (IMR90-83 cells)^12^ were obtained from the Coriell Institute for Medical Research (Camden, NJ, USA) and maintained in Dulbecco’s modified Eagle’s medium (Invitrogen) supplemented with 10% fetal bovine serum (v/v), 1 mM sodium pyruvate (Gibco^TM^, 11360-070, Thermo Fisher Scientific, USA), 1 × MEM non-essential amino acids (Gibco^TM^, 1140-035, Thermo Fisher Scientific, USA) and 1× antibiotic-antimycotic mixture (Gibco^TM,^ 1760-250106, Thermo Fisher Scientific, USA). Cells were maintained at 37 ^**◦**^C in a humidified atmosphere in 5% CO_2_ and sub-cultured at a confluency of 80%^84^. Cell numbers were assessed by manual counting using a Neubauer chamber. Population doubling was tracked according to the following formula: ${Population\; doubling}=\,\log 2\left({collected}\right)-\log 2({seeded})$.

The present study started with the establishment of 3 independent IMR90 growth curves. IMR90-83 cells at a population doubling of 15.2 were used to establish three independent cultures that were maintained for ~3 months until most of the cells underwent senescence. We refer to these as growth curves (GC), numbered from one to three. In all experimental procedures, early-passage IMR90 cells (proliferative, age 1) were defined as having undergone <32 cumulative population doublings, and late-passage cells (senescent, age 4) had undergone >60 cumulative population doublings. For time-course experiments, two intermediate stages were also taken for analysis: age 2 and age 3, with cumulative population doublings of 40 and 50 ± 2, respectively. At each stage, multiple aliquots were frozen for each growth curve. For analysis, one aliquot per growth curve was thawed and immediately split into four cultures, referred here as age replicates. These were processed independently for analysis. No statistical method was used to predetermine the number of GC or age replicates sample size for each analysis.

### Cell treatments

Autophagic flux was assessed for proliferative and senescent cells at 80% confluency. Before lysis, cells were incubated for 2 h in complete or Earle’s balanced salt solution (EBSS) medium in the presence or absence of 2.5 µM Bafilomycin A1 (B110000-1MG, Toronto Research Chemicals, Canada). For the detection of putative proteasome substrates, 80% confluent cells were treated for 4 h with 10 µM MG132 or 8 µM Bortezomib.

For the comparison of replicative senescence with distinct stressors, proliferative cells were exposed to distinct stressors. At a confluency of 80%, the following compounds were added to complete medium: 10 µM Inflachromene (HY-113772, MedChemExpress, USA) for 5 days, 50 nM MG132 (M7449, Sigma-Aldrich, Germany) for 5 days, 2.5 nM Bortezomib (S1013-0005, Selleckchem, USA) for 5 days, or 370 nM DMAG-17 (HY-10389, Hölzel Diagnostika Handels GmbH, Germany) for 3 days. For induction of starvation, cells at 80% confluency were washed with 1 × PBS twice, and the medium was replaced with complete medium with 0.01% FBS for 1 day before lysis. For oxidative stress induction, proliferative cells were treated with 500 µM hydrogen peroxide (H1009, Sigma, Germany) for 2 h, after which all hydrogen peroxide was removed from the medium, and the cells were lysed 24 h later.

### Native and denaturing protein extraction

For protein extraction, cells were washed 2× with cold PBS and scraped off the plates in lysis buffer. For native lysis, modified RIPA buffer (50 mM Tris pH 7.5, 150 mM NaCl, 1 mM EDTA, 1% NP-40, 0.1% sodium deoxycholate) was used, while in denaturing conditions either a urea buffer (8 M urea, 100 mM NaCl, 50 mM TEAB) or complete RIPA (50 mM Tris pH 7.5, 150 mM NaCl, 500 mM EDTA, 1% NP-40, 0.5% sodium deoxycholate, 1% SDS) was used. Lysis solutions were supplemented with protease inhibitors (Complete protease inhibitor cocktail tablets, Roche Diagnostics), phosphatase inhibitors (1 mM sodium orthovanadate, 5 mM β-glycerophosphate, 5 mM sodium fluoride; all from Sigma) and deubiquitylating enzyme inhibitor (10 mM N-Ethylmaleimide, Sigma-Aldrich Chemie GmbH). Lysates were then incubated on ice for at least 15 min. For native and total RIPA lysates, NaCl was added to a final concentration of 500 mM. Protein extracts were then sonicated and cleared by centrifugation at 16,000 × *g* for 20 min at 4 °C.

For the extraction of SUMOylated proteins, cell pellets were lysed using 4% SDS in 50 mM Tris pH 8.0, supplemented with protease and kinase inhibitors at the same concentrations as described above. The concentration of NEM was increased to 20 mM. and NaCl was added to protein lysates to a final concentration of 500 mM, followed by sonication and clearing by centrifugation at 16,000 × *g* for 20 min at 4 °C. After this, protein lysates were diluted 10-fold with full RIPA buffer without SDS.

### Peptide generation using in-gel and in-solution digestion

In-solution protein digestion was used for the quantitative evaluation of the replicative senescence proteome. Briefly, proteins extracted using modified RIPA buffer were first precipitated with 100% acetone at −20 °C overnight. The precipitated proteins were collected by centrifugation, and pellets were resuspended in denaturation buffer (6 M urea, 2 M thiourea, 50 mM Tris, 75 mM NaCl) and incubated in a shaker (1,000 rpm) for at least 30 min at RT. Protein samples were reduced using 2 mM DTT (45 min, 300 rpm) and alkylated using 11 mM chloroacetamide (30 min, in the dark). Then, protein samples were digested using 1:50 of LysC (129-02541, Wako Chemicals, USA; stock concentration of 0.5 μg/μL) for 4 h with shaking at 300 rpm at room temperature (RT). Protein samples were then diluted four-fold with 50 Mm Tris buffer (pH 8.0), followed by a second digestion step using 1:50 of trypsin (0.5 μg/μL) with shaking (300 rpm) at RT overnight. Subsequently, protein samples were purified using C18 Sep-Pak columns with 200 mg sorbent and eluted using 50% acetonitrile (ACN). Peptides were completely dried using vacuum centrifugation at 45 °C and resuspended in 150 mM HEPES (pH 8.5) with 30% ACN.

For other experiments, an in-gel based digestion was used, where proteins were mixed in a 1:1 volume ratio with 1×NuPAGE LDS Sample Buffer (Life Technologies) with 1 mM DTT and heated at 70 °C for 10 min. After cooling down, proteins were alkylated using 5.5 mM chloroacetamide and allowed to incubate for 30 min in the dark at RT. Samples were then loaded onto Bis-Tris NuPAGE gels (Life Technologies) and allowed to run ~1 cm into the resolving gel, followed by fixing using a fixing solution (50% methanol, 10% acetic acid) and staining with a Colloidal Blue staining kit (Life Technologies) following the manufacturer’s instructions. Each lane was then cut into small cubes (~1 mm³) that were subsequently destained (25 mM ammonium bicarbonate, 50% ethanol) and dehydrated (100% ethanol, for 10 min). After dehydration, gel pieces were rehydrated with trypsin solution (T6567, Sigma, Germany; 12.5 μg/mL in 50 mM ammonium bicarbonate), and the digestion reaction was allowed to proceed overnight at 37 °C. After protein trypsinization, peptide extraction buffer (30% ACN, 3% trifluoroacetic acid (TFA)) was added to stop the reaction, and peptides were extracted in three steps using successively increasing concentrations of ACN: peptide extraction buffer solution, buffer B (80% ACN, 0.5% acetic acid) and 100% ACN. Peptide-enriched supernatants were then combined and concentrated using vacuum centrifugation at 45 °C to decrease ACN concentration to <30% (v/v). Samples were then acidified using TFA solution to pH <2 and loaded onto C18 StageTips for desalting before MS injection.

### 16plex TMTpro^TM^ isobaric labeling

Quantitative proteome evaluation was carried out using the TMT-based quantification method^85,86^. Protein samples were subjected to in-solution digestion prior to labeling. Peptides were eluted from Sep-Pak columns in 2 ml 50% ACN and fully dried in a vacuum concentrator at RT. Fully dried peptides were resuspended in 150 mM HEPES (pH 8.5) in 30% anhydrous ACN. Peptide concentrations were determined by absorption at 280 nm, using a Thermo Fisher Scientific Nanodrop 200 spectrophotometer (Thermo Fisher Scientific, USA), and the peptide concentration was adjusted to the sample with the lowest concentration in the set (a minimum of 100 µg of peptides was used). Peptides were mixed with TMTpro^TM^ 16plex Isobaric label Reagent Set label (25 µg/ µL, A44522) in a ratio of (1:5 w/w) and incubated for 1 h at RT while shaking at 500 rpm. Reactions were quenched by adding 5% hydroxylamine to a final concentration of 0.4% and incubated for at least 15 min at RT at 500 rpm. Subsequently, small aliquots of each labeled sample were combined in equal volumes and pre-analyzed using mass spectrometry for labeling efficiency. Based on these results, sample concentration was adjusted for the final experiment to achieve equimolar amounts of TMT-labeled peptides^87^. Peptide samples were pooled at the corrected ratios and purified in Sep-Pak columns, followed by fractionation into 8 fractions via micro-tip based strong cation exchange chromatography (Micro-SCX)^88^, using the following pH fractions: 3, 3.6, 3.9, 4.25, 4.8, 5.5, 7 and 11. Finally, these fractions were desalted using C18 StageTips^88^ before injection into the instrument.

### Enrichment of SUMOylated proteins

Pulldown of SUMO2/3-modified proteins was achieved by conjugating mouse anti-SUMO2/3 antibody (8A2, Abcam ab81371) to Dynabeads^TM^ protein G beads (10004D, Thermo Fisher Scientific, USA). For each pulldown, a total of 5 µg of SUMO2/3 antibody was added to 0.750 mg of Dynabeads according to the manufacturer’s instructions. Subsequently, antibodies were crosslinked to the beads using 5 mM bis(sulfosuccinimidyl)suberate (BS3) Crosslinker (21580, Thermo Fisher Scientific, USA), following the manufacturer’s instructions, for a total of 30 min. The cross-linking reaction was stopped by adding 1 M Tris-HCl buffer pH 7.5 to a final concentration of 50 mM and allowing the reaction to incubate for 15 min with rotation. Then, the cross-linked Dynabeads were washed 3× with PBS + 0.1% Tween-20, before blocking with 0.5% BSA in PBS + 0.1% Tween-20 with rotation for 30 min at RT. After this, the beads were washed 3× with PBS + 0.1% Tween-20, before resuspension in 100 µL of the same buffer. The pulldown of SUMO2/3-modified proteins was carried out by incubating 100 µL of cross-linked Dynabeads with 1 mg of protein lysate. The suspension was incubated overnight at 4 °C with gentle rotation. After this, the supernatant was discarded, and the beads were washed gently 3× with PBS + 0.02% Tween20. Elution of SUMO2/3-modified proteins was carried out by adding 2× NuPAGE^TM^ LDS buffer supplemented with 10 mM of DTT and incubation for 20 min at 70 °C with rotation at 500 r.p.m.

### Enrichment of ubiquitylated proteins

Pulldown of proteins modified by ubiquitin was carried out using the OtUBD probe^39^. The OtUBD probe was conjugated to SulfoLink Coupling Resin (Thermo Fisher Scientific, USA) and then used for pulldown of ubiquitylated proteins in denaturing conditions, using a protocol adapted from Zhang et al., 2022^39^. Briefly, cells were directly lysed in denaturing buffer (8 M urea, 100 mM NaCl, 50 mM TEAB). Protein concentrations were quantified using Bradford, and 1 mg of protein was used for the pulldown. The beads were pre-incubated with 2 bed volumes of elution buffer (100 mM glycine-HCl, pH 2.5) and equilibrated using 20 bed volumes of column buffer (50 mM Tris-HCl, 150 mM NaCl, 1 mM EDTA, 0.5% Triton-X, 10% glycerol, pH 7.5). The protein lysate was diluted 1:1 with native lysis buffer (50 mM Tris-HCl, 300 mM NaCl, 1 mM EDTA, 0.5% Triton-X100, pH 7.5) to reduce urea concentration to 4 M, and samples were incubated overnight, with rotation, at 4 °C with the OtUBD-conjugated beads. The beads were then washed sequentially with column buffer, wash buffer-1 (50 mM Tris-HCl, 150 mM NaCl, 0.05% Tween 20, pH 7.5), wash buffer-2 (50 mM Tris-HCl, 1 M NaCl, pH 7.5), and ultrapure water. Finally, ubiquitylated proteins were eluted from the beads by incubation with SDS buffer (50 mM Tris-HCl, pH 6.8, 2% SDS, 5% glycerol, 100 mM DTT, 0.005% bromophenol blue) with heating at 65 °C, 500 rpm, for at least 30 min. Eluted proteins were loaded onto a polyacrylamide gel and prepared for analysis by the in-gel digestion protocol.

### Solubility assessment

The assessment of protein solubility was carried out using an adapted protocol from Sridharan et al. ^42^. Briefly, cell pellets were resuspended at a ratio of 1:100 (v/v) in a lysis buffer composed of PBS supplemented with deubiquitylase, phosphatase and protease inhibitors, and 1.5 mM MgCl_2_. Cells were lysed through 3 freeze-thaw cycles. Crude lysates were split into 2 equal aliquots, and concentration of the lysates were adjusted to 3.5 µg/µL. One aliquot was incubated with 0.25 u/mL of Benzonase for 30 min at 4 °C with shaking at 500 rpm, followed by addition of 1% SDS (total fraction). To the other aliquot, NP-40 was added to a final concentration of 0.8%, and the insoluble fraction was collected by ultracentrifugation (4 °C, 20 min, 100,000 × *g*). The supernatant was saved as the soluble fraction. The pellet was washed twice with 100 µL of lysis buffer containing 0.8% of NP-40 and solubilized with 100 µL of lysis buffer containing 1% SDS and 0.25 u/mL Benzonase. Finally, equal volumes of all fractions (soluble, insoluble and total) were mixed with 1×LDS buffer supplemented with 1 mM DTT, followed by a 5-min incubation at 90 °C. Equal volumes of these samples were then used for evaluation by mass spectrometry.

### Mass spectrometry acquisition parameters for 16plex TMTpro^TM^-labeled samples

The proteome of IMR90 cells was acquired using data-dependent acquisition (DDA), through TMT-based quantification. Peptide fractions labeled with TMTpro^TM^ 16plex tags were analyzed using the quadrupole Orbitrap mass spectrometer Exploris 480 (Thermo Fisher Scientific, USA), equipped with a UHPLC system (EASY-nLC 1200, Thermo Fisher Scientific, USA) as described^89,90^. Labeled peptides were loaded onto C18 reversed-phase columns (55 cm length packed in-house with ReproSil-Pur 120 C18-AQ 1.9-mm beads, Dr. Maisch GmbH) and eluted with a 120-min linear gradient of ACN (2.4% to 33.6%) containing 0.1% formic acid. Eluted peptides were ionized via electrospray ionization, with the spray voltage being maintained at 1.5 to 2.2 kV, the ion transfer tube temperature set to 250 °C, and source RF lens set to 50%. The 20 most intense peptides, with charges ranging from +2 to +6, were sequentially isolated using the quadrupole within a 0.8 m/z isolation width window. Peptides were then fragmented using a nominal collision energy of 33%, though higher-energy C-trap dissociation (HCD)^91^, within a 10 ppm mass tolerance error. The Orbitrap MS1 scan was performed at R = 60,0000 at m/z = 400, with a scan range of 300–1650 m/z, automatic gain control (AGC) target of 300% and a minimum intensity of 100,000. If a few precursors were found, restrictions would be relaxed. For the Orbitrap MS2 scan, a R = 15,000 at m/z = 100 was used, with an AGC target of 100%, with a maximum injection time of 40 ms.

### Mass spectrometry acquisition parameters for label-free quantification

For all remaining datasets, the data-independent acquisition (DIA) mode was used. Peptides were separated using C18-reverse phase chromatography (in-house packed, 45 cm length, 75 µm inner diameter, ReproSil-Pur 120 C_18_-AQ 1.9-μm silica particles, Dr. Maisch GmbH) on a Vanquish Neo UHPLC system (Thermo Fisher Scientific, USA). Peptide separation and elution were carried out using a 40-min non-linear gradient of 1.6%-32% ACN with 0.1% formic acid, at a nanoflow rate of 300 nL/min. The eluted peptides were subjected to electrospray ionization and analyzed using a Thermo Orbitrap Astral mass spectrometer (Thermo Fisher Scientific, USA). Full MS spectra were acquired in the Orbitrap analyzer in profile mode (scan range: 350 to 1,050 m/z; resolution: 120,000, AGC target value of 300%, maximum injection time: 20 ms). Precursor ions were isolated for fragmentation at a window width of 4 Th over the range of 350 to 1,050 m/z. Fragment ions were recorded in centroid mode in the Astral analyzer via higher energy collision dissociation (HCD: normalized collision energy: 26%, scan range: 150 to 2000 m/z, target value: 5 × 10^4^, maximum injection time: 5 ms, and default charge state: +2).

### Mass spectrometry acquisition parameters for ubiquitin linkage quantification

Quantification of ubiquitin linkages was carried out using the SureQuant^TM^ method of targeted acquisition, following the template provided by Thermo Fisher Scientific, in a Thermo Orbitrap Astral mass spectrometer (Thermo Fisher Scientific, USA). Ubiquitin linkage profiling was done based on nine specific internal standard peptides (Table 1) encompassing all seven lysine-linked ubiquitin modifications (K6, K11, K27, K29, K33, K48 and K63), the methionine-linked linear ubiquitin (M1), and a standard representing total ubiquitin (EST). Unlabeled peptides and isotopically labeled peptides with heavy lysine (U-13C6; U15N) and arginine (U-13C6; U-15N4) were acquired as crude peptides from JPT Peptide Technologies (Berlin, Germany) and used for optimization procedures before implementation of the method. For each peptide, the optimal precursor ion for detection was selected, based on its fragmentation efficiency and quantitative reliability (Table 1). Nominal collision energy (NCE) was individually optimized for each peptide, to ensure maximum sensitivity (Table 1).

**Table 1:** Peptides used for the quantification of ubiquitin linkages

Name of the peptide	Peptide sequence with modifications	m/z	Peptide Charge	Nominal collision energy	Intensity threshold (1% maximum height)
M1 Ub-linkage	^GG^MQIFVK	444.24858	+2	21	4.89 × 10^6^
K6 Ub-linkage	MQIFVK^GG^TLTGK	694.396504	+2	29	8.69 × 10^6^
K11 Ub-linkage	TLTGK^GG^TITLEVEPSDTIENVK	804.0983	+3	23	4.45 × 10^4^
K27 Ub-linkage	TITLEVEPSDTIENVK^GG^AK	703.710352	+3	22	8.16 × 10^5^
K29 Ub-linkage	AK^GG^IQDK	824.471592	+1	28	8.18 × 10^5^
K33 Ub-linkage	IQDK^GG^EGIPPDQQR	824.419859	+3	24	5.33 × 10^5^
K48 Ub-linkage	LIFAGK^GG^QLEDGR	735.900574	+2	28	3.53 × 10^6^
K63 Ub-linkage	TLSDYNIQK^GG^ESTLHLVLR	564.307092	+4	31	2.91 × 10^5^
EST (Total ubiquitin)	ESTLHLVLR	539.318157	+2	29	1.20 × 10^7^

In the peptide sequences, isotopically labeled amino acids (heavy Lys U-13C6; U15N2 and heavy Arg U-13C6; U-15N4) are underscored; ^GG^ represents di-Gly remnants.

For each run, liquid chromatography settings were similar to those specified for the DIA method. Parameters for the MS acquisition were set as follows: spray voltage of 1500 V (-) and 2200 V (+), with the ion transfer tube temperature maintained at 250 °C. Full-scan mass spectra were acquired for heavy and light peptides in a range of 350–1200 m/z, with a maximum injection time of 50 ms, a resolution of 120,000 and a AGC target value of 300%. Heavy peptides matching the provided m/z within 3 ppm, and an intensity threshold set to 1 × 10^10^ of the library peptide, were isolated (isolation window of 1.4 m/z) and subjected to fragmentation (using the optimized NCE) by HCD. MS2 was acquired in scan mode, with spectra acquired within a 150 − 1700 m/z scan range, maximum injection time of 10 ms, resolution of 7500 and AGC target value of 1000%. If at least 3 transitions of the heavy peptide were detected and these exhibited a relative intensity superior to the threshold, fragmentation of the light peptide was triggered and a higher resolution MS2 scan was initiated. MS2 scans covered a scan range of 150–1700 m/z, with a mass offset corresponding to the mass of heavy isotopes normalized to the charge of the peptide. The Orbitrap was used as the detector and was operated with a resolution of 60,000, accumulation time of 116 ms and 10,000% normalized AGC target.

### Analysis of raw MS files

TMT data analysis was performed using the MaxQuant analysis software (v2.0.3.0)^92^, and raw spectra were searched against the reference proteome supplied by UniProtKB/Swiss-Prot (release 2023_01 of 22-Feb-2023, including 569213 sequence entries) using the Andromeda search engine^93^. For each TMTpro^TM^ kit used, TMTpro^TM^ label correction factors were imported into MaxQuant. Additionally, to account for batch effects across multiple TMTpro^TM^ kits, we employed a computational reference-channel normalization approach^94^. Spectra were searched with a mass tolerance of 6 ppm in MS mode, 20 ppm in HCD MS/MS mode, considering strict trypsin and LysC digestion and a maximum of two mis-cleavages. Cysteine carbamidomethylation was allowed as a fixed modification, while N-terminal acetylation and methionine oxidation were considered as variable modifications, with a maximum number of 5 modifications per peptide. TMTPro^TM^ reporters were searched at the MS2 reporter ion level with a mass tolerance 0.003 Da and with PIF R 0.75. Match between runs was allowed, with a matching time window of 0.7 min and an ion mobility window of 0.05. Intensity values were weighted to the reference channel containing all TMTpro^TM^ labels^95^.

DIA raw files were analyzed using DIA-NN v2.02 Academia software^96^. We started by generating a spectral library, using the UniProtKB/Swiss-Prot file from the analysis of the DDA-data as a reference, using the DIA-NN pipeline for library generation. All libraries were generated considering the raw data needed to be analyzed. The DIA raw files were analyzed with the predicted library, allowing for N-terminal M excision, C carbamidomethylation on cysteine and 2 missed cleavages. For the OtUBD pulldown raw files, ubiquitylation was allowed as a variable modification in both the library and the search. The number of variable modifications was set to 5. Oxidation was enabled as a variable modification. Peptide range was set from 7–30; with precursor charges enabled from +1 to +4. A precursor range of 350–1050 m/z was used, as well as a fragment ion range of 150–2000 m/z. Mass tolerance was automatically inferred by DIA-NN. False discovery rates were set to 1% at both protein and peptide levels. For all the datasets analyzed, cross-run normalization was allowed to be RT-dependent, except for the SUMO2/3 pulldown experiment and for the proteome analysis where the same number of proliferative and senescent cells were compared. In these cases, normalization was completely disabled. Finally, SureQuant^TM^ raw files were analyzed using Skyline (64-bit) software version 25.1.0142. Chromatographic co-elution of heavy and light peptides, as well as accurate peak identification, was manually curated. Quantification of peptides was done considering the linear range of detection of the equipment.

### Statistical analysis of proteomics datasets

Output files of MaxQuant, DIA-NN and Skyline software were processed using RStudio version R.4.4.2. The main R scripts used for the analysis are available on GitHub (https://github.com/ndasilva13/Proteomics-data-analysis---Replicative-Senescence.git).

The acquired datasets were first imported into Rstudio (no data were excluded from the analysis) and subjected to data cleaning procedures. For the TMT-labeled dataset, the MaxQuant output was filtered for reverse and potential contaminants, and all empty channels were removed from the analysis. For DIA-NN outputs, filtering and data cleanup was carried out using the *DIAgui* R package^97^. Peptide and protein files were obtained setting a *q* value for precursors and protein groups at 0.01. For protein quantification, the fast MaxLFQ algorithm was applied (from *iq* R package^98^). Both DDA and DIA datasets were then corrected for batch effects using the variance stabilization normalization method^99^. Additionally, ubiquitylated proteins were normalized to the corresponding total protein levels, to account for changes in protein abundance across the four stages. For the solubility experiments, log_2_Intensity levels of insoluble and soluble fractions were first normalized to the total protein levels within each condition, and then the insoluble-to-soluble ratio was calculated using these normalized values and used for the statistical analysis described in the next section. Finally, the solubility index was determined by normalizing each solubility ratio to that observed in the proliferative condition. After this, data quality was inspected for all datasets using the *protti* R package^100^, and correlation between samples was estimated using the *corrplot* R package^101^.

For statistical analysis, only proteins and peptides that were quantified in at least 75% of the technical replicates of each condition were considered. In cases where imputation was needed, k-Nearest Neighbors imputation was performed by condition, using the *impute* R package^102^. Then, differential statistical analysis was performed using linear models implemented in the *limma* R package^103^. We used empirical Bayes moderated *t*-test to identify differences between the different conditions. For the time series experiments, we additionally conducted an empirical Bayes moderated global F-test in the *limma* R package, to assess temporal changes in the dataset. Specifically, for the OtUBD pulldown DIA dataset, linear models were fitted using robust regression, where iteratively reweighed least squared was applied based on Huber’s M-estimation (maximum of 100 iterations) in the *limma* package. *P* values were corrected for multiple testing using the Benjamini-Hochberg method for False Discovery Rate (FDR) control. For each dataset, distinct cut-offs were applied, considering the intrinsic characteristics of the data. For the TMT-based proteome, a protein group was considered significant if between any consecutive age or extreme ages a fold change of ±1.1 and FDR < 0.01 was observed, alongside an ANOVA adjusted *p* value of less than 0.01. For the autophagy flux and solubility experiments, a fold change of ± 1.25 was applied, alongside an FDR of 0.05. For the SUMO2/3 pulldown experiments, an FDR < 0.05 and a fold change of 1.5 were applied. For the OtUBD pulldown, a fold change of ± 1.5 and an FDR < 0.05 were applied; upon treatment with MG132 and Bortezomib, an FDR < 0.05 was applied, and only proteins in the quantile >0.9 were considered significantly regulated. For the cell number-based proteome evaluation, an FDR < 0.01 and a FC > ± 1.25 was applied. For the dataset comprising the proteome and the stressors, cut-offs were only applied to the replicative senescence proteome: a minimal fold change of ± 1.25 for each consecutive age, as well as between the extreme ages, was applied and an FDR < 0.05, alongside a minimal ANOVA *p* value of 0.05.

Finally, for the quantification of the distinct ubiquitin linkages, a report method containing curated information about all quantified transitions was exported from Skyline and imported into RStudio. There, additional filtering was applied to exclude transitions with a mass error of ±3.5 ppm. Based on the chromatogram quality assessment done in Skyline, 7 transitions were then used for the quantification of each peptide (Supplementary Data 4) using peak height as a metric for relative abundance. Each peptide level was normalized to the total amount of ubiquitin in the samples, the latter inferred using the EST peptide, to account for alterations of ubiquitin abundance across ages. Then, differential statistical analysis was carried out using the *limma* R package^103^, applying a moderated *t* test to identify differences between the consecutive ages, as well as between the extremes, in the time series analysis. All tests incorporated empirical Bayes variance moderation, and *p* values were corrected for multiple testing using the Holm method. Data visualization was done using the *ggplot*^104^ and *pheatmap*^105^ R packages. Venn diagrams and overlap maps were generated using the *VennDiagram*^106^ and *UpSetR*^107^ packages, respectively. Lattice plots were generated using the *lattice* R package^108^.

### Clustering analysis

For the proteome and transcriptome datasets, significantly altered proteins and transcripts were subjected to clustering analysis to evaluate changes during senescence progression. The data was pre-analyzed using *NbClust*^109^ and *factoextra*^110^ for the determination and visualization of the optimal number of clusters. Subsequently, the data was clustered using the K-means clustering algorithm, as implemented in the *stats* R package^111^. Briefly, we started by performing different cluster validation methods, such as the Elbow, Silhouette and Gap methods. Although the primary silhouette maximum occurred at k = 2, this would have led to the loss of subtle temporal patterns. To overcome this, we selected the second-highest validation score (k = 6), followed by a manual merging of apparently redundant clusters. This iterative approach yielded 4 distinct clusters that represented the underlying biological features of the data more clearly.

For the integration of the replicative senescence data with other stressors, the entire dataset was Z-scored within each age, using the *base* R package^111^. To cluster the Z-score data presented in Fig. 7, principal component analysis was performed. For downstream clustering steps, only the top 6 PCs explaining at least 90% of the variance were used to build a k-nearest neighbor graph using the *FNN* R package^112^ (v1.1.3.1; k = 15). The Louvain community detection algorithm was applied to identify protein clusters using the *igraph*^113^ R package v1.3.5. All parameters were left to default, unless otherwise mentioned.

### GO term enrichment and reference datasets

For comprehensive data interpretation, GO term enrichment analysis was carried out using the *clusterProfiler*^114^ and *ReactomePA*^115^ R packages. The proteomics datasets were also cross-referenced with distinct reference databases and resources, including the Interphase chromatin binding probability index acquired from Kustatscher *et al*., 2014^15^, Transcription factor annotation retrieved from TFLink annotation^16^, the SUMO annotation acquired from Hendriks and Vertegaal^116^, the Human Protein Atlas for protein localization^13^ (https://www.proteinatlas.org/, downloaded on February 2024), the proteostasis consortium annotation acquired from the proteostasis consortium (https://www.proteostasisconsortium.com/pn-annotation/)^21,22^ (accessed in February 2024) and the CORUM protein annotation of mammalian protein complexes^117^ (downloaded in May 2024).

### Data integration

For the GSEA analysis, log_2_-fold changes between proliferative and senescent cells were used as a basis, except for the solubility dataset, where the solubility ratio for senescent cells was used. GSEA analysis was carried out using the *fgsea* R package^118^, based on the GO biological process terms (total of 7583 gene sets), using a minSize of 15 and maxSize of 500. For each term, normalized enrichment scores (NES) were calculated, by normalizing enrichment scores to the size of each gene set^119^. For each dataset, a pre-filtering step was applied, in which only terms with |NES | > 1.5 and an adjusted p-value of <0.05 were considered. Then, all significant terms for all datasets were arranged in a matrix, and missing values were imputed as zero. Pearson correlation coefficients for all enriched GO terms in the dataset were calculated.

### RNA extraction and sequencing

RNA was extracted using the Qiagen RNeasy Plus Mini Kit (QIAGEN, Netherlands), following the manufacturer’s instructions. Residual DNA was removed using the gDNA eliminator columns from the kit. RNA concentration and quality was further evaluated using A280/260 absorbance (Nanodrop) and the Agilent RNA 6000 Nano Kit (using manufacturer’s instructions, using the Bioanalyser). NGS libraries were prepared using Illumina’s Stranded mRNA Prep Ligation Kit following the Stranded mRNA Prep Ligation Reference Guide (April 2021) (Document 1000000124518 v02). Libraries were prepared with a starting amount of 500 ng and amplified in 10 PCR cycles. Two post-PCR purification steps were performed to exclude residual primer and adapter dimers. Libraries were profiled with a DNA 1000 chip on a 2100 Bioanalyzer (Agilent technologies) and quantified using the Qubit 1x dsDNA HS Assay Kit, in a Qubit 4.0 Fluorometer (Invitrogen by Thermo Fisher Scientific, USA). All 12 samples were pooled in equimolar ratio and sequenced on 1 NextSeq2000 P2 (100cycles) FC, SR for 1 × 116 cycles plus 2 × 10 cycles for the dual index read and 1 dark cycle upfront R1.

### RNA-seq analysis

Reads were quality-controlled using FastQC (v0.11.9) (https://www.bioinformatics.babraham.ac.uk/projects/fastqc/). No data was excluded from the analysis and no statistical method was used to predetermine sample size. Reads were mapped to the reference genome (GRCh38; release 84) using STAR^120^ (v2.7.3a) with default parameters (except --outFilterMismatchNoverLmax 0.04 and—outFilterMismatchNmax 999). Using only the uniquely mapped reads, gene level read summarization was performed using subread featureCounts^121^ (v.2.0.0) with the canonical annotation (GRCh38; v98).

The pairwise differential expression analysis of the age groups was performed in R (v4.1.1) with the *DESeq2*^122^ (v.1.33.5) R package. The genes were subjected to pairwise differential expression analysis, comparing consecutive and extreme ages of the growth curve, and a fold change ±1.1 and an FDR < 0.01 were established as cut-off for significant regulation. Additionally, a likelihood ratio test was performed using the DESeq2 (v1.44.0) package to identify genes that are differentially expressed across all age groups, using an adjusted *p* value of 0.01 as a cut-off for statistical significance.

### Immunofluorescence microscopy

IMR90 cells were grown in 96-well PhenoPlate flat-bottom black plates (Revvity, USA) to 80% confluency. Cells were then fixed with 4% paraformaldehyde for 20 min and permeabilized using 0.5% NP-40 at room temperature for 10 min. Unspecific epitopes were blocked using 3% bovine serum albumin in PBS-Tween20 for at least one h, followed by incubation overnight at 4 °C with mouse anti-PML antibody PG-M3 (1:500, Santa Cruz Biotechnology, sc-966) diluted in blocking solution. Subsequently, cells were incubated with goat anti-mouse Alexa Fluor™ 568-coupled adsorbed secondary antibody (1:500, Life Technologies, A-11004, USA) for 1 h at room temperature. Cell nuclei were stained with 1 mg/mL Hoechst 33342.

Cells were imaged by fluorescence microscopy, using the Opera Phenix High-Content screening microscope (Revvity), in confocal mode. Images were acquired using a 40x/NA 1.1 water immersion objective. The Alexa fluor 568 fluorophore was excited using a 561 nm laser, using an exposure time of 700 ms (90% laser power), with the collected emission ranging from 570 to 630 nm. Nuclear counterstain (Hoechst) was visualized using 405 nm excitation with an exposure time of 100 ms (40% laser power), with an emission wavelength ranging from 435 to 480 nm. To capture the full volume of the cells, a 5-plane Z-stack was acquired using a step size of 0.5 µm (0.0–2.0 µm), for each 7 fields of view per well. Image analysis was performed with the Harmony High-Content Imaging and Analysis Software (version 5.1, Revvity). No data was excluded from the analysis, and no statistical method was used to predetermine sample size. Nuclei segmentation was based on the Hoechst 33342 signal (algorithm C, common threshold 0.70, area >100 µm^2^, splitting coefficient 3.7, individual threshold 0.40, contrast >0.10), and cells at the edges of images were excluded from the analysis. Mean intensity values were extracted for maximum intensity projections for each measurement, and spot detection was performed using algorithm D (detection sensitivity 0.19, splitting sensitivity 0.178, background correction 0.5).

### SDS-PAGE

Protein extracts were separated on 4–12% gradient SDS-PAGE gels (NuPAGE Bis-Tris Precast Gels, Life Technologies) and subsequently transferred onto nitrocellulose membranes (0.45 μm, Amersham™ Protran^®^, USA). The membranes were blocked using 5% (w/w) bovine serum albumin solution in PBS, supplemented with 0.1% Tween-20 for at least 30 min at RT. Subsequently, the membranes were incubated with primary antibodies overnight at 4 °C on a shaking platform. The following primary antibodies were used at a dilution of 1:1,000: mouse anti-PolII CTD4H8 (Santa Cruz Bioctechnology sc-47701), rabbit anti-XRCC1 (Cell Signaling #2735), rabbit anti-SMC2 (Cell Signaling #5329), rabbit anti-LC3B (Sigma, L7543), rabbit anti-GAPDH D16H11 (Cell Signaling #5174), rabbit anti-PSMB8/LMP1 (Cell Signaling #13635), rabbit anti-PSMB10 (Cell Signaling #78385), rabbit anti-PSMC3/TPB1 (Cell Signaling #13923), mouse Anti-MRPL18 (Abcam ab67844), rabbit anti-MRPL50 (Proteintech 17213-AP), rabbit anti-VCP (Cell Signaling #2649S). For immunodetection, secondary antibodies coupled to horseradish peroxidase (Jackson ImmunoResearch Laboratories, UK) were used. Finally, proteins were visualized on a ChemiDoc^TM^ MP imaging system (BioRad, California, USA) by chemiluminescence, using the Immobilon ECL UltraPlus Western HRP Substrate solution (Merck, Darmstadt, Germany) following the manufacturer’s instructions. Signals were quantified by densitometric analysis using the Image Lab software v6.1.0 build 7 (BioRad, California, USA). Total protein content, estimated using Ponceau S staining, was used for normalization of protein levels. Differences in protein abundance between conditions were assessed using unpaired *t* test with Welch’s correction, as implemented in the GraphPad Prism (v.10.4.1) software.

### Proteasome activity measurements

Chymotrypsin-like, trypsin-like and caspase-like activities were measured using the Cell-Based Proteasome-Glo^TM^ assay (G1180, Promega, USA), following the manufacturer’s instructions. Briefly, proliferative and senescent cells were cultured to 80% confluency. Then, cells were trypsinized and subsequently pelleted by centrifugation. To remove residual medium, cell pellets were washed 2× in PBS, and cell numbers were estimated using a Neubauer chamber. Then, a total of 50,000 cells per well in PBS were added to a 96-well PhenoPlate flat-bottom black plate (Revvity, USA). Subsequently, cells were either left untreated or treated with 5 µM FCCP (SML2959-1ML, Sigma Aldrich, Germany) or 10 µM MG132. Treatments were at 37 °C for 20 min before the Proteasome-Glo^TM^ Cell-Based Reagent was added to the cells according to the manufacturer’s instructions. The microplate was incubated at RT for at least 10 min before the luminescence of each sample was measured using a Tecan infinite 200Pro microplate reader, equipped with Tecan I control v2.0.10.0 software. Statistical analysis was carried out using GraphPad Prism (v10.4.1), using the multiple unpaired *t* test with Welch’s correction to account for unequal variances. No data was excluded from the analysis and no statistical method was used to predetermine sample size

### Measurements of ATP levels

ATP levels were quantified using the Luminescence ATP Detection Assay Kit (ab113849, Abcam, Cambridge, UK), according to the manufacturer’s instructions. Briefly, proliferative and senescent cells grew to 80% confluency. Then, 50,000 cells per well in PBS were added to a 96-well PhenoPlate flat-bottom black plate (Revvity, SA), and either left untreated or treated with 5 µM FCCP for a total of 20 min at 37 °C. Cells were subsequently lysed through the addition of the ATP-substrate solution, which simultaneously extracts and stabilizes ATP in solution. The microplate was then incubated for at least 10 min at RT, and the luminescence signal was measured using a Tecan infinite 200Pro microplate reader equipped with Tecan I control v2.0.10.0 software. Statistical analysis was carried out using GraphPad Prism (v10.04.1), using the multiple unpaired *t* test with Welch’s correction, to assess statistical differences between experimental conditions. No data was excluded from the analysis and no statistical method was used to predetermine sample size

### Reporting summary

Further information on research design is available in the Nature Portfolio Reporting Summary linked to this article.

## Supplementary information

Supplementary information

Description of Additional Supplementary Files

Supplementary Dataset 1

Supplementary Dataset 2

Supplementary Dataset 3

Supplementary Dataset 4

Supplementary Dataset 5

Supplementary Dataset 6

Supplementary Dataset 7

Supplementary Dataset 8

Supplementary Dataset 9

Reporting Summary

Transparent Peer Review file

## Source data

Source data

## Data Availability

The RNA-seq data are available in the ArrayExpress^123^ database, under accession code E-MTAB-15914. Imaging data are available on the OMERO web server (https://omero.imb.uni-mainz.de/pub/da-silva-fernandes_et_al_2026). MS proteomics data have been deposited to the ProteomeXchange Consortium via the PRIDE^124^ partner repository. The data from IM90 growth curves proteomes is available under accession code PXD067595, while the proteomes of proliferative and senescent cells (when accounting for equal cell numbers) is available under accession code PXD077797. The protein SUMOylation data during replicative senescence is available under accession code PXD077854. The protein ubiquitylation data during replicative senescence (untreated and proteasome inhibition) is available under accession codes PXD067585 and PXD077923. The ubiquitin linkages data (in total and insoluble fractions of proteins) is available under accession code PXD069085. The protein solubility data during replicative senescence is available under accession code PXD069123, while the data associated with the exposure of IMR90 proliferative cells to different stressors are available under accession code PXD069129. Source data are provided with this paper.

## Code availability

The main R scripts used for the analysis here presented are available on GitHub (https://github.com/ndasilva13/Proteomics-data-analysis---Replicative-Senescence.git).
