# Supplementary material for: Quantitative proteomics reveals coordinated changes in the proteome during replicative senescence
Source: Nat Commun. 2026 Sep 16;17:9877. doi: 10.1038/s41467-026-77686-8 (PMC13582980; doi:10.1038/s41467-026-77686-8)
Supplement: Supplementary file 1 [file 41467_2026_77686_MOESM12_ESM.pdf]

nature portfolio

Corresponding author(s): Helle D. Ulrich
Petra Beli

Last updated by author(s): 16.07.2026

Reporting Summary

Nature Portfolio wishes to improve the reproducibility of the work that we publish. This form provides structure for consistency and transparency in reporting. For further information on Nature Portfolio policies, see our Editorial Policies and the Editorial Policy Checklist.

Statistics

For all statistical analyses, confirm that the following items are present in the figure legend, table legend, main text, or Methods section.

n/a	Confirmed	
☐	☒	The exact sample size (*n*) for each experimental group/condition, given as a discrete number and unit of measurement
☐	☒	A statement on whether measurements were taken from distinct samples or whether the same sample was measured repeatedly
☐	☒	The statistical test(s) used AND whether they are one- or two-sided<br>*Only common tests should be described solely by name; describe more complex techniques in the Methods section.*
☒	☐	A description of all covariates tested
☐	☒	A description of any assumptions or corrections, such as tests of normality and adjustment for multiple comparisons
☐	☒	A full description of the statistical parameters including central tendency (e.g. means) or other basic estimates (e.g. regression coefficient) AND variation (e.g. standard deviation) or associated estimates of uncertainty (e.g. confidence intervals)
☐	☒	For null hypothesis testing, the test statistic (e.g. F, t, r) with confidence intervals, effect sizes, degrees of freedom and P value noted<br>*Give P values as exact values whenever suitable.*
☒	☐	For Bayesian analysis, information on the choice of priors and Markov chain Monte Carlo settings
☒	☐	For hierarchical and complex designs, identification of the appropriate level for tests and full reporting of outcomes
☐	☒	Estimates of effect sizes (e.g. Cohen's *d*, Pearson's *r*), indicating how they were calculated

Our web collection on statistics for biologists contains articles on many of the points above.

Software and code

Policy information about availability of computer code

Data collection	Tools used for mass spectometry acquisition: Xcalibur (v4.7)<br>Tools used for luminiscence acquisition: Biorad Image Lab (v6.1.0 build 7) and Tecan I control (v2.0.10.0).<br>Tools for microscopy data acquisition: Harmony High-Content Imaging and Analysis Software (v5.1)
Data analysis	Tools used in the analysis of mass spectometry data: MaxQuant (v2.0.3.0), DIA-NN (v2.02) Academia software and Skyline 64-bit (v25.1.0142).<br>Tools for statistical analysis: RStudio (version R.4.4.2) and GraphPad Prism (v.10.4.1).<br>Tools used in the analysis of microscopy images: Harmony High-Content Imaging and Analysis Software (version 5.1).<br>Tools used for densitometric analysis: Biorad Image Lab software (v6.1.0 build 7)<br>All the parameters used are described in the "Methods" section.

For manuscripts utilizing custom algorithms or software that are central to the research but not yet described in published literature, software must be made available to editors and reviewers. We strongly encourage code deposition in a community repository (e.g. GitHub). See the Nature Portfolio guidelines for submitting code & software for further information.

Data

Policy information about availability of data

All manuscripts must include a data availability statement. This statement should provide the following information, where applicable:

- Accession codes, unique identifiers, or web links for publicly available datasets
- A description of any restrictions on data availability
- For clinical datasets or third party data, please ensure that the statement adheres to our policy

The transcriptome generated in this study has been deposited in the ArrayExpress database, under the accession ID E-MTAB-15914.
The proteomics datasets generated in this study have been deposited in PRIDE repository, under the following accession IDs:
- Proteome of IMR90 growth curves - PXD067595
- Proteome of proliferative and senescent cells (equal cell number) - PXD077797
- Alterations on protein SUMOylation - PXD077854
- Alteration on protein ubiquitylation, in basal conditions and upon proteasomal inhibition - PXD067585, PXD077923
- Remodeling of ubiquitin linkages in senescence, in total and insoluble fractions - PXD069085
-Alterations on protein solubility - PXD069123
- Exposure of IMR90 proliferative cells to different stressors and autophagy flux experiment - PXD069129
The microscopy data has been deposited in OMERO web server: https://omero.imb.uni-mainz.de/pub/da-silva-fernandes_et_al_2026

Research involving human participants, their data, or biological material

Policy information about studies with human participants or human data. See also policy information about sex, gender (identity/presentation), and sexual orientation and race, ethnicity and racism.

Reporting on sex and gender	IMR90 cell line derives from a female 16 week old fetus.
Reporting on race, ethnicity, or other socially relevant groupings	n/a
Population characteristics	n/a
Recruitment	n/a
Ethics oversight	n/a

Note that full information on the approval of the study protocol must also be provided in the manuscript.

Field-specific reporting

Please select the one below that is the best fit for your research. If you are not sure, read the appropriate sections before making your selection.

☒ Life sciences ☐ Behavioural & social sciences ☐ Ecological, evolutionary & environmental sciences

For a reference copy of the document with all sections, see nature.com/documents/nr-reporting-summary-flat.pdf

Life sciences study design

All studies must disclose on these points even when the disclosure is negative.

Sample size	No statistical methods were used to pre-determine sample size.
Data exclusions	No data were excluded from the analysis.
Replication	For each experiment, a minimal number of 3 independent age replicates were used. Detailed information can be found in the "Methods" section of the paper.
Randomization	Samples were randomized for proteomics data acquisition and luminescence detection assay.
Blinding	n/a

Reporting for specific materials, systems and methods

We require information from authors about some types of materials, experimental systems and methods used in many studies. Here, indicate whether each material, system or method listed is relevant to your study. If you are not sure if a list item applies to your research, read the appropriate section before selecting a response.

Materials & experimental systems

n/a	Involved in the study
☐	☒ Antibodies
☐	☒ Eukaryotic cell lines
☒	☐ Palaeontology and archaeology
☐	☒ Animals and other organisms
☒	☐ Clinical data
☒	☐ Dual use research of concern
☒	☐ Plants

Methods

n/a	Involved in the study
☒	☐ ChIP-seq
☒	☐ Flow cytometry
☒	☐ MRI-based neuroimaging

Antibodies

Antibodies used

- mouse anti-PML (PG-M3) (1:500, Santa Cruz Biotechnology, sc-966)
- mouse anti-PolII CTD4H8 (1:1000, Santa Cruz Bioctechnology sc-47701)
- rabbit anti-XRCC1 (1:1,000, Cell Signaling #2735)
- rabbit anti-SMC2 (1:1,000, Cell Signaling #5329)
- rabbit anti-LC3B (1:1,000, Sigma, L7543)
- rabbit anti-GAPDH D16H11 (1:1,000, Cell Signaling #5174)
- rabbit anti-PSMB8/LMP1 (1:1,000, Cell Signaling #13635)
- rabbit anti-PSMB10 (1:1000, Cell Signaling #78385)
- rabbit anti-PSMC3/TPB1 (1:1,000, Cell Signaling #13923)
- mouse Anti-MRPL18 (1:1000, Abcam ab67844)
- rabbit anti-MRPL50 (1:1000, Proteintech 17213-AP)
- rabbit anti-VCP (1:1,000 Cell Signaling #2649S)
- goat anti-mouse Alexa Fluor™ 568-coupled adsorbed secondary antibody (1:500, Life Technologies, A-11004, USA)

Validation

The antibodies used in this study were acquired commercially.

Eukaryotic cell lines

Policy information about cell lines and Sex and Gender in Research

Cell line source(s)

Human embryonic lung fibroblasts (IMR90 cells) were obtained from the Coriell Institute for Medical Research (Camden, NJ, USA).

Authentication

The cell line exhibited typical morphological characteristics when compared to images and information available on Coriell Institute for Medical Research. No further authentication was done.

Mycoplasma contamination

IMR90 cells were tested regularly for mycoplasm contamination and have tested negative.

Commonly misidentified lines
(See ICLAC register)

n/a

Animals and other research organisms

Policy information about studies involving animals; ARRIVE guidelines recommended for reporting animal research, and Sex and Gender in Research

Laboratory animals

n/a

Wild animals

n/a

Reporting on sex

n/a

Field-collected samples

n/a

Ethics oversight

n/a

Note that full information on the approval of the study protocol must also be provided in the manuscript.

Plants

Seed stocks	n/a
Novel plant genotypes	n/a
Authentication	n/a
