# Supplementary material for: Quantitative proteomics reveals coordinated changes in the proteome during replicative senescence
Source: Nat Commun. 2026 Sep 16;17:9877. doi: 10.1038/s41467-026-77686-8 (PMC13582980; doi:10.1038/s41467-026-77686-8)
Supplement: Supplementary file 2 [file 41467_2026_77686_MOESM13_ESM.pdf]

nature portfolio

Peer Review File

Quantitative proteomics reveals coordinated changes in the proteome during replicative senescence

Corresponding Author: Professor Petra Beli

This file contains all reviewer reports in order by version, followed by all author rebuttals in order by version.

Version 0:

Reviewer comments:

Reviewer #1

(Remarks to the Author)
Cellular senescence is characterized by various changes that impair functions of multiple mechanisms and result in cell enlargement and eventually death. With aging, the removal efficiency of senescent cells deteriorates, and in some cases underlie an aging-associated increase in the incidence of different types of cancer and other disorders. Accordingly, enhancing the removal of senescent cells slows organismal aging and promotes healthspan (Yosef et al., Nat Commun. 2016 Apr 6;7:11190).
In this study, the authors used cultured human lung fibroblasts (IMR90) and mass spectrometry techniques, to characterize the proteomic, ubiquitin modifications and protein solubility signatures of senescent cells.
In a nutshell they discovered that during senescence, cells exhibit modified levels of proteins that reside in different cellular localizations, however, nuclear proteins were overrepresented among these which showed reduced levels. These include proteins that are linked with DNA structure, transcription and RNA processing. Functional clustering showed that proteins that are associated with protein degradation mechanisms, namely the ubiquitin-proteasome system (UPS) and autophagy, show modified levels. While UPS-linked proteins, and especially E3 ubiquitin ligases, showed reduced levels, those which are involved in autophagy exhibited increased levels. In agreement, UPS activity showed reduced levels in senescent cells compared to proliferative cells. Using biochemical techniques and mass spectrometry they found that more proteins are present in insoluble state in senescent cells than in proliferative cells. Among these, mitochondrial and K27-ubiquitinated proteins were over-represented. Finally, they stressed the cells using various compounds that inhibit the UPS, autophagy, HSP90 or induce oxidative stress, and found that senescence elicits proteome modifications that are distinct from the tested stress responses.
These findings are similar to the recent analysis of aging-associated proteomic changes that were characterized in the Killifish brain (Di Fraia et al., 2025, reference #43).
This study provides a “proteomic atlas” of senescent IMR90 cells, that points at specific cellular mechanisms to be majorly affected by senescence. Thus, it could be an interesting and important source for researchers in the field. Yet, several issues should be addressed to enhance rigor and enhance the manuscript.
1. To inhibit proteasome activity the authors used MG132 (Fig. 5). While this is a widely used compound, MG132 also inhibit calpains. To better establish their conclusions and enhance the confidence that the observed proteomic changes stem from UPS inhibition, they should repeat this analysis using an additional, irreversible proteasome inhibitor such as Epoxomicin or Bortezomib (as they did to stress the cells – Fig. 7).
2. One of the interesting conundrums that the authors examined is how senescence modulates post translational modifications (PTMs). Their results show that ubiquitination- and SUMOylation-associated proteins (but not UFMylation) are most prominently affected by senescence. Specifically, SUMO1 and SUMO2 show senescence-associated reduction which is similar to these of E3 ubiquitin ligases (Fig. 4a). Given that SUMOylation has been shown to regulate aging and proteostasis (Princz et al., Sci Rep. 2020 Sep 23;10(1):15513 and Moll et al., Elife. 2018 Nov 7;7:e38635) it is important that the authors follow this interesting direction and explore which proteins exhibit modulated SUMOylation levels in senescent cells. It is also advised that they will discuss the roles of SUMOylation in the discussion.
3. An additional important aspect that could enhance the study is a comparison of proteomic changes in senescent cells and additional organisms (in addition to the Killifish). This can be done by comparing the proteomic changes to these which were observed in the context of aging in the nematode C. elegans. One relevant comparison is of the list of senescent cells-specific insoluble proteins (Fig. 6) with protein that were found by the Kenyon lab to sediment in aged nematodes (David et al., PLoS Biol. 2010 Aug 10;8(8):e1000450). Another comparison should be done between the differentially Ubiquitinated proteins (Fig. 6) and the proteins that were found by the Vilchez lab to exhibit modulated ubiquitination levels in elder worms

Koyuncu et al., Nature. 2021 Jul 28;596(7871):285–290). Such comparison will address the question of how general the reported findings are.

(Remarks on code availability)
I do not have the skills to review computational codes.

Reviewer #2

(Remarks to the Author)
This manuscript employs replicative senescence in IMR90 fibroblasts as a model system. By integrating quantitative mass spectrometry-based proteomics with complementary approaches—including analysis of the ubiquitin-modified proteome, solubility profiling, and proteostasis perturbations—the study maps time-resolved proteome remodeling during senescence. The authors propose that these changes are coordinated, encompassing broad depletion of chromatin-associated proteins, downregulation of the cytosolic translation machinery, increased insolubility of mitochondrial proteins, and compromised autophagy and proteasome activity. These alterations are reportedly accompanied by a remodeling of ubiquitin linkage types and E3 ligase networks.

Major Comments
1. Critical inconsistency in the definition of "age" or "timepoint" labels. This issue could undermine all temporal interpretations presented in the study. In the Methods section, "age 1" is defined as <30 cumulative population doublings (PDs) and "age 4" as >60 PDs. However, the same section subsequently identifies intermediate timepoints as "age 3 and age 4" at approximately 40 and 50 ± 2 PDs, respectively. This creates a direct conflict where "age 4" refers both to an intermediate stage (~50 PD) and the late senescent endpoint (>60 PD). The authors must:
o (i) Correct and standardize the mapping between "age" labels and specific PD ranges.
o (ii) Audit all figures, tables, and statistical contrasts that utilize these labels to ensure consistency.
o (iii) Provide a clear sample sheet detailing the PD count for each sample and confirm senescence markers aligned with the corrected labeling scheme.
2. The experimental replication structure and statistical independence require clearer reporting. The manuscript mentions inclusion criteria based on quantification across "technical replicates," but it remains ambiguous how many biological replicates (independent cell cultures) were used per timepoint/condition, as opposed to repeated injections or measurements of the same sample preparation. Given the sensitivity of senescence time courses to batch and culture effects, the authors should:
o Explicitly distinguish between biological and technical replicates.
o Clearly state the number of biological replicates (n) for each experiment and timepoint.
o Clarify how statistical analyses accounted for sample independence and whether potential correlations within the time course or across batches were considered.
3. Conclusions regarding proteostasis function would benefit from tighter control of confounding factors and clearer quantification logic. The authors conclude that both proteasome and autophagy functions are compromised during senescence and report a concomitant decrease in ATP levels. Since ATP depletion can itself confound luminescence-based activity assays and their normalization, the manuscript would be strengthened by:
o Explicitly stating the normalization strategy employed for activity assays (e.g., per cell number, total protein amount, or with ATP-level correction).
o Demonstrating the assay dynamic range using appropriate positive controls (e.g., MG132 for proteasome inhibition).
o Clarifying how the observed changes in cellular ATP levels were accounted for when comparing enzymatic activities across different senescence states.
4. Western blot data in Figures S2B and S2D require statistical quantification. Figure S2 presents representative western blot images (e.g., for LC3B), and the manuscript cites western blotting in Figure S2D as confirmation of proteasome/immunoproteasome subunit changes. To meet standard reporting rigor, the authors should:
o Add densitometric quantification for these blots.
o Apply appropriate normalization controls.
o Report the number of biological replicates (n) and provide statistical testing of the differences observed (including exact p-values where possible).
5. A limited set of orthogonal validations for key discoveries would substantially strengthen confidence. The study's central findings—chromatin protein depletion, mitochondrial ribosome protein insolubility, and K27-linkage-associated remodeling—are compelling but heavily reliant on mass spectrometry data. Incorporating a focused validation set would greatly enhance reader confidence. For instance, the authors could validate:
o (i) 2–3 representative proteins from depleted chromatin-associated complexes.
o (ii) 1–2 mitochondrial ribosomal proteins shown to shift towards the insoluble fraction.
o (iii) One or two factors associated with K27-linked ubiquitination.
Validation via independent methods such as immunofluorescence (IF) and/or western blotting (WB) across early versus late senescence stages, accompanied by quantification, would be highly impactful even if limited in scope.
6. Generality of the findings across different senescence models. While IMR90 fibroblasts are a well-established model for replicative senescence, the conclusions drawn are broad. To better contextualize the generalizability of the findings, the authors should include a brief discussion on:
o The potential cell-type specificity of the observed proteomic changes.
o How these changes might compare or contrast with those reported in other models of senescence (e.g., stress-induced or oncogene-induced senescence).
Minor Comments

1. Terminology, typographical errors, and label consistency.
o Please correct "trypin-like" to "trypsin-like."
o Ensure consistent nomenclature throughout the manuscript for terms like "age 1–4," "proliferative/senescent," and "intermediate timepoints," particularly after resolving the age-PD mapping issue.
2. Clustering strategy and robustness of temporal patterns. K-means clustering is central to defining temporal proteome trajectories. However, the rationale for selecting the specific number of clusters (k) and an assessment of the clustering results' stability are not fully described. Providing cluster validation metrics (e.g., silhouette scores) or sensitivity analyses (e.g., clustering with different k values) would bolster confidence that the inferred temporal patterns are robust and not overly dependent on the chosen methodological parameters.
3. Extensive reliance on Z-score normalization across datasets. Many key analyses (clustering, heatmaps, violin plots, enrichment) utilize Z-score normalization. While useful for visualization, this approach can obscure absolute effect sizes and complicate comparisons across conditions or with other omics datasets. The manuscript would benefit from:
o Clarifying how Z-scores were computed (e.g., globally across all samples or within specific conditions).
o Briefly addressing whether the core biological conclusions remain robust when alternative normalization strategies are applied, thereby enhancing computational rigor.

(Remarks on code availability)

Version 1:

Reviewer comments:

Reviewer #1

(Remarks to the Author)
The authors have properly addressed my comments and solidified their claims regarding proteoasome activity (new figures 5, G and H). They also expanded their exploration of SUMOylation in the context of cell senescence (new figures 4, C and D). The new data is discussed in the revised manuscript.
I get the impression that they also comprehensively addressed the critique of referee #2.
In conclusion, I think that the manuscript is much clearer and should be published in its current form.

(Remarks on code availability)

Reviewer #2

(Remarks to the Author)
I would like to thank the authors for their comprehensive and constructive response to the review comments. The revised manuscript has been significantly strengthened. However, one major issue regarding the generality of the findings across different senescence models remains insufficiently resolved:
In their response to the query regarding the generalizability of the findings (Reviewer #2, Major Comment 6), the authors argue for the existence of a "core senescence program" by referencing transcriptomic and single-cell RNA-seq literature and have updated the discussion on page 26. While this conceptual framing is valuable, it relies almost exclusively on transcript-level data. Given that this study is fundamentally rooted in quantitative proteomics, protein solubility profiling, and post-translational modifications, transcriptomic conservation does not automatically translate to proteomic or PTM-level conservation. To fully resolve this point, the authors should further refine the discussion in the manuscript to:
1. Discuss how their specific proteomic, solubility, and PTM (ubiquitylome/SUMOylome) signatures compare or contrast with existing proteomic datasets of other senescence models, such as oncogene-induced senescence (OIS) or stress-induced premature senescence (SIPS), rather than relying solely on mRNA-level literature.
2. Explicitly address the limitation that a transcriptionally defined "core senescence program" may not fully capture the conservation or divergence of the senescent proteome and PTM networks across different cellular contexts and induction methods.
Once this final point is adequately addressed in the text, the manuscript should be well-suited for publication.

(Remarks on code availability)

Version 2:

Reviewer comments:

Reviewer #2

(Remarks to the Author)
The authors have addressed all of my questions and comments satisfactorily. The manuscript is now suitable for publication in the journal, and I have no further comments.

(Remarks on code availability)

Open Access This Peer Review File is licensed under a Creative Commons Attribution 4.0 International License, which permits use, sharing, adaptation, distribution and reproduction in any medium or format, as long as you give appropriate credit to the original author(s) and the source, provide a link to the Creative Commons license, and indicate if changes were made.
In cases where reviewers are anonymous, credit should be given to 'Anonymous Referee' and the source.
The images or other third party material in this Peer Review File are included in the article's Creative Commons license, unless indicated otherwise in a credit line to the material. If material is not included in the article's Creative Commons license and your intended use is not permitted by statutory regulation or exceeds the permitted use, you will need to obtain permission directly from the copyright holder.
To view a copy of this license, visit https://creativecommons.org/licenses/by/4.0/

Revision of manuscript "*Quantitative proteomics reveals coordinated changes in the proteome during replicative senescence*"

Response to the reviewers' comments

We would like to thank the reviewers for their constructive criticism. In response to their helpful comments, we have made extensive revisions to the manuscript, both by including additional experimental data and by re-analyzing some data, rephrasing unclear passages, and adding a discussion of critical points. Our principal experimental revisions relate to an expansion of the analysis of protein SUMOylation and the effects of proteasome inhibition, an improvement of proteasomal activity measurements by considering the effects of cellular ATP levels, a careful validation of cross-sample comparison, and an orthogonal verification of changes in the levels of relevant proteins via quantitative western blotting.

Reviewer #1 (Remarks to the Author):

Cellular senescence is characterized by various changes that impair functions of multiple mechanisms and result in cell enlargement and eventually death. With aging, the removal efficiency of senescent cells deteriorates, and in some cases underlie an aging-associated increase in the incidence of different types of cancer and other disorders. Accordingly, enhancing the removal of senescent cells slows organismal aging and promotes health span (Yosef et al., Nat Commun. 2016 Apr 6;7:11190).

In this study, the authors used cultured human lung fibroblasts (IMR90) and mass spectrometry techniques, to characterize the proteomic, ubiquitin modifications and protein solubility signatures of senescent cells.

In a nutshell they discovered that during senescence, cells exhibit modified levels of proteins that reside in different cellular localizations, however, nuclear proteins were overrepresented among these which showed reduced levels. These include proteins that are linked with DNA structure, transcription and RNA processing. Functional clustering showed that proteins that are associated with protein degradation mechanisms, namely the ubiquitin-proteasome system (UPS) and autophagy, show modified levels. While UPS-linked proteins, and especially E3 ubiquitin ligases, showed reduced levels, those which are involved in autophagy exhibited increased levels. In agreement, UPS activity showed reduced levels in senescent cells compared to proliferative cells. Using biochemical techniques and mass spectrometry they found that more proteins are present in insoluble state in senescent cells than in proliferative cells. Among these, mitochondrial and K27-ubiquitinated proteins were over-represented. Finally, they stressed the cells using various compounds that inhibit the UPS, autophagy, HSP90 or induce oxidative stress, and found that senescence elicits proteome modifications that are distinct from the tested stress responses.

These findings are similar to the recent analysis of aging-associated proteomic changes that were characterized in the Killifish brain (Di Fraia et al., 2025, reference #43). This study provides a "proteomic atlas" of senescent IMR90 cells, that points at specific cellular mechanisms to be majorly affected by senescence. Thus, it could be an interesting and important source for researchers in the field. Yet, several issues should be addressed to enhance rigor and enhance the manuscript.

1. *To inhibit proteasome activity the authors used MG132 (Fig. 5). While this is a widely used compound, MG132 also inhibits calpains. To better establish their conclusions and enhance the confidence that the*

observed proteomic changes stem from UPS inhibition, they should repeat this analysis using an additional, irreversible proteasome inhibitor such as Epoxomicin or Bortezomib (as they did to stress the cells – Fig. 7).

To ensure the reproducibility of our findings, as suggested, we have complemented our analysis using another proteasomal inhibitor (8 µM Bortezomib, 4 hours) and have compared its effects to those of MG132. To ensure dataset comparability, we applied a False Discovery Rate (FDR) < 0.05 and a quantile cutoff of > 0.9 to each dataset. Only proteins meeting these criteria were classified as significantly regulated in either proliferative or senescent conditions. From the resulting pool of significantly regulated proteins, we focused our analysis on the subset of proteins that were commonly upregulated (i.e. increased degradative ubiquitylation) across both treatments. This analysis revealed that proliferative and senescent cells shared around 76% of proteins with increased degradative ubiquitylation, with only 12% unique to senescent or proliferative conditions (new Figure 5G). Furthermore, among proteins that show increased degradative ubiquitylation only in senescent cells, we found an enrichment of proteins associated with the GO MF term "catalytic activity" (new Figure 5H).

2. One of the interesting conundrums that the authors examined is how senescence modulates post translational modifications (PTMs). Their results show that ubiquitination- and SUMOylation-associated proteins (but not UFMylation) are most prominently affected by senescence. Specifically, SUMO1 and SUMO2 show senescence-associated reduction which is similar to these of E3 ubiquitin ligases (Fig. 4a). Given that SUMOylation has been shown to regulate aging and proteostasis (Princz et al., Sci Rep. 2020 Sep 23;10(1):15513 and Moll et al., Elife. 2018 Nov 7;7:e38635) it is important that the authors follow this interesting direction and explore which proteins exhibit modulated SUMOylation levels in senescent cells. It is also advised that they will discuss the roles of SUMOylation in the discussion.

Following the reviewer's suggestion, we have now significantly expanded our analysis of protein SUMOylation. Considering that both SUMO1 and SUMO2/3 are depleted during senescence, and that SUMO2/3 typically exhibits a significantly higher intracellular concentration compared to SUMO1 and is known to regulate cellular stress responses[1], we decided to focus on SUMO2/3-modified proteins. To also gain information about effects on protein solubility, we performed pulldowns using an anti-SUMO2/3 antibody from lysates obtained at high SDS concentration.

Our SUMO2/3-enriched samples showed good intra-replicate correlation (new Figure S3C). Pulldowns were performed using equivalent protein amounts (new Figure S3D). Our analysis shows an enrichment of 122 SUMO2/3-modified proteins in senescence, most of them known targets of SUMO, such as nuclear body proteins PML, SON and SRRM2 (new Figure 4B, D). Furthermore, GO term analysis demonstrates an enrichment of proteins associated with protein translation such as cytoplasmic ribosomes and nucleosome organization (new Figure 4C, D). These new findings show that the cytoplasmic translation machinery experiences decreased ubiquitylation (Figure 5C) but increased SUMOylation in senescence (new Figure 4E). Using immunofluorescence and confocal microscopy, we show that the changes in PML SUMOylation in senescence go along with changes in the number and size of PML bodies (new Figure S3E). We describe and discuss these findings in the revised manuscript on pages 13-14 and 25-26, respectively.

3. An additional important aspect that could enhance the study is a comparison of proteomic changes in senescent cells and additional organisms (in addition to the Killifish). This can be done by comparing the

proteomic changes to these which were observed in the context of aging in the nematode C. elegans. One relevant comparison is of the list of senescent cells-specific insoluble proteins (Fig. 6) with protein that were found by the Kenyon lab to sediment in aged nematodes (David et al., PLoS Biol. 2010 Aug 10;8(8):e1000450). Another comparison should be done between the differentially Ubiquitinated proteins (Fig. 6) and the proteins that were found by the Vilchez lab to exhibit modulated ubiquitination levels in elder worms Koyuncu et al., Nature. 2021 Jul 28;596(7871):285–290). Such comparison will address the question of how general the reported findings are.

To better contextualize our findings, we have attempted to directly compare our datasets with the suggested datasets. A prerequisite for this attempt is the assignment of human orthologues for the proteins identified in those studies. To achieve this, we relied on the *BiocManager* and *biomaRt* packages for R studio, considering only "ortholog_one2one" or "ortholog_one2many" types. Taking into account gene duplication events reported between *Nothobranchius furzeri* /*Caenorhabditis elegans* and *Homo sapiens*, we used orthologous groups to perform this comparison and subsequent statistical analysis.

We started by evaluating which proteins become more insoluble in senescence and during nematode aging (Della *et al*., 2010)[2]. With this approach, we identified a total of 267 orthologous groups between the two species. Of these, only 20 orthologous groups were found to become more insoluble in both species. These were mostly mitochondria-related proteins as well as proteins participating in the unfolded protein response (Revision Table 1). However, the observed overlap was not statistically significant (Hypergeometric test of 0.819), thus possibly representing a stochastic occurrence and attributable to the small overall numbers. For that reason, we would prefer not to include this comparison in the manuscript.

Revision Table 1 | List of proteins found to be insoluble in senescent cells (this study) and aged *C. elegans*[2].

Orthologous group	Human ortholog	C. elegans ortholog	Ortholog type
1	DLD	dld-1	ortholog_one2one
2	PDHB	pdhb-1	ortholog_one2one
3	HSP90B1	enpl-1	ortholog_one2one
4	MMUT	mmcm-1	ortholog_one2one
5	AHCY	ahcy-1	ortholog_one2one
6	PSMA5	pas-5	ortholog_one2one
7	OAT	oatr-1	ortholog_one2one
8	DUT	dut-1	ortholog_one2one
9	HSPA9	hsp-6	ortholog_one2one
10	NUTF2	ran-4	ortholog_one2one
11	PRDX4	prdx-3	ortholog_one2one
12	DHTKD1	ogdh-2	ortholog_one2one
13	ACO2	aco-2	ortholog_one2one
14	MRPL44	mrpl-44	ortholog_one2one
15	RAN	ran-1	ortholog_one2one
16	PDLIM1	alp-1	ortholog_one2many
17	ENO1	enol-1	ortholog_one2many
17	ENO2	enol-1	ortholog_one2many
18	ME2	men-1	ortholog_one2many
19	CALR	crt-1	ortholog_one2many
20	GRPEL1	C34C12.8	ortholog_one2many

A comparative analysis of the ubiquitylome data during IMR90 senescence and aging *C. elegans*[3] was challenging for technical reasons, due to differences between the statistical analyses adopted in the two studies. Koyuncu *et al*.[3] did not normalize ubiquitylation levels to their corresponding protein levels; instead, statistical analyses for the ubiquitylome and the proteome were performed separately and subsequently integrated. However, since individual protein levels are changing during aging, normalization needs to be included to address whether proteins are indeed differentially ubiquitylated or only changing in their abundance. As we were unable to confirm that the same samples were used across datasets (ubiquitylome and proteome), a direct comparison of our data with the published literature was therefore not feasible.

In another attempt to address the reviewer's comment, we extracted the data from Supplementary Table 5 of Koyuncu *et al*. and attempted to compare it with our data (Revision Table 2). Ortholog searches between *C. elegans* and human proteins yielded 341 orthologous groups within our set of proteins commonly modified by ubiquitin during aging and senescence. Of these, only 30 orthologous groups exhibited significant alterations during IMR90 senescence and *C. elegans* aging. Most of the compared orthologous groups exhibited decreased ubiquitylation in *C. elegans* and either a downregulation (n=11) or no significant change (n=13) in protein levels. Most of these were ribosomal proteins, suggesting that regulation occurs at the post-translational level. This could be indicative of a decreased abundance of cytoplasmic translation machinery common between human cellular senescence and *C. elegans* organismal aging, which should be clarified in further studies, but the basis on which such a statement could be made appears insufficiently rigorous to be included in the manuscript. In the revised manuscript, we therefore only discuss commonly regulated processes found in our study and aged vertebrates (pages 24 to 25).

Revision Table 2 | List of proteins found to be differentially ubiquitylated in senescent cells (this study) and aged *C. elegans*[3].

Symbol (*H. sapiens*)	Regulation in senescence (Protein Group)	WormBase ID	Ortholog type	Regulation of ubiquitylated peptides in aged worms
RPL18	Decreased ubiquitylation	rpl-18	ortholog_one2one	Ub down, protein down
RPL23A	Decreased ubiquitylation	rpl-25.2	ortholog_one2many	Ub down, protein down
RPL24	Decreased ubiquitylation	rpl-24.1	ortholog_one2one	Ub down, protein down
RPL35	Decreased ubiquitylation	rpl-35	ortholog_one2one	Ub down, protein down
RPL35A	Decreased ubiquitylation	rpl-33	ortholog_one2one	Ub down, protein down
RPL5	Decreased ubiquitylation	rpl-5	ortholog_one2one	Ub down, protein down
RPS18	Decreased ubiquitylation	rps-18	ortholog_one2one	Ub down, protein down
RPS6	Decreased ubiquitylation	rps-6	ortholog_one2one	Ub down, protein down
RPS8	Decreased ubiquitylation	rps-8	ortholog_one2one	Ub down, protein down

UBAP2L	Decreased ubiquitylation	pqn-59	ortholog_one2many	Ub down, protein down
UTP20	Decreased ubiquitylation	utp-20	ortholog_one2one	Ub down, protein down
DYNLL1	Decreased ubiquitylation	dlc-1	ortholog_one2many	Ub down, protein equal
RPL14	Decreased ubiquitylation	rpl-14	ortholog_one2one	Ub down, protein equal
RPL15	Decreased ubiquitylation	rpl-15	ortholog_one2one	Ub down, protein equal
RPL27	Decreased ubiquitylation	rpl-27	ortholog_one2one	Ub down, protein equal
RPL3	Decreased ubiquitylation	rpl-3	ortholog_one2many	Ub down, protein equal
RPL31	Decreased ubiquitylation	rpl-31	ortholog_one2one	Ub down, protein equal
RPL8	Decreased ubiquitylation	rpl-2	ortholog_one2one	Ub down, protein equal
RPS13	Decreased ubiquitylation	rps-13	ortholog_one2one	Ub down, protein equal
RPS15	Decreased ubiquitylation	rps-15	ortholog_one2one	Ub down, protein equal
RPS2	Decreased ubiquitylation	rps-2	ortholog_one2one	Ub down, protein equal
RPS3A	Decreased ubiquitylation	rps-1	ortholog_one2one	Ub down, protein equal
RPS9	Decreased ubiquitylation	rps-9	ortholog_one2one	Ub down, protein equal
NOP56	Decreased ubiquitylation	nol-56	ortholog_one2one	Ub down, protein up
NOP58	Decreased ubiquitylation	nol-58	ortholog_one2one	Ub down, protein up
RPL7L1	Decreased ubiquitylation	rpl-7	ortholog_one2many	Ub down, protein up
VAMP2	Increased ubiquitylation	snb-1	ortholog_one2one	Ub down, protein up
RPF1	Decreased ubiquitylation	F44G4.1	ortholog_one2one	Ub up, protein equal
RPL13	Decreased ubiquitylation	rpl-13	ortholog_one2one	Ub up, protein equal
RPS2	Decreased ubiquitylation	rps-2	ortholog_one2one	Ub up, protein equal
ABCD4	Increased ubiquitylation	pmp-5	ortholog_one2many	Ub up, protein equal
RPL7L1	Decreased ubiquitylation	rpl-7	ortholog_one2many	Ub up, protein up
VAMP2	Increased ubiquitylation	snb-1	ortholog_one2one	Ub up, protein up

Reviewer #1 (Remarks on code availability):

I do not have the skills to review computational codes.

Reviewer #2 (Remarks to the Author):

This manuscript employs replicative senescence in IMR90 fibroblasts as a model system. By integrating quantitative mass spectrometry-based proteomics with complementary approaches—including analysis of the ubiquitin-modified proteome, solubility profiling, and proteostasis perturbations—the study maps time-resolved proteome remodeling during senescence. The authors propose that these changes are coordinated, encompassing broad depletion of chromatin-associated proteins, downregulation of the cytosolic translation machinery, increased insolubility of mitochondrial proteins, and compromised autophagy and proteasome activity. These alterations are reportedly accompanied by a remodeling of ubiquitin linkage types and E3 ligase networks.

Major Comments

1. Critical inconsistency in the definition of "age" or "timepoint" labels. This issue could undermine all temporal interpretations presented in the study. In the Methods section, "age 1" is defined as <30 cumulative population doublings (PDs) and "age 4" as >60 PDs. However, the same section subsequently identifies intermediate timepoints as "age 3 and age 4" at approximately 40 and 50 ± 2 PDs, respectively. This creates a direct conflict where "age 4" refers both to an intermediate stage (~50 PD) and the late senescent endpoint (>60 PD). The authors must:

o (i) Correct and standardize the mapping between "age" labels and specific PD ranges.
o (ii) Audit all figures, tables, and statistical contrasts that utilize these labels to ensure consistency.
o (iii) Provide a clear sample sheet detailing the PD count for each sample and confirm senescence markers aligned with the corrected labeling scheme.

We apologize for the confusion that may have arisen from a typographical error in the methods section of the original manuscript. We have now corrected this error and ensured that the nomenclature for the stages is consistent throughout the revised manuscript. For all experimental procedures we define early-passage IMR90 cells (Proliferative, age 1) as having undergone <32 cumulative population doublings, and late-passage cells (Senescent, age 4) as having undergone >60 cumulative population doublings. For time-course experiments, two intermediate stages were also taken for analysis: age 2 and age 3, with cumulative population doublings of 40 and 50 ± 2, respectively. We have now included this information in the schematics of Figure 1A, in the figure legends and in the Materials and Methods section.

In this context, we would like to point out that although the population doublings serve as an important "tracker" of replicative senescence, the senescence state is defined more accurately though internal controls in each experiment. To tackle this, we validated the senescence phenotype by monitoring established senescence biomarkers (Figure S5D) and confirmed that expression patterns for these were consistent across all experimental replicates and experiments conducted.

2. The experimental replication structure and statistical independence require clearer reporting. The manuscript mentions inclusion criteria based on quantification across "technical replicates," but it remains ambiguous how many biological replicates (independent cell cultures) were used per timepoint/condition, as opposed to repeated injections or measurements of the same sample preparation. Given the sensitivity of senescence time courses to batch and culture effects, the authors should:

o Explicitly distinguish between biological and technical replicates.
o Clearly state the number of biological replicates (n) for each experiment and timepoint.
o Clarify how statistical analyses accounted for sample independence and whether potential correlations within the time course or across batches were considered.

In the revised manuscript, we now include information regarding the growth curves and the age replicates that were used in the analysis. A detailed description of the experimental procedures has been added to the methods section (page 37) and well-defined terms are used in all the main and supplementary figures and legends.

To assess potential batch effects, we performed a rigorous assessment of correlations across experiments. We chose to proceed with a data-dependent acquisition method for mass spectrometry, relying on TMTpro™ quantification and off-line fractionation[4,5,6]. This method is advantageous for this experimental set up, as it allows for (1) the combination and quantification of a maximum number of 16 samples per run, (2) elimination of missing values in protein quantification, (3) enhanced precision and sensitivity and (4) deep protein coverage when used together with off-line fractionation. Following analysis in MaxQuant, the extracted data was subjected to the variance stabilization normalization algorithm, for correction of batch effects. After these steps, we employed dimensional reduction techniques such as PCA, as well as unsupervised learning algorithms such as hierarchical clustering, to check whether batch effects were predominant in the dataset (Revision Figure 1A, B). From these data, we concluded that all three growth curves were sufficiently similar for a successful integration of the proteomes. For subsequent evaluation of post-translational modifications and alterations on protein solubility, we therefore relied on the data of one growth curve while maintaining a minimal number of 3 independent age replicates for each stage, with no repeated injections as technical replicates being performed. For whole proteome analysis, we present data from all three growth curves, each performed in four age replicates.

Revision Figure 1

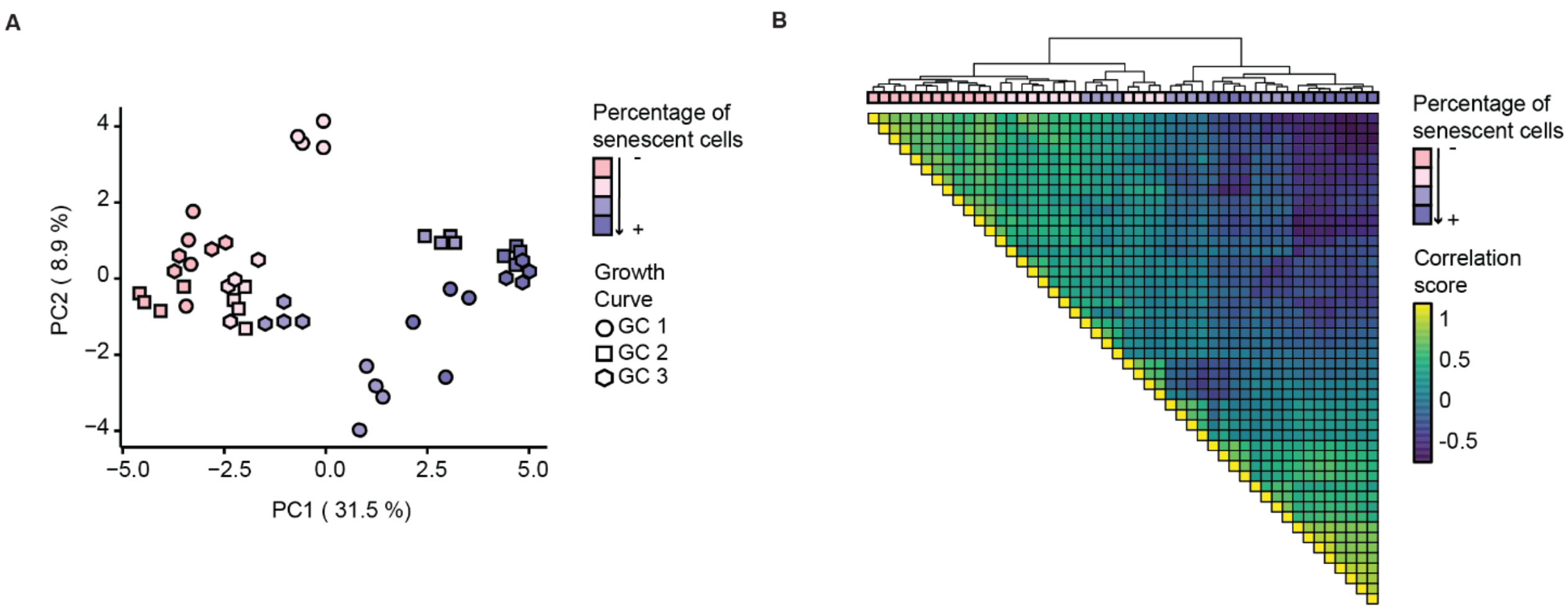

Revision Figure 1 | Quality control analysis of the proteome samples demonstrates a high reproducibility across the three independent IMR90 growth curves analyzed in this study and among age replicates. (A) Principal component analysis of protein expression profiles for the samples used in the characterization of proteome changes during senescence. (B) Heatmap displays hierarchical clustering and Pearson correlation scores for the samples used for the characterization of proteome changes during senescence progression.

3. Conclusions regarding proteostasis function would benefit from tighter control of confounding factors and clearer quantification logic. The authors conclude that both proteasome and autophagy functions are compromised during senescence and report a concomitant decrease in ATP levels. Since ATP depletion can itself confound luminescence-based activity assays and their normalization, the manuscript would be strengthened by:

o Explicitly stating the normalization strategy employed for activity assays (e.g., per cell number, total protein amount, or with ATP-level correction).

o Demonstrating the assay dynamic range using appropriate positive controls

o Clarifying how the observed changes in cellular ATP levels were accounted for when comparing enzymatic activities across different senescence states.

Based on these concerns, we have repeated the quantification of ATP and proteasomal activities in proliferative and senescent conditions more rigorously. To evaluate which normalization strategy would be most adequate, we started by monitoring for each condition the total protein content as a function of cell number. In this manner, it became obvious that a 1.5× to 2× fold higher amount of protein can be extracted from the same number of senescent cells compared to proliferative cells (Revision Figure 2A). This increase in total protein content is known to occur, as senescent cells undergo massive hypertrophy, reaching 3 to 5 times the size of proliferative cells. To take this into account, we decided to use total protein content rather than cell number as a basis for the normalization of both assays.

To account for differences in basal ATP levels in proliferative and senescent conditions, we then blocked respiratory ATP production by treating the cells with 5 µM FCCP, an uncoupler of oxidative phosphorylation. To avoid ATP production through glycolysis, cells were cultured in media without glucose (PBS supplemented with calcium and magnesium). Under these conditions, we indeed observed a

depletion of ATP. In senescent cells, the effect was weaker, most likely because these cells rely more on glycolysis compared to proliferative cells. In the revised manuscript, we have added this data as a new Figure 3F.

Using the same experimental conditions, we then assessed differences in proteasomal activity in proliferative and senescent conditions. This assay was complemented with a control (treatment with proteasome inhibitor), and these data are now included in the revised manuscript in new Figure 3E (information regarding statistical testing can be found in the source data supplement). The activity assays show decreased Chymotrypsin-, Trypsin- and Caspase-like proteasomal activities in senescent compared to proliferative cells. However, the effect of ATP depletion on proteasomal activity turned out to be minor (proliferative cells – no significant change; senescent cells – caspase-like activity: 0.8 relative to control; trypsin-like activity: 1.32 relative to control). We therefore conclude that ATP depletion is not responsible for the observed decrease in the activity of the proteasome in senescent cells. We have included this data in the revised manuscript on page 11.

Revision Figure 2

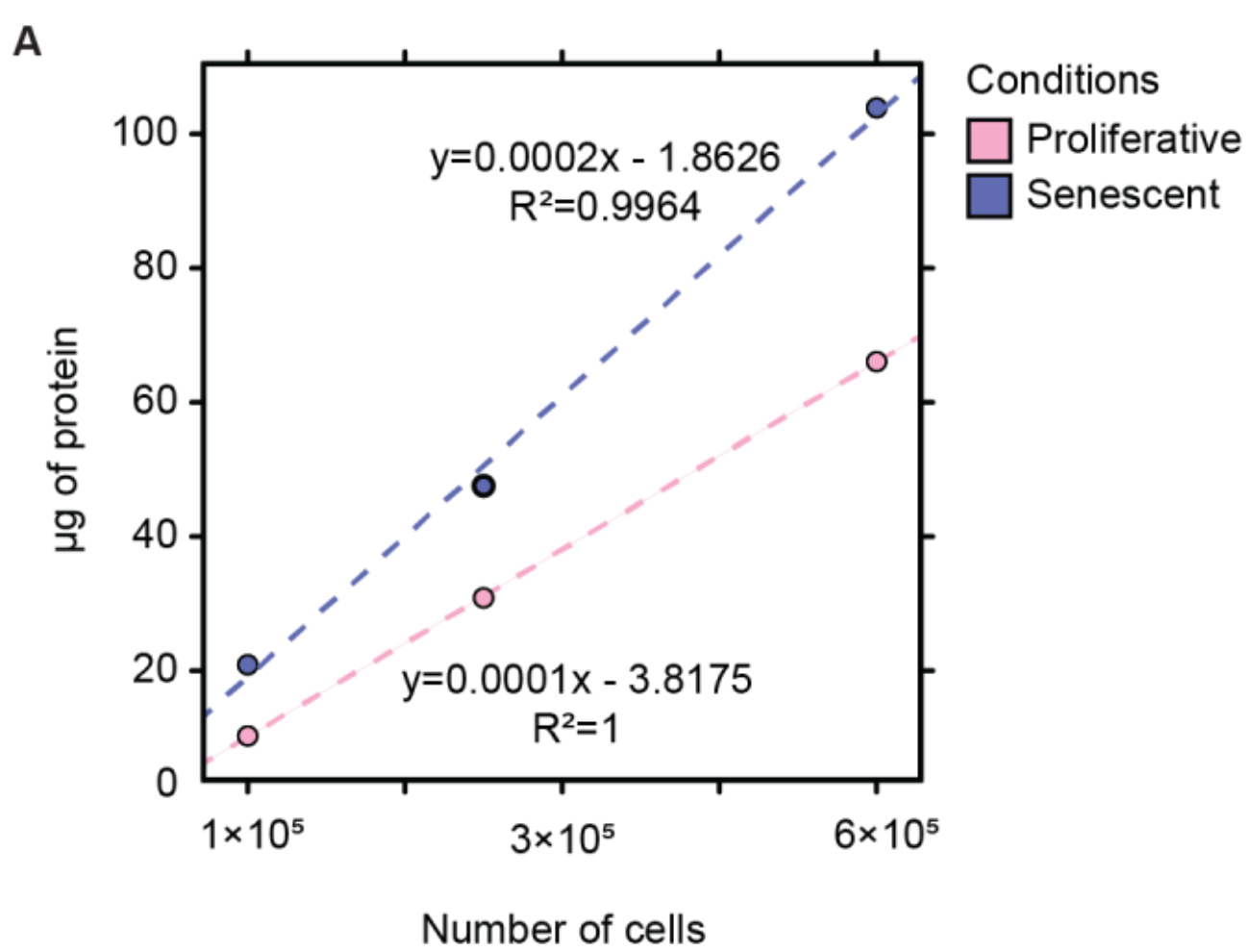

Revision Figure 2 | Senescent cells exhibit almost double the amount of protein content, when in comparison with proliferative cells, while displaying lower levels of ATP and proteasomal activity. (A) Scatter plot displaying the correlation between cell number and protein yield for proliferative and senescent conditions. Dashed lines represent the linear regression for each condition, with the corresponding equations for the best fit lines and R^2 values being displayed on the graph.

4. Western blot data in Figures S2B and S2D requires statistical quantification. Figure S2 presents representative western blot images (e.g., for LC3B), and the manuscript cites western blotting in Figure S2D as confirmation of proteasome/immunoproteasome subunit changes. To meet standard reporting rigor, the authors should:
o Add densitometric quantification for these blots.
o Apply appropriate normalization controls.

o Report the number of biological replicates (n) and provide statistical testing of the differences observed (including exact p-values where possible).

We have now added representative western blot images for PSMB10, PSMB8 and PSMC3 (Figure S2C). Levels of protein expression were normalized to total protein levels as determined by Ponceau S staining. Bar plots displaying densitometric quantification of these blots were added to Figure 3H for PSMB10 and PSMB8, while Figure S2F displays PSMC3 data.

Information regarding the number of age replicates was also added to the legend of each figure.

5. A limited set of orthogonal validations for key discoveries would substantially strengthen confidence. The study's central findings—chromatin protein depletion, mitochondrial ribosome protein insolubility, and K27-linkage-associated remodeling—are compelling but heavily reliant on mass spectrometry data. Incorporating a focused validation set would greatly enhance reader confidence. For instance, the authors could validate:
o (i) 2–3 representative proteins from depleted chromatin-associated complexes.
o (ii) 1–2 mitochondrial ribosomal proteins shown to shift towards the insoluble fraction.
o (iii) One or two factors associated with K27-linked ubiquitination.
Validation via independent methods such as immunofluorescence (IF) and/or western blotting (WB) across early versus late senescence stages, accompanied by quantification, would be highly impactful even if limited in scope.

The revised manuscript now includes quantifications of representative proteins of chromatin-associated complexes (POL2, XRCC1 and SMC2) as well as two representative mitochondrial ribosome subunits that become more insoluble during senescence (MRPL50 and MRPL18). Total protein content (Ponceau S staining) was used as a normalization control. Representative western blot images and densitometric quantification can be found in Figure S1E and Figure S4C, respectively.

Validation of factors associated with K27-Linked ubiquitination is more challenging due to the current lack of K27-linkage specific tools. However, a recent bioRxiv preprint reports the mapping of the K27-linked ubiquitin landscape under proteotoxic stress by means of a new K27-linkage conformation-specific antibody, identifying molecular chaperones enriched in the K27-linked ubiquitin fraction[7]. These findings could well be linked to our observation of K27-linkage enrichment in the insoluble protein fraction. We have therefore added this information in the revised manuscript (page 20).

6. Generality of the findings across different senescence models. While IMR90 fibroblasts are a well-established model for replicative senescence, the conclusions drawn are broad. To better contextualize the generalizability of the findings, the authors should include a brief discussion on:
o The potential cell-type specificity of the observed proteomic changes.
o How these changes might compare or contrast with those reported in other models of senescence (e.g., stress-induced or oncogene-induced senescence).

Elucidation of which molecular features are core to the senescent phenotype remains challenging, even with access to state-of-the-art multi-omics methods. It is consensual in the literature that a core senescence program exists, as senescence has been postulated to be an evolutionary conserved program

with relevance for protection against cancer progression as well as for embryogenesis and tissue homeostasis.

First indications that a core response does exist came from the integration of transcriptomics datasets of established cellular senescence models[8]. This study uncovered a transcriptomic core signature for senescence for different models of cellular senescence, following distinct senescence induction methods. Others have started to characterize senescent cells using single-cell omics approaches, further corroborating the well-known fact that senescent cells exhibit a great phenotypic variation[9]. This suggests that albeit there is a core senescence response, this should be viewed not as a static program, but instead as a dynamic footprint that is context-, time- and cell-type specific. Thus, to better contextualize our findings, we have now included in the revised manuscript a statement about the variability of the senescent phenotype and the existence of a core senescence response, on page 26.

Minor Comments

1. Terminology, typographical errors, and label consistency.
o Please correct "trypin-like" to "trypsin-like."
o Ensure consistent nomenclature throughout the manuscript for terms like "age 1–4," "proliferative/senescent," and "intermediate timepoints," particularly after resolving the age-PD mapping issue.

We have now corrected typographical errors, including one that caused confusion about the description of "timepoints", and we have ensured uniform nomenclature for these stages and samples throughout the paper. The revised manuscript now includes a definition of aging stages based on the cumulative population doubling of the cells and an assignment of sample identity. Statements about the number of independent age replicates are now included in all figure legends.

2. Clustering strategy and robustness of temporal patterns. K-means clustering is central to defining temporal proteome trajectories. However, the rationale for selecting the specific number of clusters (k) and an assessment of the clustering results' stability are not fully described. Providing cluster validation metrics (e.g., silhouette scores) or sensitivity analyses (e.g., clustering with different k values) would bolster confidence that the inferred temporal patterns are robust and not overly dependent on the chosen methodological parameters.

To determine the adequate number of clusters for each analysis, we started by performing different cluster validation methods, such as the Elbow, Silhouette and Gap methods (Revision Figure 3A). According to these analyses, the optimal number of clusters would be 2. However, this limited number of clusters would not capture subtle differences in temporal patterns besides increases and decreases in protein levels. The second-best number of clusters is 6, as determined by maximum silhouette width. However, when examining the clusters obtained in this manner, some of them exhibited very similar patterns (Revision Figure 3B). We therefore decided to combine these, yielding a total of 4 clusters. We now include this justification in the revised manuscript.

Revision Figure 3

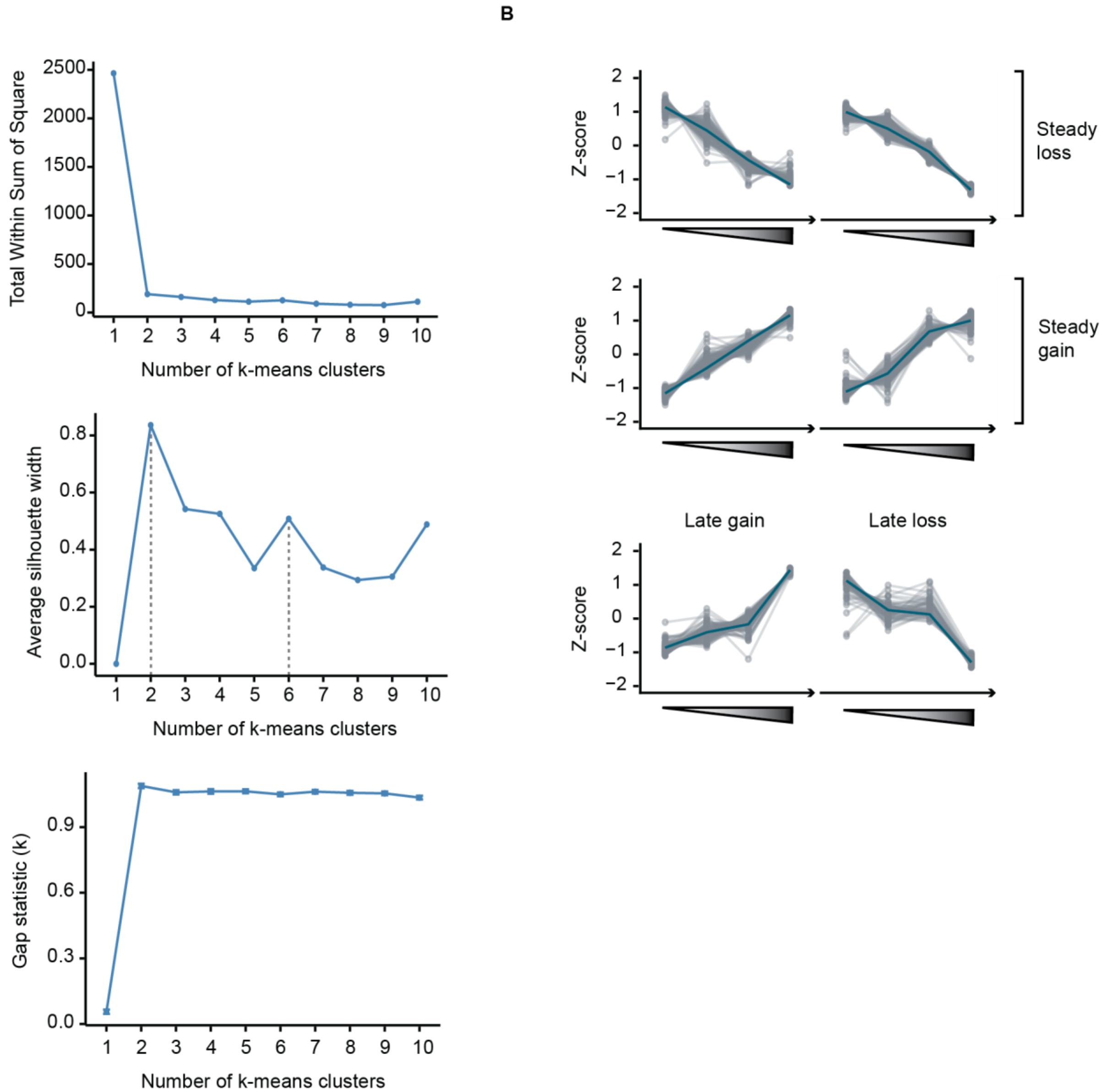

Revision Figure 3 | Determination of the optimal K-means clusters used for the characterization of temporal patterns during senescence progression. (A) Line plots displaying scores for distinct statistical tests used in the determination of the optimal number of k-means clusters for the dataset. Top: the Total Within Sum of Squares plotted against the number of clusters – Elbow method. Middle: average silhouette width analysis for each cluster number – Silhouette method. Grey dashed lines indicate from left to right the first- and second-best scoring number of clusters. Bottom: gap statistic plotted across varying cluster numbers. (B) Differentially regulated proteins were clustered using the K-means algorithm, showing the patterns obtained when six clusters are used.

3. Extensive reliance on Z-score normalization across datasets. Many key analyses (clustering, heatmaps, violin plots, enrichment) utilize Z-score normalization. While useful for visualization, this approach can obscure absolute effect sizes and complicate comparisons across conditions or with other omics datasets.

The manuscript would benefit from:
o Clarifying how Z-scores were computed (e.g., globally across all samples or within specific conditions).

o Briefly addressing whether the core biological conclusions remain robust when alternative normalization strategies are applied, thereby enhancing computational rigor.

In the original manuscript, we used Z-score values to display temporal patterns of protein expression in the proteome. These values were calculated individually for each protein based on their relative abundance across the four ages, using the *base* R statistical environment. This procedure has the following advantages: (1) it scales all variables to the same scale, allowing the joined comparison of the relative dynamics of both low-expressed and highly abundant proteins, and (2) it takes into consideration the variance of the data, consequently leading to the mitigation of distortion caused by outliers. Furthermore, representation of temporal patterns of protein expression using K-means offers superior clarity and thus enhances interpretability of timeseries data.

Evaluation of protein levels was only carried out for statistically significant proteins. To ensure that we were only capturing biological variations, batch effects were corrected using the variance stabilization transformation (generalized logarithm), as implemented in the *vsn* Bioconductor package[10]. As an alternative approach, we also attempted quantile normalization; however, the results were inferior to those obtained with *vsn* normalization. To preserve biological variance, the algorithm was applied within predefined experimental groups composed of replicates from the same condition. In addition to Z-scores, we conducted rigorous statistical analysis. Normalized $\log_2$Intensity values for each protein were used as an input for the *limma* R package[11]. The *limma* R package fits a linear model to the $\log_2$ Intensity values for each protein, and uses an empirical Bayes approach to stabilize variance, subsequently increasing the statistical power of the analysis. Details regarding the multiple correction method and the significance threshold applied to each dataset can be found in the "Methods" section of the manuscript. Both steps were important to guarantee that both low- and high-abundance proteins contributed equally to the identified temporal profiles, using the K-means algorithm.

As an additional way to test the robustness of our data, we also carried out a normalization of our data according to cell number. For this, we characterized proteome alterations for an equal number of proliferative and senescent cells (new Figure S1G, H, I). Consistent with the data obtained from protein quantification by Bradford, we observed an increased accumulation of proteins in senescent cells (1245 proteins), while only 129 proteins had their levels decreased. HPA annotation of the dataset showed that proteins localized in the nucleus exhibited a decreased abundance in senescence. Importantly, GO term analysis showed an enrichment of terms such as "chromatin-binding" and "double-stranded DNA binding" for depleted proteins. This brief analysis therefore confirms the patterns described in Figure 1 of the manuscript, where protein levels were used in the normalization of the data. Thus, overall, we feel confident that our analysis reflects the core biological events during senescence.

References

1. Wang, W. & Matunis, M. J. Paralogue-Specific Roles of SUMO1 and SUMO2/3 in Protein Quality Control and Associated Diseases. *Cells* **13**; 10.3390/cells13010008 (2023).

2. Della David, C. *et al.* Widespread protein aggregation as an inherent part of aging in C. elegans. *PLOS Biology* **8,** e1000450; 10.1371/journal.pbio.1000450 (2010).

3. Koyuncu, S. *et al.* Rewiring of the ubiquitinated proteome determines ageing in C. elegans. *Nature* **596,** 285–290; 10.1038/s41586-021-03781-z (2021).

4. Thompson, A. *et al.* Tandem mass tags: a novel quantification strategy for comparative analysis of complex protein mixtures by MS/MS. *Analytical Chemistry* **75,** 1895–1904; 10.1021/ac0262560 (2003).

5. Li, J. *et al.* TMTpro reagents: a set of isobaric labeling mass tags enables simultaneous proteome-wide measurements across 16 samples. *Nat Methods* **17,** 399–404; 10.1038/s41592-020-0781-4 (2020).

6. Zecha, J. *et al.* TMT Labeling for the Masses: A Robust and Cost-efficient, In-solution Labeling Approach. *Molecular & Cellular Proteomics : MCP* **18,** 1468–1478; 10.1074/mcp.TIR119.001385 (2019).

7. Han, C. *et al.* Conformation-specific Antibody Deciphers K27-linked Ubiquitination in Chaperone-Mediated Proteostasis. *bioRxiv*; 10.64898/2025.12.18.695067 (2025).

8. Casella, G. *et al.* Transcriptome signature of cellular senescence. *Nucleic Acids Research* **47,** 7294–7305; 10.1093/nar/gkz555 (2019).

9. Cohn, R. L., Gasek, N. S., Kuchel, G. A. & Xu, M. The heterogeneity of cellular senescence: insights at the single-cell level. *Trends In Cell Biology* **33,** 9–17; 10.1016/j.tcb.2022.04.011 (2023).

10. Huber, W., Heydebreck, A. von, Sültmann, H., Poustka, A. & Vingron, M. Variance stabilization applied to microarray data calibration and to the quantification of differential expression. *Bioinformatics (Oxford, England)* **18 Suppl 1,** S96-104; 10.1093/bioinformatics/18.suppl_1.s96 (2002).

11. Ritchie, M. E. *et al.* limma powers differential expression analyses for RNA-sequencing and microarray studies. *Nucleic Acids Research* **43,** e47; 10.1093/nar/gkv007 (2015).

Revision of manuscript "*Quantitative proteomics reveals coordinated changes in the proteome during replicative senescence*"

Response to the reviewers' comments

We sincerely thank the reviewers for their positive assessment of the revised manuscript and for their constructive and insightful comments. In this new revised version, we have expanded the "Discussion" section, to accommodate literature concerning pathways commonly regulated across different senescent models.

Reviewer #1 (Remarks to the Author):

The authors have properly addressed my comments and solidified their claims regarding proteoasome activity (new figures 5, G and H). They also expanded their exploration of SUMOylation in the context of cell senescence (new figures 4, C and D). The new data is discussed in the revised manuscript.
I get the impression that they also comprehensively addressed the critique of referee #2.
In conclusion, I think that the manuscript is much clearer and should be published in its current form.

Reviewer #2 (Remarks to the Author):

I would like to thank the authors for their comprehensive and constructive response to the review comments. The revised manuscript has been significantly strengthened. However, one major issue regarding the generality of the findings across different senescence models remains insufficiently resolved:
In their response to the query regarding the generalizability of the findings (Reviewer #2, Major Comment 6), the authors argue for the existence of a "core senescence program" by referencing transcriptomic and single-cell RNA-seq literature and have updated the discussion on page 26. While this conceptual framing is valuable, it relies almost exclusively on transcript-level data. Given that this study is fundamentally rooted in quantitative proteomics, protein solubility profiling, and post-translational modifications, transcriptomic conservation does not automatically translate to proteomic or PTM-level conservation. To fully resolve this point, the authors should further refine the discussion in the manuscript to:

1. Discuss how their specific proteomic, solubility, and PTM (ubiquitylome/SUMOylome) signatures compare or contrast with existing proteomic datasets of other senescence models, such as oncogene-induced senescence (OIS) or stress-induced premature senescence (SIPS), rather than relying solely on mRNA-level literature.

In the revised manuscript, we have added comparative information regarding proteomic signatures across senescence models (page 26-27).

2. Explicitly address the limitation that a transcriptionally defined "core senescence program" may not fully capture the conservation or divergence of the senescent proteome and PTM networks across different cellular contexts and induction methods.

Evidence for a core senescence response has so far come from bulk and single-cell transcriptomic analyses. In recent years, advances in mass spectrometry have improved the detection of proteomes and post-translational modifications, offering new opportunities to characterize cellular states in greater depth.

However, proteomics studies of context-specific senescence signatures remain limited and often rely on pairwise comparisons between proliferative and senescence states, thereby missing dynamic proteome alterations. Nonetheless, existent datasets indicate recurrent regulation of specific pathways and protein complexes across senescence models, although not always in the same direction. This may support the existence of mechanisms that are commonly regulated at the proteomic level across senescence models. We have included this point in the Discussion section of the revised manuscript, on pages 26-27.

Once this final point is adequately addressed in the text, the manuscript should be well-suited for publication.
