# Supplementary material for: Quantitative proteomics reveals coordinated changes in the proteome during replicative senescence
Source: Nat Commun. 2026 Sep 16;17:9877. doi: 10.1038/s41467-026-77686-8 (PMC13582980; doi:10.1038/s41467-026-77686-8)
Supplement: Supplementary file 3 [file 41467_2026_77686_MOESM1_ESM.pdf]

Quantitative proteomics reveals coordinated changes in the proteome during replicative senescence

Supplementary Information

Nádia Da Silva Fernandes[1,2], Fridolin Kielisch[1], Amitkumar Fulzele[1], Sivarajan Karunanithi[1], Justus F. Gräf[1], Jia-Xuan Chen[1], Christian Behl[2], Helle D. Ulrich[1*], Petra Beli[1,3*]

[1]Institute of Molecular Biology (IMB), 55128 Mainz, Germany

[2]Institute of Pathobiochemistry, The Autophagy Lab, University Medical Center of the Johannes Gutenberg University, 55128 Mainz, Germany

[3]Institute of Developmental Biology and Neurobiology (IDN), Johannes Gutenberg University, 55128 Mainz, Germany

[*]Correspondence should be addressed to Helle D. Ulrich (H.Ulrich@imb-mainz.de) and Petra Beli (petrbeli@uni-mainz.de)

This PDF file includes:

Supplementary Figures and Legends 1 to 5

Supplementary Fig. 1

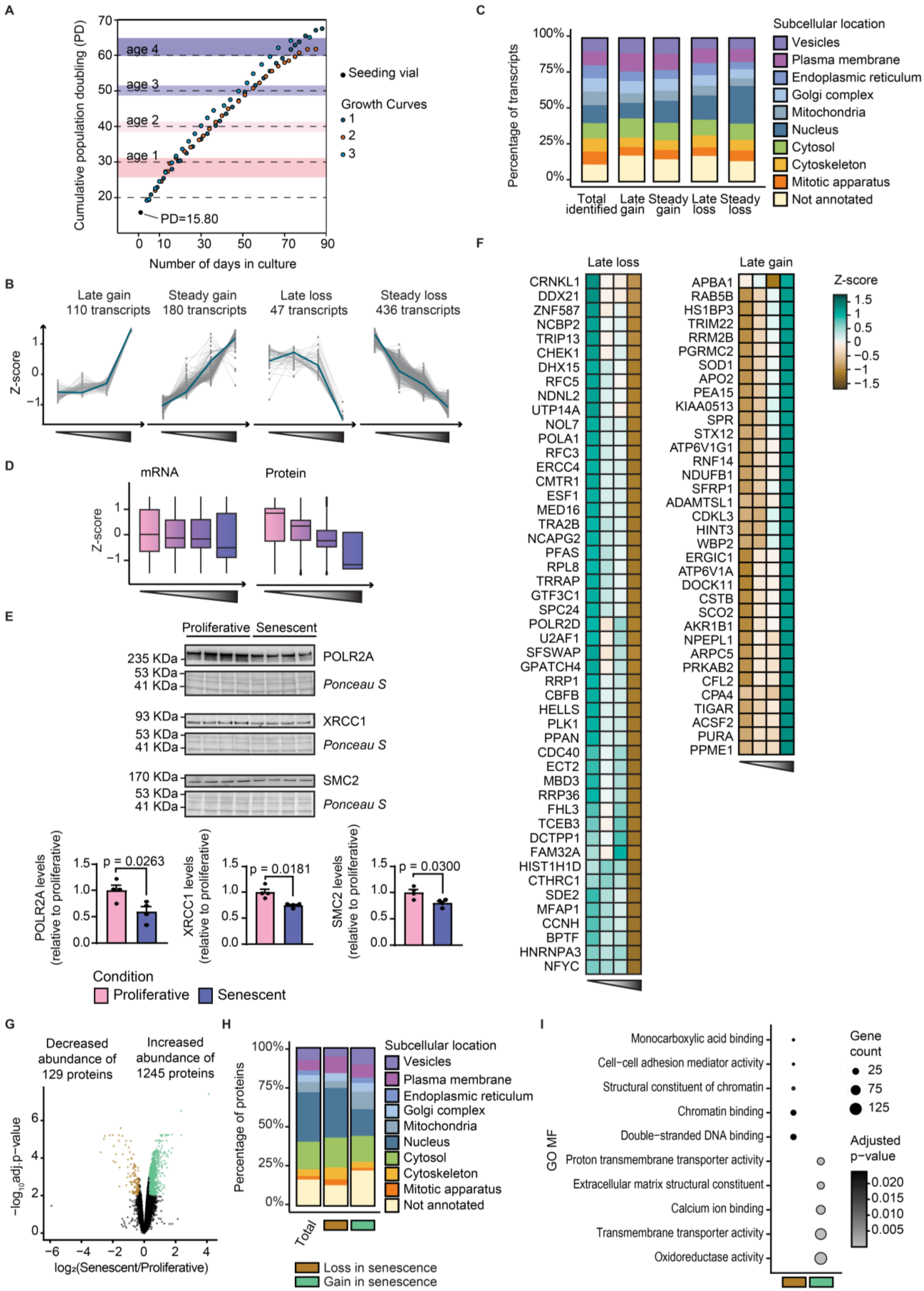

Supplementary Fig. 1 | Transcriptome and proteome remodeling during replicative senescence. (A) Dot plot displaying the three growth curves established for this study (numbered from one to three). The initial seeding population is highlighted, and the specific population doublings selected for proteomics analysis are indicated in green. (B) Transcripts found to be differentially regulated were clustered using the K-means algorithm, showing distinct temporal patterns. (C) Stacked bar plot showing the percentage of transcripts significantly regulated by subcellular localization of the corresponding proteins according to the Human Proteome Atlas annotation and the clusters defined in panel A. (D) Box plot showing Z-scores for all quantified mRNA transcripts encoding nuclear-localized proteins and for all quantified nuclear proteins, annotated according to the Human Proteome Atlas. Bottom border, interior line, and top border of the box represent the first quartile, median and third quartile, respectively. Whiskers extend to the minimum and maximum values within 1.5× IQR, with outliers being represented as individual points. (E) Top: Representative western blot images used for the quantification of POLR2A, XRCC1 and SMC2 levels in proliferative and senescent cells. Ponceau S staining served as loading control and for normalization. Bottom: Bar plots displaying relative levels of POLR2A, XRCC1 and SMC2 in proliferative and senescent cells. Error bars display SEM, and p-values were calculated using Welch's statistics. (GC1, n= four age replicates). (F) Heatmap showing Z-scores of nuclear proteins significantly lost (left) or gained (right) only at later stages during senescence. (G) Volcano plot displaying all quantified proteins when evaluating two hundred thousand proliferative or senescent cells. Significantly upregulated and downregulated proteins are highlighted in green and brown, respectively. (GC2, n= four age replicates). (H) Stacked bar plot shows the distribution of protein main subcellular localization as defined by the Human Proteome Atlas for all proteins quantified and significantly regulated when analyzing equal numbers of proliferative and senescent cells. (I) Dot plot displaying GO MF terms enriched for proteins found to be increased or decreased in abundance in senescent cells when analyzing equal numbers of proliferative and senescent cells. Only terms with adjusted p-value < 0.05 (hypergeometric test with Benjamin-Hochberg false discovery correction) were considered.

Supplementary Fig. 2

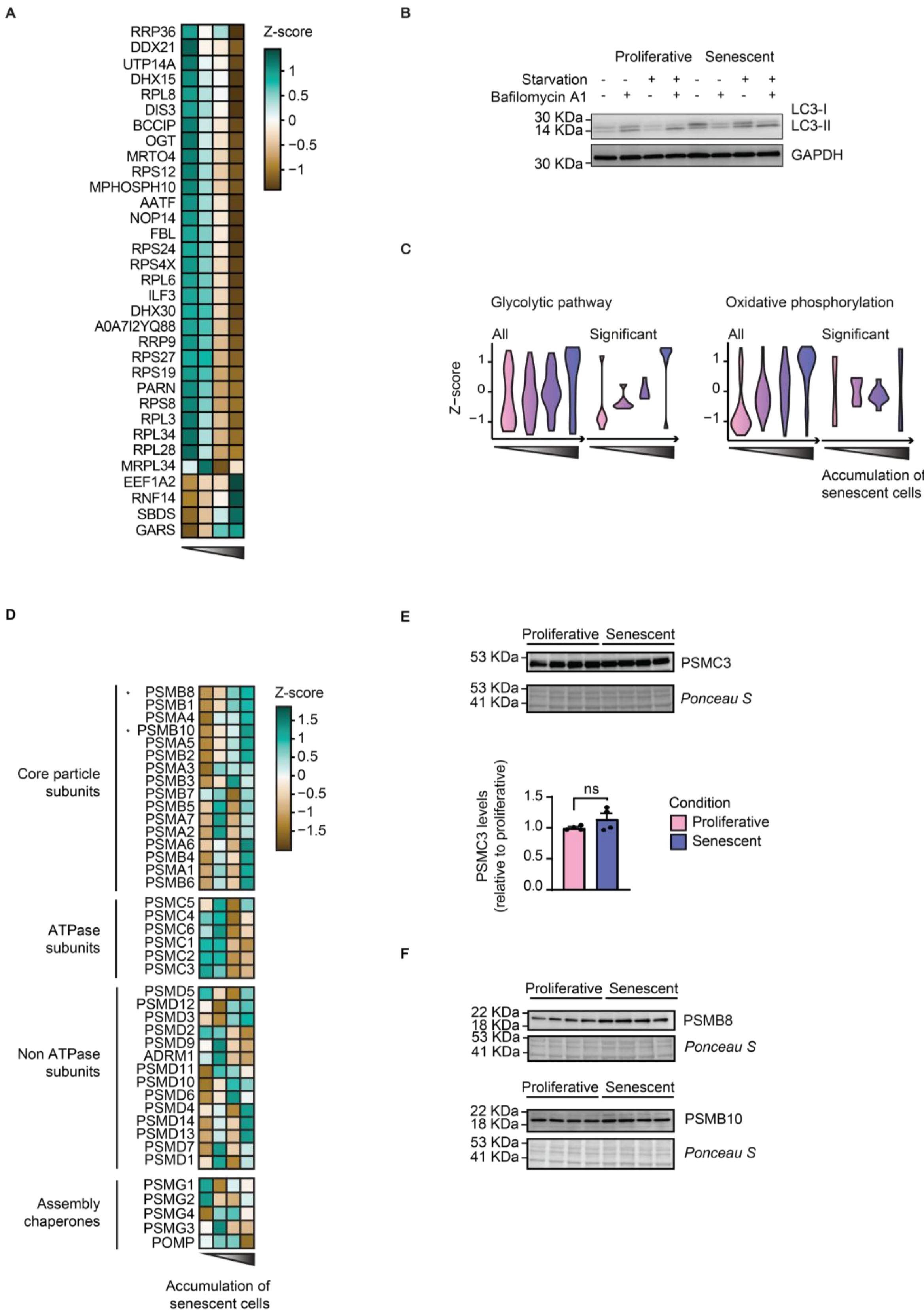

Supplementary Fig. 2 | Regulation of translation and proteasome components in senescence. (A) Heatmap showing the Z-scores of significantly regulated proteins associated with translation. (B) Representative western blot images used for the quantification of LC3B-I and LC3B-II, for the assessment of autophagy flux in proliferative and senescent cells. Proliferative and senescent cells were exposed for 2 hours to 2.5 µM of Bafilomycin A1 in control and starvation conditions. Detection of GAPDH served for normalization. (GC4, n= three age replicates). (C) Violin plots showing the trends of all quantified proteins involved in the glycolytic and oxidative phosphorylation pathways, respectively, and the subset of proteins found to be significantly regulated during senescence. (D) Heatmap showing the Z-scores of all quantified proteasomal subunits, subdivided into core proteasomal subunits, ATPase subunits, non-ATPase subunits and assembly chaperones. (E) Top: representative western blot image used for the quantification of PSMC3 levels in proliferative and senescent cells. Bottom: bar plots displaying relative PMSC3 levels. Ponceau S staining served as loading control. The error bars represent SEM. Statistical analysis was performed using multiple unpaired t-test Welch's correction. (GC1, n= four age replicates). (F) Representative western blot images used for the quantification of relative PSMB8 and PSMB10 levels in proliferative and senescent conditions. Ponceau S staining served as loading control and for normalization. (GC1, n= four age replicates).

Supplementary Fig. 3

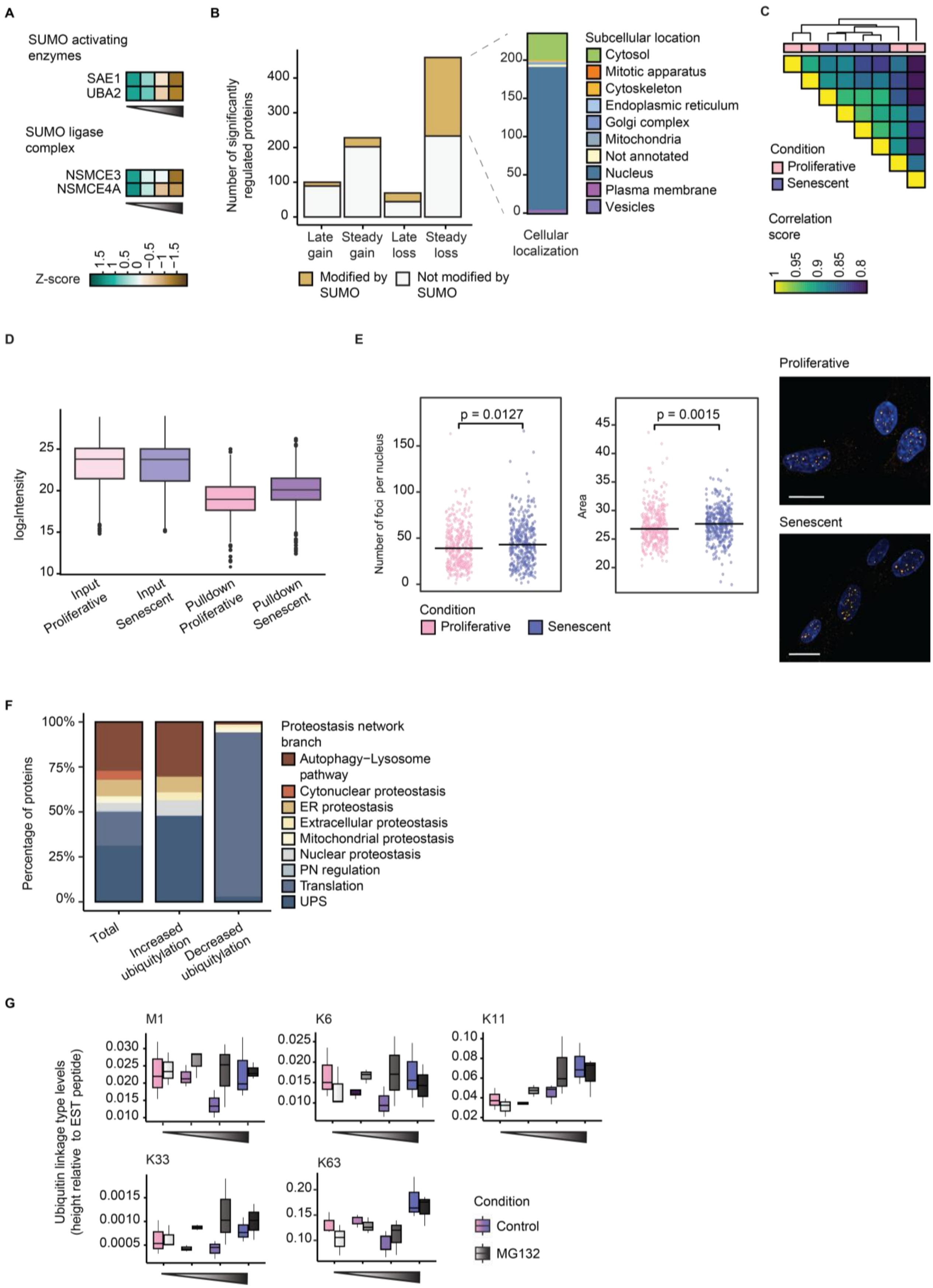

Supplementary Fig. 3 | Changes in SUMOylation and ubiquitylation during replicative senescence. (A) Heatmap showing the Z-scores of SUMO-specific E1 and E3 ligases found to be significantly regulated in senescence. (B) Left: stack plot showing the percentage of known SUMO substrates found to be differentially regulated in senescence. Right: stack plot displaying the relative distribution of protein main subcellular localizations for known SUMO substrates within the steady loss cluster. (C) Heatmap displaying hierarchical clustering and Pearson correlation scores for SUMO2/3 pulldown samples. (D) Box plots displaying total $\log_2$Intensities for the input and SUMO2/3 pulldown samples evaluated by mass spectrometry. Bottom border, interior line, and top border of the box represent the first quartile, median and third quartile, respectively. Whiskers extend to the minimum and maximum values within 1.5× IQR, with outliers being represented as individual points. (E) Left: Dot plots showing the number of PML bodies per nucleus and mean area of detected foci for proliferative and senescent cells. Black lines represent median values. Statistical analysis was done using the Wilcoxon rank sum test with continuity correction. (GC2, n= four age replicates; a total of 380 nuclei were analyzed per condition). Right: Representative images for nuclear PML foci quantified for proliferative and senescent cells. Scale bars correspond to 20 µm. (F) Stack plot representative of the distribution of proteostasis network members with significantly changed ubiquitylation status in senescence (G) Box plot showing the SureQuant™ targeted quantification of M1, K6, K33, K63 and EST peptides upon senescence in control and MG132-treated conditions. Bottom border, interior line, and top border of the box represent the first quartile, median and third quartile, respectively. Whiskers extend to the minimum and maximum values within 1.5× IQR, with outliers being represented as individual points (GC4, n= three age replicates, seven transitions quantified per age replicate).

Supplementary Fig. 4

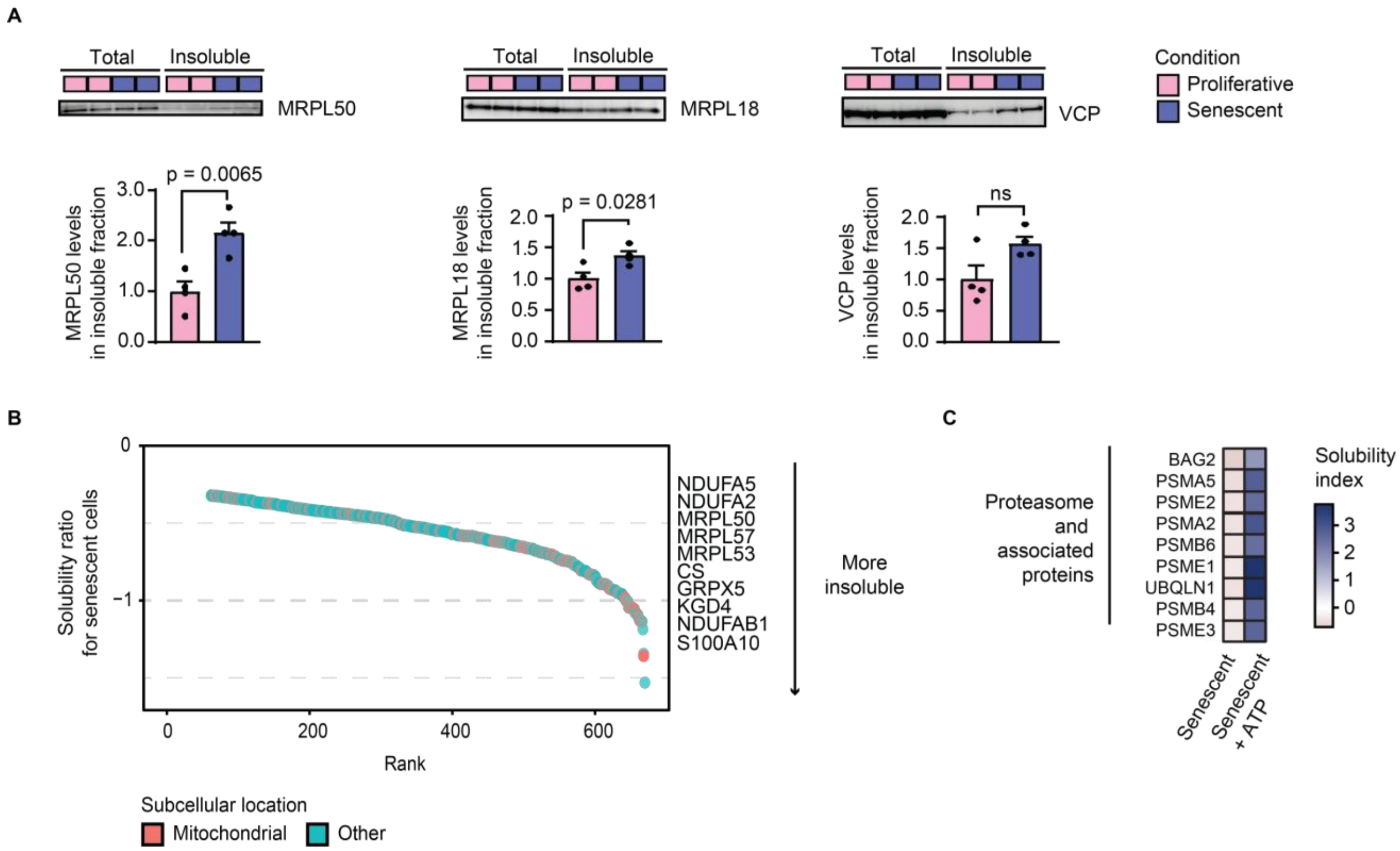

Supplementary Fig. 4 | Protein solubility changes during replicative senescence. (A) Top: Representative western blot images used for the quantification of MRPL50, MRPL18 and VCP for the assessment of alterations in their solubility in proliferative and senescent cells. Insoluble fraction values were normalized to the corresponding total protein levels. Bottom: bar plots displaying relative levels of MRPL50, MRPL18 and VCP in insoluble fractions for proliferative and senescent cells. Error bars display SEM, and p-values were calculated using Welch's statistics. (GC1, n= four age replicates) (B) Rank plot of all proteins that lose solubility in senescence, distinguishing mitochondrial from non-mitochondrial proteins. The top ten mitochondrial proteins to lose solubility in senescence are indicated on the right of the plot. (C) Heatmap showing alterations of the solubility index for proteasome subunits and associated proteins that lose solubility in senescence.

Supplementary Fig. 5

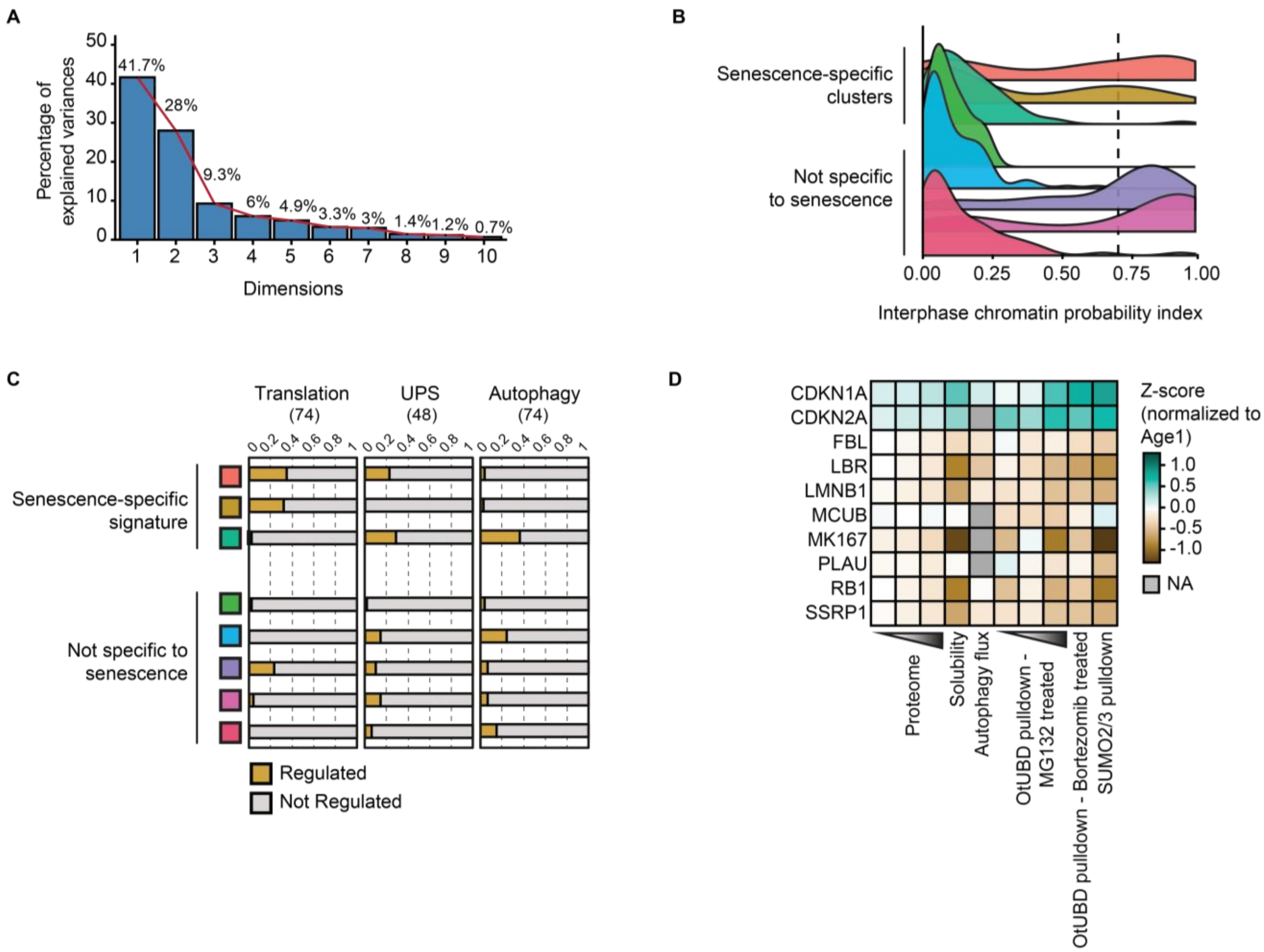

Supplementary Fig. 5 | Comparative analysis of the senescent proteome to different cellular stress conditions. (A) Bar plot showing the percentage of variance explained by each PC as defined in Figure 7A. (B) Distribution of the interphase chromatin probability index as reported by Kustatscher *et al.*[15], organized by the protein clusters defined in Figure 7C. (C) Bar plot showing the percentage of proteins associated with translation, UPS and autophagy that are significantly regulated during senescence within the clusters defined in Figure 7C. (D) Heatmap showing the relative abundance of canonical senescence markers identified across different datasets. Protein levels are presented as Z-scores of fold-change values, normalized to proliferative (age 1) cells.
