# Supplementary material for: Quantitative proteomics reveals coordinated changes in the proteome during replicative senescence
Source: Nat Commun. 2026 Sep 16;17:9877. doi: 10.1038/s41467-026-77686-8 (PMC13582980; doi:10.1038/s41467-026-77686-8)
Supplement: Supplementary file 4 [file 41467_2026_77686_MOESM2_ESM.docx]

**Description of Additional Supplementary Files**

Supplementary Data 1 | Proteome remodeling during senescence The excel file contains all quantified proteins in the TMT-based time series experiment, used for the profiling of proteome alterations during replicative senescence. Information regarding the adjusted p-values for the pairwise empirical Bayes moderated t-test and the ANOVA statistical analysis were added, as well as annotations for statistical significance. The dataset is annotated according to the Human Protein Atlas Annotation for main subcellular localization information and to the proteostasis consortium database for information regarding the proteostasis network branch. Additionally, this file contains all quantified proteins for the proteome analysis of proliferative and senescent cells when using equal cell numbers for the analysis.

Supplementary Data 2 | Transcriptome remodeling during senescence The excel file contains all quantified transcripts during replicative senescence across four ages. Adjusted p-values derived from the pairwise empirical Bayes moderated t-test comparison and ANOVA statistical analysis were added, as well as annotations for statistical significance.

Supplementary Data 3 | Alterations in protein SUMOylation during senescence The excel file contains all quantified SUMO2/3-modified proteins in proliferative and senescent conditions. Levels of SUMOylated proteins enriched in the pulldown are presented with and without normalization to the proteome. Mean log2Intensity and log2(Senescent/Proliferative) values are presented, as well as annotations for statistical significance (pairwise empirical Bayes moderated t-test values).

Supplementary Data 4 | Transitions used in the quantification of ubiquitin and ubiquitin linkages in SureQuantTM experiments The excel file contains a list of transitions that were used for the quantification of ubiquitin and distinct ubiquitin linkages in the SureQuantTM experiments. Targeted acquisition was performed for OtUBD pulldown samples in control and MG132 treated conditions, as well as for insoluble and total fractions of proliferative and senescent IMR90 cells.

Supplementary Data 5 | Quantification of ubiquitin and ubiquitin linkages during senescence The excel file contains the peak height values used for the quantification of ubiquitin and different ubiquitin linkages in the SureQuantTM experiments. Targeted acquisition was performed for OtUBD pulldown samples in control and MG132-treated conditions, as well as for insoluble and total fractions of proliferative and senescent IMR90 cells. The dataset includes information regarding the adjusted p values calculated from the pairwise empirical Bayes moderated t-test.

Supplementary Data 6 | Alterations in protein ubiquitylation during senescence The excel file contains all quantified ubiquitylated proteins during replicative senescence across four ages. Levels of ubiquitylated proteins pulled down by the OtUBD probe were normalized to proteome levels. Mean log2Intensity and log2(Senescent/Proliferative) values are presented, as well as annotations for statistical significance (adjusted p values calculated from the pairwise empirical Bayes moderated t-test).

Supplementary Data 7 | Alterations in degradative protein ubiquitylation during senescence The excel file contains all quantified ubiquitylated proteins upon proteasomal inhibition by MG132 or Bortezomib, measured across the four ages. Levels of ubiquitylated proteins pulled down by the OtUBD probe were normalized to proteome levels. For all protein groups, mean log2Intensity, log2(MG132 treated/control) and log2(Bortezomib treated/control) values are presented, as well as annotations for statistical significance (adjusted p-values calculated from the pairwise empirical Bayes moderated t-test).

Supplementary Data 8 | Alterations in protein solubility during senescence The excel file contains the mean solubility ratio for all quantified proteins in the solubility experiment in proliferative and senescent conditions. The adjusted p-values obtained upon pairwise empirical Bayes moderated t-test comparison of proliferative and senescent cells, as well as annotations describing the solubility state of each protein are presented.

Supplementary Data 9 | Exposure of IMR90 proliferative cells to distinct stressors The excel file contains the Z-scores of all proteins distinctly regulated during cellular senescence, and their Z scores upon exposure to distinct stressors, including Inflachromene, starvation, hydrogen peroxide, DMAG-17, Bafilomycin A1, MG132 and Bortezomib treatments. The dataset also includes information regarding PCA decomposition and the clusters defined using the Louvain algorithm.
